# Biopsychosocial impact of high levels of trait anxiety on family caregivers in the end-of-life palliative care setting

**DOI:** 10.1371/journal.pone.0307349

**Published:** 2025-03-18

**Authors:** Anastasia Maria Grumeth, Hans-Bernd Rothenhäusler, Sabrina Mörkl, Jolana Wagner-Skacel, Elisabeth Sciri, Andreas Baranyi

**Affiliations:** 1 Department of Psychiatry, Psychosomatics and Psychotherapeutic Medicine, Division of Psychiatry and Psychotherapeutic Medicine, Graz, Austria; 2 Department of Psychiatry, Psychosomatics and Psychotherapeutic Medicine, Division of Medical Psychology, Psychosomatics and Psychotherapeutic Medicine, Graz, Austria; 3 Department of Internal Medicine, University Palliative Care Facility, Graz, Austria; Medical University of Vienna, AUSTRIA

## Abstract

**Introduction:**

Caring for a family member with a palliative diagnosis at home is physically and mentally stressful. This prospective study explores the emotional and physical burdens experienced by family caregivers in end-of-life palliative care settings, particularly focusing on those caregivers with high levels of pre-existing trait anxiety. The following hypotheses were examined: 1.) Family caregivers with high levels of trait anxiety suffer from high levels of anxiety, stress, burnout symptoms, insomnia, daytime sleepiness, physical complaints, health-related anxiety and resentments. 2.) Caregivers with a high level of trait anxiety are less resilient and receive less social support. They are more likely to use inappropriate strategies in the context of emotional regulation and work-related behavior. 3.) The support provided by the mobile palliative care team reduces stress and improves the quality of sleep of the caregiving relatives.

**Methods:**

Forty-seven caregivers participated, with assessments conducted at two time points: before the mobile palliative care team’s intervention and six weeks later. Data collection included measures such as the State-Trait Anxiety Inventory (STAI), Whiteley Index (WI), Perceived Stress Scale (PSS-10), Maslach Burnout Inventory (MBI-HSS), Insomnia Severity Index (ISI), Epworth Sleepiness Scale (ESS), Giessen Complaints Questionnaire, Berner Bitterness Inventory (BVI), Brief Resilience Coping Scale, FEEL-E for emotional regulation, AVEM, and the MOS Social Support Survey.

**Results:**

High levels of trait anxiety among caregivers were significantly associated with increased anxiety, stress, emotional exhaustion, depersonalization, insomnia, and feelings of resentment. Notably, maladaptive emotional regulation strategies were also prevalent. Intervention by the mobile palliative care team significantly reduced stress and insomnia, showing benefits across all levels of trait anxiety.

**Conclusion:**

Family caregivers with high trait anxiety face significant challenges in end-of-life palliative care settings, indicating a crucial need for early identification and comprehensive biopsychosocial support to mitigate adverse emotional and physical health outcomes.

## 1. Introduction

### 1.1. Overview and introduction

Receiving a life-limiting diagnosis is often a traumatic experience not only for the affected person but also for their caring relatives, which changes the entire family's life situation. Nowadays, severe illnesses and the associated death can no longer be experienced directly, as care is often institutionalized, and the act of dying usually takes place in medical facilities such as hospitals or palliative care units [1–3]. The conscious decision to care for a seriously ill family member at home often takes immense demand on relatives. It can lead to psychological stress and impairments to the caregiver's health. Frequently, feelings of worry, sadness, and powerlessness arise. Thus, family caregivers often suffer from anxiety and burnout symptoms as they are exposed to permanently high levels of stress [4]. It is known that family caregivers of palliative patients have a more impaired quality of life and more physical complaints when compared to family members who care for relatives with curable illnesses. The social pressure to meet one's requirements and that of the person being cared for contributes to long-term stress [5].Caregiving itself is experienced as a multidimensional and multifaceted task bycaregivers, which is influenced by several factors. Many relatives do not only assist ill family members physically by assisting with everyday tasks such as washing and dressing, but they also often provide high levels of psychological support. Due to the time-consuming physical and mental support, many family caregivers have to reduce their leisure time activities. If there is also a lack of resources in the context of information or social support, this might reduce the carer's well-being and the quality of care provided to the patient [6]. For many relatives, the primary focus is on an incurable, progressive illness and the associated deterioration in a family member's state of health, as well as the constant challenge of coping with progressive and newly emerging symptoms. In addition, many family members are also confronted with making essential decisions regarding end-of-life care. The extent of a person's pre-existing trait anxiety might have an impact on the perception of anxiety in the caring situation [7]. Trait anxiety can be seen as a consistent part of a person's personality, whereas state anxiety is a transitory emotional response involving unpleasant feelings caused by a specific situation.

### 1.2. Aims of the study

High levels of pre-existing trait anxiety might affect the mental and physical health of family caregivers in the end-of-life palliative care setting at home. The following hypotheses were examined in this prospective study:

- Family caregivers with high levels of trait anxiety suffer from high levels of anxiety, stress, burnout symptoms, insomnia, daytime sleepiness, physical complaints, health-related anxiety and resentments.
- Caregivers with a high level of trait anxiety are less resilient and receive less social support. They are more likely to use inappropriate strategies in the context of emotional regulation and work-related behavior.
- The support provided by the mobile palliative care team reduces stress and improves the quality of sleep of the caregiving relatives.

## 2. Methods

### 2.1. Procedures

This prospective study was conducted in collaboration with the Division of Psychiatry and Psychotherapeutic Medicine, Medical University of Graz, and the Mobile Palliative Care Team Graz/Graz-Umgebung.

The Mobile Palliative Care Team Graz/Graz-Umgebung in Austria offers specialized care focused on the needs of terminally ill patients. The team's tasks include pain relief and symptom control, holistic care and coordination of it, support for relatives, enabling patients to die with dignity and also counselling and support with ethical and legal issues. The typical members of a mobile palliative care team are palliative care physicians/doctors, palliative care nurses, social workers, psychologists/psychotherapists, counselors (e.g., priests, religious communities), physiotherapists, occupational therapists, dieticians and volunteer helpers.

All participants were family caregivers of patients with an end-of-life palliative care setting and were recruited by the mobile palliative care team Graz/ Graz-Umgebung. The data collection started on the 17^th^ of May 2021 and ended on the 14^th^ of April 2022. During the study period, the palliative care team Graz/ Graz-Umgebung was in contact with 153 palliative patients. Of these 153 patients, 46 died before their caregiving relatives were invited to participate in the study. 60 caregiving relatives declined to participate in the study. The main reason given was the time required to complete the questionnaires. The study sample subsequently consisted of 47 family caregivers. In terms of sociodemographics, the participating family caregivers did not differ significantly from the non-participating family caregivers.

The inclusion criteria were as follows: 1.) family caregivers of patients with an end-of-life palliative care setting, 2.) age between 18 and 90 years. Family caregivers were excluded if they had a known pre-existing psychiatric disease, were unable to give consent, or did not meet the inclusion criteria.

There were two measurement points at which family caregivers received psychometric questionnaires:

1. Baseline: The first measurement point was at the beginning of the end-of-life support provided by the mobile palliative care team Graz/ Graz Umgebung, Austria. At that measurement point, family caregivers were given the following psychometric questionnaires: State-Trait Anxiety Inventory (STAI), Whiteley Index (W.I.), Perceived Stress Scale (PSS-10), Trier Inventory on Chronic Stress (TICS), Maslach Burnout Inventory - Human Services Survey (MBI-HSS), Insomnia Severity Index (ISI), Epworth Sleepiness Scale (ESS), Giessen complaint Questionnaire, Berner Bitterness Inventory (BVI), Brief Resilience Coping Scale (BRCS), FEEL-E, AVEM-44. and the MOS Social-Survey-Support.
2. Re-evaluation: Most patients with end-of-life support die within the first few weeks, so the time of re-evaluation was set at 6 weeks after the beginning of end-of-life support by the mobile palliative care team Graz/ Graz-Umgebung. Family caregivers were given the following psychometric questionnaires: Perceived Stress Scale (PSS-10), Trier Inventory on Chronic Stress (TICS), Insomnia Severity Index (ISI), and the MOS Social-Survey-Support.

This study was approved by the ethics committee of the Medical University of Graz (protocol- no: EK33-332 ex 20/21). Before voluntary participation, family caregivers signed an informed consent form.

### 2.2. Measures

#### 2.2.1. Socio-demographic data.

All caregivers were asked about their gender, age, the caring level, and the primary diagnoses of the affected family member. In addition, the type of support provided during the caring situation was also explored.

#### 2.2.2. Psychometric questionnaires.

**Anxiety:**

- **State-Trait Anxiety Inventory (STAI):** The State-Trait Anxiety Inventory (STAI) by Spielberger et al. [8] is a self-report questionnaire with which state anxiety and trait anxiety (general anxiety) are assessed. Based on a median splitting (median =  40), the study participants were divided into a group with higher and a group with lower trait anxiety. The median calculated in this study also corresponds exactly to the cut-off recommended by Addolorato et al. [9].
- **Whiteley Index (W.I.):** According to Pilowsky [10], the WI is suitable as a screening method for body-related and health-related anxieties, including hypochondriacal symptoms.

**Stress:**

- **Perceived Stress Scale (PSS-10):** The PSS-10 by Cohen et al. [11] measures the quantitative extent of acute stress exposure in the current caregiving situation.
- **Trier Inventory on Chronic Stress (TICS):** The TICS by Schulz, Schlotz and Becker [12] records various areas of chronic stressors.

**Burnout symptoms:**

- **Maslach Burnout Inventory - Human Services Survey (MBI-HSS):** The MBI – HSS by Maslach et al. [13] is a self-assessment instrument for recording burnout symptoms in the caring sector. It includes three burnout categories: emotional exhaustion, personal performance and depersonalization. Depersonalization in the context of burnout means an unemotional and impersonal way of dealing with the recipients, e.g., the patients being cared for.

**Sleeping disorders:**

- **Insomnia Severity Index (ISI):** The ISI by Bastien et al. [14] determines the severity of a sleep disorder.
- **Epworth Sleepiness Scale (ESS):** The ESS, according to Johns [15], is a scale that measures daytime sleepiness.

**Psychosomatic complaints:**

- **Giessen Complaint Questionnaire (GBB-24):** The GBB-24 by Brähler et al. [16] records the psychosomatic correlation or partial correlation of physical complaints.

**Resentments:**

- **Berner Bitterness Inventory (BVI):** The Berner Bitterness Inventory (BVI), according to Znoj [17], measures resentments in the private sphere.

**Resilience:**

- **Brief Resilience Coping Scale (BRCS):** The BRCS by Sinclaire et al. [18] assesses the extent to which resilience and coping strategies are present. Resilience is understood as adaptability and as a process in which people respond to problems and changes by adapting their behavior.

**(Mal-)adaptive strategies in the context of emotional regulation:**

- **FEEL-E:** The FEEL-E by Grob et al. [19] is a questionnaire that measures the adaptivity or maladaptivity of emotion regulation strategies. Adaptive strategies are problem-oriented action, acceptance, cognitive problem solving, reappraisal, mood elevation, forgetting, and maladaptive strategies are withdrawal, self-devaluation, giving up, perseveration, catastrophizing, and blaming others.

**Work-related patterns of behavior and experience:**

- **AVEM-44:** Work-related behavior and experience patterns were assessed with the AVEM 44 by Schaarschmidt et al. [20]. It allows statements about health-promoting or health-threatening behaviors and experiences when coping with work demands.

**Social support:**

- **MOS Social-Survey-Support:** The MOS Social-Survey-Support [21] includes the amount of emotional, physical, and affectionate support and the extent of positive social interaction and distraction. In addition, the overall level of support is examined in the MOS.

### 2.3. Statistical analysis

Descriptive data are presented as mean and standard deviation (S.D.). In the case of normally distributed continuous measurements, group differences were analyzed using t-tests or, in the case of repeated measurements, using RM-ANOVAs and RM-ANCOVA. Correlative associations were analyzed using Pearson’s correlation coefficients. All statistical tests were two-sided (p <  0.05). All statistical analyses were performed with SPSS 25.0 for Windows.

## 3. Results

### 3.1. Socio-demographic characteristics

47 family caregivers (30.7%; 11 men (23,4%) and 36 women (76,6%) were enrolled. Twenty-one participants (44.7%) were employed, 21 caregivers (44.7%) were retired, 3 people (6.38%) did something different and 2 (4.26%) participants did not answer that question. The primary diagnoses of the palliative patients were cancer (59.6%), neurological (8.5%), lung (6.38%) and heart (2.13%) diseases. Palliative care support was mainly provided by family members (89.4%), in the other cases, by close friends.

#### 3.1.1. Impact of trait anxiety in the end-of-life palliative care setting.

**Trait anxiety in caregivers:** Based on a median splitting (median =  40), the family caregivers were divided into a group with higher and a group with lower trait anxiety. The mean STAI - Trait anxiety score for caregivers with a high level of trait anxiety was 50.81 (*SD* = ±6.38), while the mean STAI - Trait anxiety score for caregivers with a low level of trait anxiety was 33.52 (*SD* = ±5.11). 

Caregivers with a greater extent of trait anxiety were, on average 57.38 years old (*SD* = ±15.47), whereas participants with less trait anxiety were, on average, 59.13 years old (*SD* = ±10.95; *T*  =  0.44; df = 42; *p* = 0.665; Cohen’s d = 0,132; CI: LL = -0,461, UL = -0,723).

#### 3.1.2. Trait anxiety and anxiety in the caregiving situation.

Caregivers with high levels of pre-existing trait anxiety had an average of 56.43 points (*SD* = ±9.54) in the STAI-State, whereas participants with low trait anxiety scored an average of 42.78 points (*SD* = ±10.41) in the STAI-State (*T*  =  -4.52; *df* = 42; *p* < 0.01; Cohen’s d = -1,364; CI: LL = -2,016, UL = -0,698). This means that caregivers with high trait anxiety suffered from high state anxiety in the caring situation.

#### 3.1.3. Trait anxiety and caregivers’ health-related anxiety.

Caregivers with high levels of trait anxiety had a mean score of 4.48 (*SD* = ±2.46) on the Whiteley Index (W.I.), whereas caregivers with low trait anxiety had a mean score of 2.57 points (*SD* = ±1.97). This means that participants with high levels of trait anxiety suffered more from health-related anxiety than those with low trait anxiety (*T* = -2.85; *df* = 42; *p* = 0.007; Cohen’s d = -0,861; CI: LL = -1,476, UL = -0,237).

### 3.2. Stress

#### 3.2.1. Perceived chronic stress before the beginning of the palliative care situation and trait anxiety.

In the TICS, caregivers with high levels of trait anxiety had an average score of 16.20 (*SD* = ±7.36), whereas caregivers with low trait anxiety had a mean score of 19.55 points (*SD* = ± 7.88; *T* = 1.42; *df* = 40; *p* = 0.16; Cohen’s d = 0,438; CI: LL = -178, UL = -1,048). Thus, caregivers with high trait anxiety had a similarly perceived chronic stress load before the beginning of the palliative care situation as caregivers with low trait anxiety.

#### 3.2.2. Trait anxiety and stress levels in the caregiving situation.

Caregivers with high levels of trait anxiety had an average score of 23.86 (SD = +5.04) on the PSS-10, whereas caregivers with low trait anxiety had a lower mean score of 16.04 points (SD = +4.13; T = -5.64; df = 42; p < 0.01; Cohen’s d = -1,704; CI: LL = -2,390, UL = -1,002). This shows that caregivers with high levels of trait anxiety suffered from higher levels of stress in the caring situation.

### 3.3. Burnout symptoms, emotional exhaustion, depersonalization and personal performance

#### 3.3.1. Burnout symptoms and stress in the caregiving situation.

Analyses showed a significant positive correlation between stress levels (PSS-10) in the caregiving situation and burnout symptoms (MBI-HSS; emotional exhaustion: Pearson’s *r* = 0.612; *p* < 0.001; depersonalization: Pearson’s *r* = 0.501; *p* < 0.001).

#### 3.3.2. Trait anxiety and emotional exhaustion.

The palliative caregivers with high levels of trait anxiety had an average score of 10.47 (*SD* = ±2.47) on the emotional exhaustion scale (MBI-HSS), whereas caregivers with low trait anxiety had a mean score of 9.99 points (*SD* = ± 2.08). Family caregivers with high trait anxiety suffer from a higher level of emotional exhaustion than those with low trait anxiety. (*T* = -3.54; *df* = 39; *p* = 0.001; Cohen’s d = -1,115; CI: LL = -1,773, UL = -0,444).

#### 3.3.3. Trait anxiety and depersonalization.

Caregivers with high levels of trait anxiety had an average score of 4.94 (*SD* = ± 4.17) on the depersonalization scale (MBI-HSS), whereas caregivers with low trait anxiety had a mean score of 1.22 points (*SD* = ± 1.44). Family caregivers with high trait anxiety suffer from a higher degree of depersonalization than people with low trait anxiety (*T* = -3.63; *df* = 20.21; *p* = 0.002; Cohen’s d = -1,261; CI: LL = -1,931, UL = -0,577).

#### 3.3.4. Trait anxiety and personal performance.

Caregivers with high levels of trait anxiety had an average score of 37.94 (*SD* = ± 8.22) on the personal performance scale (MBI-HSS), whereas caregivers with low trait anxiety had a mean score of 38.91 points (*SD* = ± 6.82). No significant difference in personal performance was found (*T* = 0.41; *df* = 39; *p* = 0.682; Cohen’s d = 0,130; CI: LL = -0,488, UL = -0,746).

### 3.4. Insomnia and daytime sleepiness

#### 3.4.1. Insomnia and stress in the caregiving situation.

A significant correlation was found between an increased occurrence of insomnia (Insomnia Severity Index; ISI) and increased stress levels in the caregiving situation (PSS-10; Pearson correlation coefficient *r* = 0.480; *p* < 0.01). It can therefore be assumed that the occurrence of sleeping disorders is associated with stress levels in the caregiving situation. No significant correlation was found between daytime sleepiness (ESS) and stress levels (PSS-10; Pearson’s *r* = 0.286; *p* = 0.057).

#### 3.4.2. Trait anxiety and insomnia/daytime sleepiness in the caregiving situation.

Regarding insomnia, caregivers with high levels of trait anxiety had an average score of 13.14 (*SD* = ±4.14) on the ISI, whereas caregivers with low trait anxiety had a mean score of 9.13 points (*SD* = ±5.92). Palliative caregivers with high trait anxiety suffer more from insomnia (*T* = -2.58; *df* = 42; *p* = 0.013; Cohen’s d = -0,779; CI: LL = -1,390, UL = -0,161).

Regarding daytime sleepiness, participants with high levels of trait anxiety had an average score of 10.53 (*SD* = ±3.98) on the ESS, whereas participants with low trait anxiety had a mean score of 8.70 points (*SD* = ±4.39). No significant differences in daytime sleepiness were found between the groups (*T* = -1.44; *df* = 42; *p* = 0.157; Cohen’s d = -0,435; CI: LL = -1,031, UL = 0,166).

### 3.5. Psychosomatic complaints

Caregivers with high levels of trait anxiety had an average score of 22.71 (*SD* = ±13.03) on the GBB-24, whereas participants with less trait anxiety had a mean score of 22.41 points (*SD* = ±11.79). No significant difference was found between the two groups with regard to psychosomatic complaints (*T* = -0.08; *df* = 41; *p* = 0.936; Cohen’s d = -0,025; CI: LL = -0,622, UL = 0,574).

### 3.6. Resentments

#### 3.6.1. Resentments in the caregiving situation and stress.

A significant positive correlation was found between increased resentment (BVI) and stress levels in the caregiving situation (PSS-10; Pearson’s *r* = 0.431; *p* = 0.003). This shows that resentment is associated with high stress in the caring situation.

### 3.7. Trait anxiety and resentment in the caregiving situation

Caregivers with high levels of trait anxiety had an average score of 48.54 (*SD* = ± 6.12) on the BVI, whereas participants with low trait anxiety had a mean score of 39.56 points (*SD* = ± 6.44). Caregivers with high trait anxiety suffer from a higher level of resentment in the caregiving situation than people with low trait anxiety (*T* = -4.73; *df* = 42; *p* < 0.01; Cohen’s d = -1,427; CI: LL = -2,086, UL = -0,755).

### 3.8. Resilience and coping strategies

#### 3.8.1. Resilience and stress levels.

A bivariate Pearson correlation analysis was carried out, and a significant correlation was found between low resilience (BRSC) and high-stress levels in the caregiving situation (PSS-10; Pearson’s *r* = -0.398; *p* = 0.008), suggesting resilience may be associated with high stress levels in the caring situation.

#### 3.8.2. Resilience, coping strategies and trait anxiety.

Caregivers with high levels of trait anxiety had an average score of 13.6 (*SD* = ± 3.41) in terms of resilience and coping strategies (measured with the BRSC), whereas caregivers with low trait anxiety had a mean score of 14.6 points (*SD* = ± 3.13). No significant differences were found between the two groups (*T* = 1.00; *df* = 42; *p* = 0.320; Cohen’s d = 0,304; CI: LL = -0,293, UL = -0,897).

### 3.9. Emotion regulation

#### 3.9.1. Maladaptive Emotion Regulation Strategies and stress levels in the caregiving situation.

A correlation analysis revealed a significant positive correlation between maladaptive emotion regulation (FEEL-E) and stress levels in the caregiving situation (PSS-10; Pearson’s *r* = 0.419; *p* = 0.005). It can, therefore be assumed that the amount of maladaptive emotion regulation is associated with high stress levels in the caring situation.

#### 3.9.2. Maladaptive emotional regulation strategies and trait anxiety.

Caregivers with high levels of trait anxiety had an average score of 53.24 (*SD* = ± 12.99) in the FEEL-E for maladaptive strategies, whereas participants with low trait anxiety had a mean score of 44.40 points (*SD* = ±11.68; *T* = -2.38; *df* = 42; *p* = 0.022; Cohen’s d = -0,718; CI: LL = -1,325, UL = -0,103). This shows that people with high levels of trait anxiety also have a high level of maladaptive emotion regulation strategies.

#### 3.9.3. Adaptive emotional regulation strategies and trait anxiety.

In addition to the previously mentioned maladaptive strategies, adaptive strategies in emotion regulation were also examined.

Caregivers with high levels of trait anxiety had an average score of 39.90 (*SD* = ±10.09) for adaptive emotional regulation strategies, whereas participants with low trait anxiety had a mean score of 58.86 points (*SD* = ±10.64) for adaptive emotional regulation strategies (*T* = 5.99; *df* = 41; *p* < 0.01; Cohen’s d = 1,827; CI: LL = 1,103, UL = 2,535). This shows that family caregivers with low trait anxiety have an increased extent of adaptive strategies in the context of emotion regulation.

### 3.10. Work-related behavior and experience patterns

Caregivers with high levels of trait anxiety had a mean score of 4.55 (*SD* = ±2.77) concerning work-related behavior and experience patterns, whereas participants with low trait anxiety had a higher mean score of 5.45 points (*SD* = ±1.86). No significant difference was found between caregivers with high levels of trait anxiety and caregivers with low levels of trait anxiety with regard to work-related behavior and experience patterns (*T* = 0.90; *df* = 20; *p* = 0.377; Cohen’s d = 0,385; CI: LL = -0,464, UL = 1,225).

### 3.11. Social support

#### 3.11.1. Trait anxiety and affectionate support.

Caregivers with high levels of trait anxiety had in The MOS a mean score of 4.26 (*SD* = ±0.87) for receiving affectionate support, while caregivers with low trait anxiety had a mean score of 4.39 (*SD* = ±0.89). No significant difference was found between the two groups with regard to the level of affectionate support (*T* = 0.47; *df* = 40; *p* = 0.642; Cohen’s d = 0,145; CI: LL = -0,464, UL = 0,753).

#### 3.11.2. Trait anxiety and emotional support.

Caregivers with high levels of trait anxiety had in The MOS an average score of 4.2 (*SD* = ±0.77) for receiving emotional support, while those with low trait anxiety had a mean score of 4.04 points (*SD* =  ± 1.12). No significant differences in the levels of emotional support between the two groups were found (*T* = -0.53; *df* = 41; *p* = 0.599; Cohen’s d = -0,162; CI: LL = -0,762, UL = 0,439).

#### 3.11.3. Trait anxiety and physical support.

Caregivers with high levels of trait anxiety had in The MOS Social-Survey-Support a mean score of 4.05 (*SD* = ±1.13) for receiving physical support, while caregivers with low trait anxiety had a mean score of 4.22 (*SD* = ±0.99). No significant differences were found in the level of physical support between the two groups (*T* = 0.50; *df* = 40; *p* = 0.619; Cohen’s d = 0,156; CI: LL = -0,454, UL = 0,763).

#### 3.11.4. Trait anxiety and distraction.

Caregivers with high levels of trait anxiety had in The MOS Social-Survey-Support a mean score of 3.66 (*SD* = ±1.18) in the category of receiving distraction from everyday care by an external person, whereas those with low anxiety had a mean score of 3.74 points (*SD* = ±1.14) for receiving distraction. No significant differences were found in the level of distraction between the two groups (*T* = 0.25; *df* = 41; *p* = 0.803).

#### 3.11.5. Trait anxiety and positive social interaction.

Caregivers with high levels of trait anxiety had a mean score of 3.55 (*SD* = ±1.19) in the MOS Social-Survey-Support for receiving positive social interaction, while participants with low trait anxiety had a mean score of 3.91 (*SD* = ±0.99) for receiving positive social interaction. No significant difference in positive social interaction was found between the two groups (*T* = 1.09; *df* = 41; *p* = 0.283; Cohen’s d = 0,333; CI: LL = -0,273, UL = 0,934).

#### 3.11.6. Number of social supporters.

Caregivers with high levels of trait anxiety reported an average number of 3.76 supporters (*SD* = ±1.45), while caregivers with low trait anxiety reported an average number of 3.77 supporters (*SD* = ±1.45). No significant differences were found between the two groups concerning the number of supportive persons (*T* = 0.03; *df* = 41; *p* = 0.981; Cohen’s d = 0,77; CI: LL = -0,523, UL = 0,676).

#### 3.11.7. Total amount of support.

Caregivers with high levels of trait anxiety had a mean score of 4.16 (*SD* = ±0.90) in the MOS Social Survey Support scale total score of amount of support, whereas participants with low trait anxiety had a mean score of 3.96 points (*SD* = ±1.02) in the total score. No significant differences were found between the two groups with regard to the overall level of support (*T* = -0.67; *df* = 40; *p* = 0.506; Cohen’s d = -0,208; CI: LL = -0,816, UL = 0,403).

### 3.12. Summary of the findings

The results are summarized in Table 1.

**Table 1:** Effects of high trait anxiety in palliative family caregivers in comparison with palliative family caregivers with low trait anxiety.

High trait anxiety in palliative family caregivers		*p;* confidence interval (CI)
State Anxiety (STAI-State)	↑	*p* < 0.01 * ; CI: LL = -2,016, UL = -0,698
Stress (PSS-10)	↑	*p* < 0.01 * ; CI: LL = -2,390, UL = -1,002
Burnout: Depersonalization (MBI-HSS)	↑	*p* < 0.001 * ; CI: LL = -1,931, UL = -0,577
Burnout: emotional exhaustion (MBI-HSS)	↑	*p* < 0.001**; CI: LL = -1,773, UL = -0,444;
Insomnia (ISI)	↑	*p* = 0.013 * ; CI: LL = -1,390, UL = -0,161
Emotion regulation: maladaptive strategies (FEEL-E)	↑	*p* = 0.022 * ; CI: LL = -1,325, UL = -0,103
Resentments (BVI)	↑	*p* < 0.01 * ; CI: LL = -2,086, UL = -0,755
Health-related anxiety (W.I.)	↑	*p* = 0.007**; CI: LL = -1,476, UL = -0,237
Emotion regulation: adaptive strategies (FEEL-E)	↓	*p* < 0.01 * ; CI: LL = 1,103, UL = 2,535
Chronic stress perception at the beginning of the palliative family caregiving situation (TICS)	–	*p* = 0.16; CI: LL = -178, UL = -1,048
Burnout: personal performance (MBI-HSS)	–	*p* = 0.682; CI: LL = -0,488, UL = -0,746
Daytime sleepiness (ESS)	–	*p* = 0.157; CI: LL = -1,031, UL = 0,166
Physical complaints (GBB-24)	–	*p* = 0.936; CI: LL = -0,622, UL = 0,574
Resilience and coping strategies (BRCS)	–	*p* = 0.320; CI: LL = -0,293, UL = -0,897
Work-related behavior and experience patterns (AVEM-44)	–	*p* = 0.377; CI: LL = -0,464, UL = 1,225
Total amount and number of support (MOS-SSS)	–	*p* = 0.506; CI: LL = -0,816, UL = 0,403
Affectionate support (MOS-SSS)	–	*p* = 0.642; CI: LL = -0,464, UL = 0,753
Physical support (MOS-SSS)	–	*p* = 0.619; CI: LL = -0,454, UL = 0,763
Emotional support (MOS-SSS)	–	*p* = 0.599; CI: LL = -0,762, UL = 0,439
Positive social interaction (MOS-SSS)	–	*p* = 0.283; CI: LL = -0,273, UL = 0,934

Legend: ↑  increased in comparison with palliative family caregivers with low trait anxiety; ↓  less existent in comparison with palliative family caregivers with low trait anxiety; - equal to palliative family caregivers with low trait anxiety; LL =  lower limit, UL =  upper limit.

### 3.13. End-of-life support provided by the mobile palliative care team

#### 3.13.1. Changes in the stress load in the caregiving situation.

Study data showed that after six weeks, palliative caregivers with high levels of trait anxiety experienced an increase in chronic stress perception (TICS; *F* = 5.58; *df* = 1; *p* = 0.011). This increase in chronic stress can primarily be explained by the progression of the care recipient's illness.

However, the stress load in the caregiving situation decreases after the palliative care team's support starts. Thus, the stress levels in the caring situation (measured with the PSS-10) were assessed at the baseline and again six weeks later in caregivers with high trait anxiety and low trait anxiety. A significant time effect (*F*(time = 4.46; *df* = 1; *p* = 0.045; partial eta squared = 0,151) and a significant effect of group (*F*(group) = 28.10; *df* = 1; *p* < 0.01; partial eta squared = 0,529) were found. Family caregivers with high levels of trait anxiety suffered more from stress in the caregiving situation. The interaction time x group was not significant (*F*(time x group) = 0.04; *df* = 1; *p* = 0.836; partial eta squared = 0,002). Thus, after the start of the support of palliative care, the stress level decreased in all caregivers, regardless of the level of trait anxiety. In order to examine the impact of resilience on stress reduction an additional RM-ANCOVA was calculated that takes resilience into account as a co-variable. However, in this model resilience did not significantly predict stress reduction. (F_(time x BRCS)_ = 0.661, *df* = 1; *p* = 0.424).

Table 2 shows the stress load before and after the start of the support by the palliative care team.

**Table 2:** Stress load before and after the start of the support by the palliative care team.

	Stress load (PSS-10) before the start of the support provided by the palliative care team: (mean (M), *S.D*.)	Stress load (PSS-10) after the start of the support provided by the palliative care team: (mean (M), *S.D*.)
Trait anxiety ↑	*M*: 16.2 *SD*: ± 7.4	*M*: 21.1 *SD*: ± 10.8
Trait anxiety ↓	*M*: 19.5 *SD*: ± 7.9	*M*: 11.8 *SD*: ± 6.2

#### 3.13.2. Insomnia after receiving support from the mobile palliative team.

After a care period of six weeks, a correlation analysis was carried out, which revealed a significant positive correlation between comprehensive support (MOS) and insomnia (ISI; *r* = 0.402; *p* = 0.046). This means that comprehensive support by the mobile palliative team is associated with less insomnia in the palliative care situation.

## 4. Discussion

### 4.1. Main findings

Our study data revealed that family caregivers with high trait anxiety suffer from high levels of anxiety and stress during end-of-life palliative caregiving. It was also shown that caregivers with high trait anxiety have increased burnout symptoms, insomnia, resentment, and maladaptive strategies in the context of emotion regulation.

No significant differences were found between family caregivers with high and low trait anxiety in terms of their health-related fears, daytime sleepiness, physical complaints resilience, coping strategies, and support.

### 4.2. What does this study add?

#### 4.2.1. High levels of trait anxiety increase anxiety in the end-of-life palliative caregiving situation and health-related anxiety in palliative caregivers.

The results of our study show that palliative family caregivers with high trait anxiety suffer not only from high levels of anxiety in end-of-life palliative caregiving but also from high levels of health-related anxiety.

The majority of previous studies only reflect the frequent occurrence of feelings of anxiety in caregiving situations. It is implied that the daily physical and mental strain can trigger increased anxiety in affected persons [22]. In the work by Götze et al. [4] almost a third of caregivers have a high level of anxiety at the beginning of home care. Other studies report a prevalence of anxiety of 40 to 48% [23,24]. However, the existing literature does not take into account sufficiently the impact of pre-existing trait anxiety on anxiety in the current palliative care situation. A study by Hoellen et al. [25] describes that especially young caring men aged 18-35 show high levels of trait as well as state anxiety. In particular, anxiety levels were highest during appointments for surgery or examinations and tumor board meetings [25].

Family caregivers increasingly suffer from feelings of anxiety due to the progressive extent of the patient's symptoms, a frequent lack of knowledge about the diagnosis and prognosis, and an occasional lack of knowledge about nursing activities. It is also conceivable that many of those affected are burdened by the fact that their sick relative is still alive, possibly still in a satisfactory general condition, but the prognosis is poor and as a layperson you can neither influence nor assess the course of the disease and the risk of death. Regarding trait anxiety, Comte et al. showed that a dysfunction of the prefrontal control mechanisms plays a central role in feelings of anxiety. It is described that people with trait anxiety suffer from a distortion of their selective attention to negative stimuli [26].

#### 4.2.2. High levels of trait anxiety increase the experience of stress in the end-of-life palliative caregiving situation.

At the beginning of the end-of-life palliative care situation, family caregivers with high trait anxiety had a similarly perceived chronic stress load as caregivers with low trait anxiety. However, our results also showed that caregivers with high levels of trait anxiety suffer from increased stress in the caring situation and perceived chronic stress perception. A previous publication by Varona et al. [27] revealed that high scores in the State-Trait Anxiety Inventory of family caregivers are associated with a more intense perception of stressful situations. Another study confirmed that increased feelings of anxiety can triple the subjectively perceived caregiving burden and, therefore significantly influence the individual stress level [28]. In another study by Bidwell et al., also conducted with family caregivers with a focus on heart failure, it is described that the severity of the disease significantly influences the increased development of feelings of anxiety and the perception of stress and that this relationship can be regarded as directly proportional [29].

#### 4.2.3. High stress levels, trait anxiety, and burnout symptoms in the end-of-life palliative caregiving situation.

Our study data revealed a significant correlation between high stress levels and burnout symptoms such as depersonalization and emotional exhaustion. It is assumed that many people in the care sector suffer from burnout symptoms, which are often not recognized. Stressful workload is probably the most critical risk factor for burnout. If the level of workload increases, the affected person has less time to recover from stressful situations, which can lead to emotional exhaustion in the long term [30]. Other factors that can promote the development of burnout are a lack of control and a lack of recognition. An incongruence between personal values and the current situation can also lead to dissatisfaction and, in the longer term, to burnout symptoms [30]. It has also been described that depression and anxiety can often occur together with burnout [31]. Our study data also showed that especially family caregivers with high trait anxiety suffer from a high level of emotional exhaustion and depersonalization. However, regarding burnout-associated work-related behavior and experience patterns, no significant difference between family caregivers with high and low levels of trait anxiety could be found.

#### 4.2.4. Stress levels and insomnia in the end-of-life palliative caregiving situation.

The data of our study showed that there is a significant association between the occurrence of insomnia and high stress levels. One potential explanation might be that many family caregivers suffer from a longer latency period for sleep onset due to concerns and often double burden of the caring situation. Gao et al. [32] also add that numerous caregivers experience reduced sleep duration and quality, with their sleep often disturbed several times and for longer periods by the patient's nightly awakenings. The general perception of stress among family caregivers increases in the event of sleep disturbances [33] and may lead to stress-related disorders [34]. Thus, anxiety disorders and depression might be the result of sleep deprivation and vice versa. Neuroimaging studies using functional magnetic resonance imaging revealed an association between reduced connectivity of the left amygdala and the bilateral region of the anterior cingulate cortex, which subsequently leads to an increased risk of developing anxiety disorders in patients with insomnia [35]. Furthermore, the lack of sleep might have a negative impact on the physical and cognitive performance of family caregivers, so caregiving activities might be impaired in the long term. That recognition often triggers feelings of guilt and stress in family caregivers [32]. Insomnia related cognitive impairments might affect short-term memory and mental performance. Those affected have an increased risk of depression, cardiovascular disease and a higher mortality rate [36].

#### 4.2.5. Trait anxiety, insomnia and daytime sleepiness in the end-of-life palliative caregiving situation.

In this study, family caregivers with high levels of trait anxiety suffered more often from insomnia. One possible reason for the increased insomnia could be that family caregivers with increased trait anxiety are also physically and mentally more burdened with caring for their relatives, so the anxiety is further enhanced and, as a consequence, the quantity and quality of sleep deteriorates. Regarding daytime sleepiness, no significant differences between people with higher and lower trait anxiety were detected.

#### 4.2.6. Trait anxiety and physical complaints in the end-of-life palliative caregiving situation.

Regarding physical complaints, our study revealed that family caregivers with high levels of trait anxiety have a similar level of physical discomfort as those with low trait anxiety. In contrast, some previous studies have shown that family caregivers of palliative patients have more physical complaints than people who care for family members with curative illnesses and have a higher risk of hospitalization and increased medication use compared to non-bereaved people [4]. The grieving process often begins before the actual loss, which means that relatives are already confronted with symptoms of weakness, loss of appetite and reduced sleep quality during nursing care [37]. Many caregivers also suffer from chronic fatigue and weight loss [23].

#### 4.2.7. High levels of stress and trait anxiety increase feelings of resentment in the end-of-life palliative caregiving situation.

The data from our study showed an association between feelings of resentment and high stress levels in the palliative caregiving situation. A previous study by Lee et al. also reported an association between stress and the potential development of feelings of resentment. In addition, depression and educational background also appear to have an impact on the development of resentment [38]. In our study, family caregivers with high trait anxiety also suffered from a higher level of resentment than people with low trait anxiety. An explanation might be that family caregivers with high trait anxiety suffer from low self-esteem and blame themselves for not being able to prevent or cope with adverse events [38]. An earlier publication by Levy et al. [39] showed that approximately ¾  of caregivers experienced feelings of bitterness, anger, and sadness. Interpersonal slights often occur during home care situations and impact the subsequent grieving process.

#### 4.2.8. Resilience.

Our study data revealed an association between low resilience and high stress levels in the end-of-life palliative caregiving situation. This could be explained by the fact that an external stressor triggers a physiological stress response with activation of the hypothalamic-pituitary-adrenal axis, followed by a rapid recovery phase. Individuals with low resilience experience prolonged exposure to a stressful event and possible failure of recovery mechanisms, which can turn to chronic stress with persistent sequelae [40]. Lin et al. [41] describe that a greater degree of warmth and care from caregivers positively affects coping with stress and builds resilience. A high level of resilience can protect against stress-related symptoms and disorders and generally have a protective effect on mental health [42]. Thus, people with higher resilience can successfully adapt to the situation without developing persistent psychopathologies. Increased resilience, therefore, offers positive effects on one's mental health and the ageing process, which is slowed down as a result [40]. In contrast, people with low resilience tend to dysregulate their stress response in challenging situations, which can subsequently lead to psychiatric illness. An earlier publication of family caregivers reports that resilience training after a stressful event reduces the likelihood of anxiety, depressive symptoms and stress in family caregivers [28]. An increased level of trait anxiety is seen as one of the most important predictors of low resilience [42]. However, in our study, there was no significant difference in resilience between caregivers with high and low trait anxiety.

#### 4.2.9. High levels of stress and trait anxiety are associated with maladaptive emotion regulation.

Our data provide evidence that there is an association between maladaptive emotion regulation and high levels of stress and trait anxiety in everyday caregiving. One previous study by Yaroslavsky et al. [43] reports that the frequent use of maladaptive emotion regulation can lead to increased stress sensitization. The effects of everyday stress are subsequently exacerbated [43]. Earlier publications by Iavarone et al. [22] and Young et al. [44] describe that caregivers with maladaptive coping tendencies have a higher risk of burnout as well as prolonged depression and melancholy [22]. In addition, maladaptive emotion regulation may impair coping with feelings of anxiety and promote the avoidance of anxiety-inducing situations [45]. Disordered emotion regulation is therefore seen as a predictor rather than a consequence of future psychopathologies. All fMRI studies on impaired neuronal circuits of emotion regulation show that there are differences in activation and functional connectivity between different brain areas [44].

#### 4.2.10. Decreased stress load and insomnia after the start of end-of-life support from the mobile palliative team.

In this study before the onset of support provided by the mobile palliative team, caregivers with high trait anxiety did not differ in the extent of social support (total amount and number of support, affectionate support, physical support, emotional support, positive social interaction, distraction) from caregivers with low trait anxiety. Our study data showed an increase in chronic stress perception in caregivers with increased trait anxiety over the course of the study. This increase in stress in caregivers with high levels of trait anxiety might be explained by the progression of the patient's illness. However, after the beginning of the support provided by the mobile palliative team the stress levels of family caregivers in the palliative caring situation decreased over the course of six weeks in all caregivers, regardless of the extent of trait anxiety. This demonstrates the positive effect of the mobile palliative care team. Myoungsoon et al. [46] describe that receiving support can also have a positive effect on the emotional status of the respective person and that support can help to strengthen individual stress management skills in order to protect against negative psychological and physical consequences. There is also a significant correlation between comprehensive support and sleep quality. Thus, a decrease in insomnia was observed over the course of the study. An earlier study describes that with sufficient social support, the extent of depressive symptoms, feelings of anxiety and sleep disorders can be reduced. Social support is therefore seen as an important factor in preventing or counteracting psychological and somatic problems [47].

### 4.3. Clinical and practical implications for healthcare providers

Our study reveal multiple biopsychosocial burdens (including increased anxiety, stress, insomnia, burnout symptoms and resentment) among caregiving relatives with high trait anxiety. This group of relatives in particular should therefore benefit from early psychosocial support from trained professionals and palliative care teams might offer an early screening in this context to prevent potential mental health comorbidities.

### 4.4. Limitations

The study is based on the self-reports of the family caregivers which is why social desirability cannot be ruled out in the evaluation and response. The study results should be replicated in a study with a higher sample size and future studies should also enroll individuals with a history of mental illness.

## 5. Conclusions

Palliative caregiving might lead to increased anxiety, stress load, burnout symptoms, insomnia, physical complaints and feelings of resentment. Especially family caregivers with high levels of pre-existing trait anxiety suffer in the palliative care situation more from anxiety, stress, burnout symptoms, increased own health-related anxiety, insomnia and feelings of resentment within the caregiving situation. Regarding emotional regulation, they also apply from maladaptive strategies. Therefore, caregiving relatives with pre-existing high trait anxiety should be identified at an early stage in order to offer them comprehensive biopsychosocial support. They might especially benefit from holistic, palliative medical care. In addition to alleviating and controlling symptoms in patients themselves, the mobile palliative care team also does valuable work for family caregivers like supporting them in medical, nursing and socio-psychological matters during the care period. It would be conceivable to offer family caregivers with high levels of pre-existing trait anxiety professional, accompanying support in advance in order to minimize serious mental health symptoms and to provide adequate help at an early stage.

## Supporting information

S1 Data Data Record .
(SAV)

S2 Data Sociodemographic Data. (XLSX)

S3 Data Statistical Analysis. (PDF)
