# Correction: Biopsychosocial impact of high levels of trait anxiety on family caregivers in the end-of-life palliative care setting

**DOI:** 10.1371/journal.pone.0322896

**Published:** 2025-04-22

**Authors:** Anastasia Maria Grumeth, Hans-Bernd Rothenhäusler, Sabrina Mörkl, Jolana Wagner-Skacel, Elisabeth Sciri, Andreas Baranyi

There are errors in the author affiliations. The correct affiliations are as follows:

Anastasia Maria Grumeth^1^, Hans-Bernd Rothenhäusler^1^, Sabrina Mörkl^2^, Jolana Wagner-Skacel^2^, Elisabeth Sciri^3^, Andreas Baranyi^1^

1 Department of Psychiatry, Psychosomatics and Psychotherapeutic Medicine, Division of Psychiatry and Psychotherapeutic Medicine, Medical University of Graz, Graz, Austria, 2 Department of Psychiatry, Psychosomatics and Psychotherapeutic Medicine, Division of Medical Psychology, Psychosomatics and Psychotherapeutic Medicine, Medical University of Graz, Graz, Austria, 3 Department of Internal Medicine, University Palliative Care Facility, Medical University of Graz, Graz, Austria.
