# Supplementary material for: Biopsychosocial impact of high levels of trait anxiety on family caregivers in the end-of-life palliative care setting
Source: PLoS One. 2025 Mar 18;20(3):e0307349. doi: 10.1371/journal.pone.0307349 (PMC11918354; doi:10.1371/journal.pone.0307349)
Supplement: S3 Data — (PDF) [file pone.0307349.s003.pdf]

```
GET
  FILE='C:\Users\baranyia\AppData\Local\Microsoft\Windows\INetCache\Content.Outlook\WF
PFBIOG\Dateneingabe DA (1)_1 (003).sav'.
DATASET NAME DataSet1 WINDOW=FRONT.
GET
  FILE='C:\Users\baranyia\AppData\Local\Microsoft\Windows\INetCache\Content.Outlook\WF
PFBIOG\Dateneingabe DA (1)_1 (005).sav'.
DATASET NAME DataSet2 WINDOW=FRONT.
T-TEST GROUPS=STAITraitMedianaufteilungErsterZP(1 2)
  /MISSING=ANALYSIS
  /VARIABLES=Alter
  /ES DISPLAY(TRUE)
  /CRITERIA=CI(.95).
```

t-Test

```
[DataSet2] C:\Users\baranyia\AppData\Local\Microsoft\Windows\INetCache\Content.Outlook
\WFPFBIOG\Dateneingabe DA (1)_1 (005).sav
```

Gruppenstatistiken

	STAITraitMedianaufteilung ErsterZP	N	Mittelwert	Std.-Abweichung	Standardfehler des Mittelwertes
Alter	1bis40	23	59,1304	10,95156	2,28356
	ab41	21	57,3810	15,47410	3,37672

Test bei unabhängigen Stichproben

		Levene-Test der Varianzgleichheit		t-Test für die Mittelwertgleichheit	
		F	Sig.	T	df
Alter	Varianzen sind gleich	4,291	,045	,436	42
	Varianzen sind nicht gleich			,429	35,690

Test bei unabhängigen Stichproben

		t-Test für die Mittelwertgleichheit			
					95% Konfidenzinterv..
		Sig. (2-seitig)	Mittlere Differenz	Differenz für Standardfehler	Unterer Wert
Alter	Varianzen sind gleich	,665	1,74948	4,01376	-6,35061
	Varianzen sind nicht gleich	,670	1,74948	4,07638	-6,52030

Test bei unabhängigen Stichproben

		t-Test für die Mittelwertgleichh.. 95% Konfidenzinterval.. Oberer Wert
Alter	Varianzen sind gleich	9,84958
	Varianzen sind nicht gleich	10,01926

Effektgrößen bei unabhängigen Stichproben

		Standardisierer[a]	Punktschätzung	95% Konfidenzintervall Unterer Wert	Oberer Wert
Alter	Cohen's d	13,29837	,132	-,461	,723
	Hedges' Korrektur	13,54188	,129	-,453	,710
	Glass' Delta	15,47410	,113	-,481	,704

a. Der bei der Schätzung der Effektgrößen verwendete Nenner.
Cohen's d verwendet die zusammengefasste Standardabweichung.
Hedges' Korrektur verwendet die zusammengefasste Standardabweichung und einen Korrekturfaktor.
Glass' Delta verwendet die Standardabweichung einer Stichprobe von der Kontrollgruppe.

```
T-TEST GROUPS=STAITraitMedianaufteilungErsterZP(1 2)
  /MISSING=ANALYSIS
  /VARIABLES=Unterstützungsanzahl
  /ES DISPLAY(TRUE)
  /CRITERIA=CI(.95).
```

t-Test

Gruppenstatistiken

	STAITraitMedianaufteilung ErsterZP	N	Mittelwert	Std.-Abweichung
Unterstützungsanzahl	1bis40	22	3,7727	1,44525
	ab41	21	3,7619	1,44585

Gruppenstatistiken

	STAITraitMedianaufteilung ErsterZP	Standardfehler des Mittelwertes
Unterstützungsanzahl	1bis40	,30813
	ab41	,31551

Test bei unabhängigen Stichproben

		Levene-Test der Varianzgleichheit		t-Test für die ...
		F	Sig.	T
Unterstützungsanzahl	Varianzen sind gleich	,000	,994	,025
	Varianzen sind nicht gleich			,025

Test bei unabhängigen Stichproben

		t-Test für die Mittelwertgleichheit		
		df	Sig. (2-seitig)	Mittlere Differenz
Unterstützungsanzahl	Varianzen sind gleich	41	,981	,01082
	Varianzen sind nicht gleich	40,905	,981	,01082

Test bei unabhängigen Stichproben

		t-Test für die Mittelwertgleichheit		
		Differenz für Standardfehler	95% Konfidenzintervall der Differenz	
			Unterer Wert	Oberer Wert
Unterstützungsanzahl	Varianzen sind gleich	,44101	-,87981	,90145
	Varianzen sind nicht gleich	,44101	-,87988	,90152

Effektgrößen bei unabhängigen Stichproben

		Standardisierer[a]	Punktschätzung	95% ... Unterer Wert
Unterstützungsanzahl	Cohen's d	1,44554	,007	-,591
	Hedges' Korrektur	1,47267	,007	-,580
	Glass' Delta	1,44585	,007	-,591

Effektgrößen bei unabhängigen Stichproben

		95% ... Oberer Wert
Unterstützungsanzahl	Cohen's d	,605
	Hedges' Korrektur	,594
	Glass' Delta	,605

a. Der bei der Schätzung der Effektgrößen verwendete Nenner.
Cohen's d verwendet die zusammengefasste Standardabweichung.
Hedges' Korrektur verwendet die zusammengefasste Standardabweichung und einen Korrekturfaktor.
Glass' Delta verwendet die Standardabweichung einer Stichprobe von der Kontrollgruppe.

```
T-TEST GROUPS=STAITraitMedianaufteilungErsterZP(1 2)
  /MISSING=ANALYSIS
  /VARIABLES=STAIStateersterZP
  /ES DISPLAY(TRUE)
  /CRITERIA=CI(.95).
```

t-Test

Gruppenstatistiken

	STAITraitMedianaufteilung ErsterZP	N	Mittelwert	Std.-Abweichung
STAIStateersterZP	1bis40	23	42,7826	10,40960
	ab41	21	56,4286	9,54239

Gruppenstatistiken

	STAITraitMedianaufteilung ErsterZP	Standardfehler des Mittelwertes
STAIStateersterZP	1bis40	2,17055
	ab41	2,08232

Test bei unabhängigen Stichproben

		Levene-Test der Varianzgleichheit		t-Test für die ...
		F	Sig.	T
STAIStateersterZP	Varianzen sind gleich	,152	,699	-4,518
	Varianzen sind nicht gleich			-4,537

Test bei unabhängigen Stichproben

		t-Test für die Mittelwertgleichheit		
		df	Sig. (2-seitig)	Mittlere Differenz
STAIStateersterZP	Varianzen sind gleich	42	,000	-13,64596
	Varianzen sind nicht gleich	41,998	,000	-13,64596

Test bei unabhängigen Stichproben

		t-Test für die Mittelwertgleichheit		
			95% Konfidenzintervall der Differenz	
		Differenz für Standardfehler	Unterer Wert	Oberer Wert
STAIStateersterZP	Varianzen sind gleich	3,02005	-19,74067	-7,55126
	Varianzen sind nicht gleich	3,00788	-19,71612	-7,57581

Effektgrößen bei unabhängigen Stichproben

		Standardisierer[a]	Punktschätzung	95% ...
				Unterer Wert
STAIStateersterZP	Cohen's d	10,00602	-1,364	-2,016
	Hedges' Korrektur	10,18924	-1,339	-1,980
	Glass' Delta	9,54239	-1,430	-2,156

Effektgrößen bei unabhängigen Stichproben

		95% ...
		Oberer Wert
STAIStateersterZP	Cohen's d	-,698
	Hedges' Korrektur	-,685
	Glass' Delta	-,681

a. Der bei der Schätzung der Effektgrößen verwendete Nenner.
Cohen's d verwendet die zusammengefasste Standardabweichung.
Hedges' Korrektur verwendet die zusammengefasste Standardabweichung und einen Korrekturfaktor.
Glass' Delta verwendet die Standardabweichung einer Stichprobe von der Kontrollgruppe.

```
T-TEST GROUPS=STAITraitMedianaufteilungErsterZP(1 2)
  /MISSING=ANALYSIS
  /VARIABLES=STAIStatezweiterZP
  /ES DISPLAY(TRUE)
  /CRITERIA=CI(.95).
```

t-Test

Gruppenstatistiken

	STAITraitMedianaufteilung ErsterZP	N	Mittelwert	Std.-Abweichung
STAIStatezweiterZP	1bis40	13	39,3846	11,68716
	ab41	14	55,3571	12,86383

Gruppenstatistiken

	STAITraitMedianaufteilung ErsterZP	Standardfehler des Mittelwertes
STAIStatezweiterZP	1bis40	3,24144
	ab41	3,43800

Test bei unabhängigen Stichproben

		Levene-Test der Varianzgleichheit		t-Test für die ...
		F	Sig.	T
STAIStatezweiterZP	Varianzen sind gleich	,414	,526	-3,368
	Varianzen sind nicht gleich			-3,380

Test bei unabhängigen Stichproben

		t-Test für die Mittelwertgleichheit		
		df	Sig. (2-seitig)	Mittlere Differenz
STAIStatezweiterZP	Varianzen sind gleich	25	,002	-15,97253
	Varianzen sind nicht gleich	24,991	,002	-15,97253

Test bei unabhängigen Stichproben

		t-Test für die Mittelwertgleichheit		
			95% Konfidenzintervall der Differenz	
		Differenz für Standardfehler	Unterer Wert	Oberer Wert
STAIStatezweiterZP	Varianzen sind gleich	4,74256	-25,74000	-6,20505
	Varianzen sind nicht gleich	4,72512	-25,70427	-6,24079

Effektgrößen bei unabhängigen Stichproben

		Standardisierer[a]	Punktschätzung	95% ... Unterer Wert
STAIStatezweiterZP	Cohen's d	12,31307	-1,297	-2,122
	Hedges' Korrektur	12,69852	-1,258	-2,058
	Glass' Delta	12,86383	-1,242	-2,115

Effektgrößen bei unabhängigen Stichproben

		95% ... Oberer Wert
STAIStatezweiterZP	Cohen's d	-,451
	Hedges' Korrektur	-,438
	Glass' Delta	-,334

a. Der bei der Schätzung der Effektgrößen verwendete Nenner.
Cohen's d verwendet die zusammengefasste Standardabweichung.
Hedges' Korrektur verwendet die zusammengefasste Standardabweichung und einen Korrekturfaktor.
Glass' Delta verwendet die Standardabweichung einer Stichprobe von der Kontrollgruppe.

```
T-TEST GROUPS=STAITraitMedianaufteilungErsterZP(1 2)
  /MISSING=ANALYSIS
  /VARIABLES=FEELEadaptiveStrategienersterZP
  /ES DISPLAY(TRUE)
  /CRITERIA=CI(.95).
```

t-Test

Gruppenstatistiken

	STAITraitMedianaufteilung ErsterZP	N	Mittelwert	Std.-Abweichung
FEELEadaptiveStrategienersterZP	1bis40	22	58,8636	10,64266
	ab41	21	39,9048	10,08913

Gruppenstatistiken

	STAITraitMedianaufteilung ErsterZP	Standardfehler des Mittelwertes
FEELEadaptiveStrategienersterZP	1bis40	2,26902
	ab41	2,20163

Test bei unabhängigen Stichproben

		Levene-Test der Varianzgleichheit		t-Test für die ...
		F	Sig.	T
FEELEadaptiveStrategienersterZP	Varianzen sind gleich	,112	,739	5,989
	Varianzen sind nicht gleich			5,997

Test bei unabhängigen Stichproben

		t-Test für die Mittelwertgleichheit		
		df	Sig. (2-seitig)	Mittlere Differenz
FEELEadaptiveStrategienersterZP	Varianzen sind gleich	41	,000	18,95887
	Varianzen sind nicht gleich	40,999	,000	18,95887

Test bei unabhängigen Stichproben

		t-Test für die Mittelwertgleichheit	
			95% Konfidenzinterv..
		Differenz für Standardfehler	Unterer Wert
FEELEadaptiveStrategienersterZP	Varianzen sind gleich	3,16561	12,56580
	Varianzen sind nicht gleich	3,16159	12,57391

Test bei unabhängigen Stichproben

		t-Test für die Mittelwertgleichh..
		95% Konfidenzinterval..
		Oberer Wert
FEELEadaptiveStrategienersterZP	Varianzen sind gleich	25,35195
	Varianzen sind nicht gleich	25,34383

Effektgrößen bei unabhängigen Stichproben

		Standardisierer[a]	Punktschätzung	95% ... Unterer Wert
FEELEadaptiveStrategienersterZP	Cohen's d	10,37633	1,827	1,103
	Hedges' Korrektur	10,57109	1,793	1,083
	Glass' Delta	10,08913	1,879	1,035

Effektgrößen bei unabhängigen Stichproben

		95% ... Oberer Wert
FEELEadaptiveStrategienersterZP	Cohen's d	2,535
	Hedges' Korrektur	2,489
	Glass' Delta	2,699

a. Der bei der Schätzung der Effektgrößen verwendete Nenner.
Cohen's d verwendet die zusammengefasste Standardabweichung.
Hedges' Korrektur verwendet die zusammengefasste Standardabweichung und einen Korrekturfaktor.
Glass' Delta verwendet die Standardabweichung einer Stichprobe von der Kontrollgruppe.

```
T-TEST GROUPS=STAITraitMedianaufteilungErsterZP(1 2)
  /MISSING=ANALYSIS
  /VARIABLES=FEELEmaladaptiveStrategienersterZP
  /ES DISPLAY(TRUE)
  /CRITERIA=CI(.95).
```

t-Test

Gruppenstatistiken

	STAITraitMedianaufteilungErsterZP	N	Mittelwert	Std.-Abweichung
FEELEmaladaptiveStrategienersterZP	1bis40	23	44,3913	11,68036
	ab41	21	53,2381	12,99579

Gruppenstatistiken

	STAITraitMedianaufteilungErsterZP	Standardfehler des Mittelwertes
FEELEmaladaptiveStrategienersterZP	1bis40	2,43552
	ab41	2,83591

Test bei unabhängigen Stichproben

		Levene-Test der Varianzgleichheit		t-Test für die ...
		F	Sig.	T
FEELEmaladaptiveStrategienersterZP	Varianzen sind gleich	,016	,899	-2,378
	Varianzen sind nicht gleich			-2,367

Test bei unabhängigen Stichproben

		t-Test für die Mittelwertgleichheit		
		df	Sig. (2-seitig)	Mittlere Differenz
FEELEmaladaptiveStrategienersterZP	Varianzen sind gleich	42	,022	-8,84679
	Varianzen sind nicht gleich	40,402	,023	-8,84679

Test bei unabhängigen Stichproben

		t-Test für die Mittelwertgleichheit	
			95% Konfidenzinterv..
		Differenz für Standardfehler	Unterer Wert
FEELEmaladaptiveStrategienersterZP	Varianzen sind gleich	3,71975	-16,35356
	Varianzen sind nicht gleich	3,73821	-16,39964

Test bei unabhängigen Stichproben

		t-Test für die Mittelwertgleichh..
		95% Konfidenzinterval..
		Oberer Wert
FEELEmaladaptiveStrategienersterZP	Varianzen sind gleich	-1,34002
	Varianzen sind nicht gleich	-1,29394

Effektgrößen bei unabhängigen Stichproben

		Standardisierer[a]	Punktschätzung	95% ... Unterer Wert
FEELEmaladaptiveStrategienersterZP	Cohen's d	12,32428	-,718	-1,325
	Hedges' Korrektur	12,54995	-,705	-1,301
	Glass' Delta	12,99579	-,681	-1,301

Effektgrößen bei unabhängigen Stichproben

		95% ... Oberer Wert
FEELEmaladaptiveStrategienersterZP	Cohen's d	-,103
	Hedges' Korrektur	-,101
	Glass' Delta	-,046

a. Der bei der Schätzung der Effektgrößen verwendete Nenner.
Cohen's d verwendet die zusammengefasste Standardabweichung.
Hedges' Korrektur verwendet die zusammengefasste Standardabweichung und einen Korrekturfaktor.
Glass' Delta verwendet die Standardabweichung einer Stichprobe von der Kontrollgruppe.

```
T-TEST GROUPS=STAITraitMedianaufteilungErsterZP(1 2)
  /MISSING=ANALYSIS
  /VARIABLES=SSSIemotionalsupportersterZP
  /ES DISPLAY(TRUE)
  /CRITERIA=CI(.95).
```

t-Test

Gruppenstatistiken

	STAITraitMedianaufteilung ErsterZP	N	Mittelwert	Std.-Abweichung
SSSIemotionalsupportersterZP	1bis40	23	4,0435	1,10693
	ab41	20	4,2000	,76777

Gruppenstatistiken

	STAITraitMedianaufteilung ErsterZP	Standardfehler des Mittelwertes
SSSIemotionalsupportersterZP	1bis40	,23081
	ab41	,17168

Test bei unabhängigen Stichproben

		Levene-Test der Varianzgleichheit		t-Test für die ...
		F	Sig.	T
SSSIemotionalsupporterste rZP	Varianzen sind gleich	1,148	,290	-,531
	Varianzen sind nicht gleich			-,544

Test bei unabhängigen Stichproben

		t-Test für die Mittelwertgleichheit		
		df	Sig. (2-seitig)	Mittlere Differenz
SSSIemotionalsupporterste rZP	Varianzen sind gleich	41	,599	-,15652
	Varianzen sind nicht gleich	39,188	,589	-,15652

Test bei unabhängigen Stichproben

		t-Test für die Mittelwertgleichheit	
			95% Konfidenzinterv..
		Differenz für Standardfehler	Unterer Wert
SSSIemotionalsupporterste rZP	Varianzen sind gleich	,29495	-,75218
	Varianzen sind nicht gleich	,28766	-,73828

Test bei unabhängigen Stichproben

		t-Test für die Mittelwertgleichh..
		95% Konfidenzinterval..
		Oberer Wert
SSSIemotionalsupporterste rZP	Varianzen sind gleich	,43914
	Varianzen sind nicht gleich	,42523

Effektgrößen bei unabhängigen Stichproben

		Standardisierer[a]	Punktschätzung	95% ... Unterer Wert
SSSIemotionalsupporterste rZP	Cohen's d	,96470	-,162	-,762
	Hedges' Korrektur	,98281	-,159	-,748
	Glass' Delta	,76777	-,204	-,804

Effektgrößen bei unabhängigen Stichproben

		95% ... Oberer Wert
SSSIemotionalsupporterste rZP	Cohen's d	,439
	Hedges' Korrektur	,431
	Glass' Delta	,401

a. Der bei der Schätzung der Effektgrößen verwendete Nenner.
Cohen's d verwendet die zusammengefasste Standardabweichung.
Hedges' Korrektur verwendet die zusammengefasste Standardabweichung und einen Korrekturfaktor.
Glass' Delta verwendet die Standardabweichung einer Stichprobe von der Kontrollgruppe.

```
T-TEST GROUPS=STAITraitMedianaufteilungErsterZP(1 2)
  /MISSING=ANALYSIS
  /VARIABLES=SSSItangiblesupportersterZP
  /ES DISPLAY(TRUE)
  /CRITERIA=CI(.95).
```

t-Test

Gruppenstatistiken

	STAITraitMedianaufteilung ErsterZP	N	Mittelwert	Std.-Abweichung
SSSItangiblesupportersterZP	1bis40	23	4,2174	,99802
	ab41	19	4,0526	1,12909

Gruppenstatistiken

	STAITraitMedianaufteilung ErsterZP	Standardfehler des Mittelwertes
SSSItangiblesupportersterZP	1bis40	,20810
	ab41	,25903

Test bei unabhängigen Stichproben

		Levene-Test der Varianzgleichheit		t-Test für die ...
		F	Sig.	T
SSSItangiblesupportersterZP	Varianzen sind gleich	,008	,927	,502
	Varianzen sind nicht gleich			,496

Test bei unabhängigen Stichproben

		t-Test für die Mittelwertgleichheit		
		df	Sig. (2-seitig)	Mittlere Differenz
SSSItangiblesupportersterZP	Varianzen sind gleich	40	,619	,16476
	Varianzen sind nicht gleich	36,346	,623	,16476

Test bei unabhängigen Stichproben

		t-Test für die Mittelwertgleichheit	
			95% Konfidenzinterv..
		Differenz für Standardfehler	Unterer Wert
SSSItangiblesupportersterZP	Varianzen sind gleich	,32831	-,49878
	Varianzen sind nicht gleich	,33227	-,50889

Test bei unabhängigen Stichproben

		t-Test für die Mittelwertgleichh..
		95% Konfidenzinterval..
		Oberer Wert
SSSItangiblesupportersterZP	Varianzen sind gleich	,82830
	Varianzen sind nicht gleich	,83841

Effektgrößen bei unabhängigen Stichproben

		Standardisierer[a]	Punktschätzung	95% ... Unterer Wert
SSSItangiblesupportersterZP	Cohen's d	1,05901	,156	-,454
	Hedges' Korrektur	1,07940	,153	-,445
	Glass' Delta	1,12909	,146	-,466

Effektgrößen bei unabhängigen Stichproben

		95% ... Oberer Wert
SSSItangiblesupportersterZP	Cohen's d	,763
	Hedges' Korrektur	,749
	Glass' Delta	,753

a. Der bei der Schätzung der Effektgrößen verwendete Nenner.
Cohen's d verwendet die zusammengefasste Standardabweichung.
Hedges' Korrektur verwendet die zusammengefasste Standardabweichung und einen Korrekturfaktor.
Glass' Delta verwendet die Standardabweichung einer Stichprobe von der Kontrollgruppe.

```
T-TEST GROUPS=STAITraitMedianaufteilungErsterZP(1 2)
  /MISSING=ANALYSIS
  /VARIABLES=SSSIaffectionatesupportersterZP
  /ES DISPLAY(TRUE)
  /CRITERIA=CI(.95).
```

t-Test

Gruppenstatistiken

	STAITraitMedianaufteilung ErsterZP	N	Mittelwert	Std.-Abweichung
SSSIaffectionatesupportersterZP	1bis40	23	4,3913	,89133
	ab41	19	4,2632	,87191

Gruppenstatistiken

	STAITraitMedianaufteilung ErsterZP	Standardfehler des Mittelwertes
SSSIaffectionatesupportersterZP	1bis40	,18585
	ab41	,20003

Test bei unabhängigen Stichproben

		Levene-Test der Varianzgleichheit		t-Test für die ...
		F	Sig.	T
SSSIaffectionatesupporters terZP	Varianzen sind gleich	,083	,775	,468
	Varianzen sind nicht gleich			,469

Test bei unabhängigen Stichproben

		t-Test für die Mittelwertgleichheit		
		df	Sig. (2-seitig)	Mittlere Differenz
SSSIaffectionatesupporters terZP	Varianzen sind gleich	40	,642	,12815
	Varianzen sind nicht gleich	38,821	,641	,12815

Test bei unabhängigen Stichproben

		t-Test für die Mittelwertgleichheit	
			95% Konfidenzinterv..
		Differenz für Standardfehler	Unterer Wert
SSSIaffectionatesupporters terZP	Varianzen sind gleich	,27363	-,42489
	Varianzen sind nicht gleich	,27305	-,42422

Test bei unabhängigen Stichproben

		t-Test für die Mittelwertgleichh..
		95% Konfidenzinterval..
		Oberer Wert
SSSIaffectionatesupporters terZP	Varianzen sind gleich	,68118
	Varianzen sind nicht gleich	,68052

Effektgrößen bei unabhängigen Stichproben

		Standardisierer[a]	Punktschätzung	95% ... Unterer Wert
SSSIaffectionatesupportersterZP	Cohen's d	,88264	,145	-,464
	Hedges' Korrektur	,89964	,142	-,455
	Glass' Delta	,87191	,147	-,465

Effektgrößen bei unabhängigen Stichproben

		95% ... Oberer Wert
SSSIaffectionatesupportersterZP	Cohen's d	,753
	Hedges' Korrektur	,739
	Glass' Delta	,754

a. Der bei der Schätzung der Effektgrößen verwendete Nenner.
Cohen's d verwendet die zusammengefasste Standardabweichung.
Hedges' Korrektur verwendet die zusammengefasste Standardabweichung und einen Korrekturfaktor.
Glass' Delta verwendet die Standardabweichung einer Stichprobe von der Kontrollgruppe.

```
T-TEST GROUPS=STAITraitMedianaufteilungErsterZP(1 2)
  /MISSING=ANALYSIS
  /VARIABLES=SSSIpositivesocialinteractionersterZP
  /ES DISPLAY(TRUE)
  /CRITERIA=CI(.95).
```

t-Test

Gruppenstatistiken

	STAITraitMedianaufteilung ErsterZP	N	Mittelwert	Std.-Abweichung
SSSIpositivesocialinteractionersterZP	1bis40	23	3,9130	,99604
	ab41	20	3,5500	1,19097

Gruppenstatistiken

	STAITraitMedianaufteilung ErsterZP	Standardfehler des Mittelwertes
SSSIpositivesocialinteractionersterZP	1bis40	,20769
	ab41	,26631

Test bei unabhängigen Stichproben

		Levene-Test der Varianzgleichheit		t-Test für die ...
		F	Sig.	T
SSSIpositivesocialinteractionersterZP	Varianzen sind gleich	1,089	,303	1,089
	Varianzen sind nicht gleich			1,075

Test bei unabhängigen Stichproben

		t-Test für die Mittelwertgleichheit		
		df	Sig. (2-seitig)	Mittlere Differenz
SSSIpositivesocialinteractionersterZP	Varianzen sind gleich	41	,283	,36304
	Varianzen sind nicht gleich	37,242	,289	,36304

Test bei unabhängigen Stichproben

		t-Test für die Mittelwertgleichheit	
			95% Konfidenzinterv..
		Differenz für Standardfehler	Unterer Wert
SSSIpositivesocialinteractionersterZP	Varianzen sind gleich	,33348	-,31043
	Varianzen sind nicht gleich	,33772	-,32109

Test bei unabhängigen Stichproben

		t-Test für die Mittelwertgleichh..
		95% Konfidenzinterval..
		Oberer Wert
SSSIpositivesocialinteractionersterZP	Varianzen sind gleich	1,03652
	Varianzen sind nicht gleich	1,04718

Effektgrößen bei unabhängigen Stichproben

		Standardisierer[a]	Punktschätzung	95% ... Unterer Wert
SSSIpositivesocialinteractionersterZP	Cohen's d	1,09072	,333	-,273
	Hedges' Korrektur	1,11119	,327	-,268
	Glass' Delta	1,19097	,305	-,306

Effektgrößen bei unabhängigen Stichproben

		95% ... Oberer Wert
SSSIpositivesocialinteractionersterZP	Cohen's d	,934
	Hedges' Korrektur	,917
	Glass' Delta	,908

a. Der bei der Schätzung der Effektgrößen verwendete Nenner.
Cohen's d verwendet die zusammengefasste Standardabweichung.
Hedges' Korrektur verwendet die zusammengefasste Standardabweichung und einen Korrekturfaktor.
Glass' Delta verwendet die Standardabweichung einer Stichprobe von der Kontrollgruppe.

```
T-TEST GROUPS=STAITraitMedianaufteilungErsterZP(1 2)
  /MISSING=ANALYSIS
  /VARIABLES=SSSIadditionalitemersterZP
  /ES DISPLAY(TRUE)
  /CRITERIA=CI(.95).
```

t-Test

Gruppenstatistiken

	STAITraitMedianaufteilung ErsterZP	N	Mittelwert	Std.-Abweichung
SSSIadditionalitemersterZP	1bis40	23	3,7391	1,13688
	ab41	20	3,6500	1,18210

Gruppenstatistiken

	STAITraitMedianaufteilung ErsterZP	Standardfehler des Mittelwertes
SSSIadditionalitemersterZP	1bis40	,23706
	ab41	,26433

Test bei unabhängigen Stichproben

		Levene-Test der Varianzgleichheit		t-Test für die ...
		F	Sig.	T
SSSIadditionalitemersterZP	Varianzen sind gleich	,509	,480	,252
	Varianzen sind nicht gleich			,251

Test bei unabhängigen Stichproben

		t-Test für die Mittelwertgleichheit		
		df	Sig. (2-seitig)	Mittlere Differenz
SSSIadditionalitemersterZP	Varianzen sind gleich	41	,803	,08913
	Varianzen sind nicht gleich	39,684	,803	,08913

Test bei unabhängigen Stichproben

		t-Test für die Mittelwertgleichheit	
			95% Konfidenzinterv..
		Differenz für Standardfehler	Unterer Wert
SSSIadditionalitemersterZP	Varianzen sind gleich	,35407	-,62592
	Varianzen sind nicht gleich	,35505	-,62864

Test bei unabhängigen Stichproben

		t-Test für die Mittelwertgleichh..
		95% Konfidenzinterval..
		Oberer Wert
SSSIadditionalitemersterZP	Varianzen sind gleich	,80418
	Varianzen sind nicht gleich	,80690

Effektgrößen bei unabhängigen Stichproben

		Standardisierer[a]	Punktschätzung	95% ... Unterer Wert
SSSladditionalitemersterZP	Cohen's d	1,15806	,077	-,523
	Hedges' Korrektur	1,17979	,076	-,513
	Glass' Delta	1,18210	,075	-,525

Effektgrößen bei unabhängigen Stichproben

		95% ... Oberer Wert
SSSladditionalitemersterZP	Cohen's d	,676
	Hedges' Korrektur	,664
	Glass' Delta	,674

a. Der bei der Schätzung der Effektgrößen verwendete Nenner.
Cohen's d verwendet die zusammengefasste Standardabweichung.
Hedges' Korrektur verwendet die zusammengefasste Standardabweichung und einen Korrekturfaktor.
Glass' Delta verwendet die Standardabweichung einer Stichprobe von der Kontrollgruppe.

```
T-TEST GROUPS=STAITraitMedianaufteilungErsterZP(1 2)
  /MISSING=ANALYSIS
  /VARIABLES=SSSIoverallsupportindexersterZP
  /ES DISPLAY(TRUE)
  /CRITERIA=CI(.95).
```

t-Test

Gruppenstatistiken

	STAITraitMedianaufteilung ErsterZP	N	Mittelwert	Std.-Abweichung
SSSloverallsupportindexersterZP	1bis40	23	3,9565	1,02151
	ab41	19	4,1579	,89834

Gruppenstatistiken

	STAITraitMedianaufteilung ErsterZP	Standardfehler des Mittelwertes
SSSloverallsupportindexersterZP	1bis40	,21300
	ab41	,20609

Test bei unabhängigen Stichproben

		Levene-Test der Varianzgleichheit		t-Test für die ...
		F	Sig.	T
SSSIoverallsupportindexersterZP	Varianzen sind gleich	,073	,788	-,671
	Varianzen sind nicht gleich			-,679

Test bei unabhängigen Stichproben

		t-Test für die Mittelwertgleichheit		
		df	Sig. (2-seitig)	Mittlere Differenz
SSSIoverallsupportindexersterZP	Varianzen sind gleich	40	,506	-,20137
	Varianzen sind nicht gleich	39,819	,501	-,20137

Test bei unabhängigen Stichproben

		t-Test für die Mittelwertgleichheit	
			95% Konfidenzinterv..
		Differenz für Standardfehler	Unterer Wert
SSSIoverallsupportindexersterZP	Varianzen sind gleich	,30010	-,80790
	Varianzen sind nicht gleich	,29638	-,80047

Test bei unabhängigen Stichproben

		t-Test für die Mittelwertgleichh..
		95% Konfidenzinterval..
		Oberer Wert
SSSIoverallsupportindexersterZP	Varianzen sind gleich	,40516
	Varianzen sind nicht gleich	,39773

Effektgrößen bei unabhängigen Stichproben

		Standardisierer[a]	Punktschätzung	95% ... Unterer Wert
SSSIoverallsupportindexersterZP	Cohen's d	,96802	-,208	-,816
	Hedges' Korrektur	,98666	-,204	-,801
	Glass' Delta	,89834	-,224	-,833

Effektgrößen bei unabhängigen Stichproben

		95% ... Oberer Wert
SSSIoverallsupportindexersterZP	Cohen's d	,403
	Hedges' Korrektur	,395
	Glass' Delta	,391

a. Der bei der Schätzung der Effektgrößen verwendete Nenner.
Cohen's d verwendet die zusammengefasste Standardabweichung.
Hedges' Korrektur verwendet die zusammengefasste Standardabweichung und einen Korrekturfaktor.
Glass' Delta verwendet die Standardabweichung einer Stichprobe von der Kontrollgruppe.

```
T-TEST GROUPS=STAITraitMedianaufteilungErsterZP(1 2)
  /MISSING=ANALYSIS
  /VARIABLES=BRCSersterZP
  /ES DISPLAY(TRUE)
  /CRITERIA=CI(.95).
```

t-Test

Gruppenstatistiken

	STAITraitMedianaufteilung ErsterZP	N	Mittelwert	Std.-Abweichung	Standardfehler des Mittelwertes
BRCSersterZP	1bis40	23	14,5652	3,13087	,65283
	ab41	21	13,5714	3,41426	,74505

Test bei unabhängigen Stichproben

		Levene-Test der Varianzgleichheit F	Sig.	t-Test für die Mittelwertgleichheit T	df
BRCSersterZP	Varianzen sind gleich	,661	,421	1,007	42
	Varianzen sind nicht gleich			1,003	40,693

Test bei unabhängigen Stichproben

		t-Test für die Mittelwertgleichheit		
		Sig. (2-seitig)	Mittlere Differenz	Differenz für Standardfehler
BRCSersterZP	Varianzen sind gleich	,320	,99379	,98663
	Varianzen sind nicht gleich	,322	,99379	,99060

Test bei unabhängigen Stichproben

		t-Test für die Mittelwertgleichheit	
		95% Konfidenzintervall der Differenz	
		Unterer Wert	Oberer Wert
BRCSersterZP	Varianzen sind gleich	-,99730	2,98488
	Varianzen sind nicht gleich	-1,00723	2,99481

Effektgrößen bei unabhängigen Stichproben

		Standardisierer[a]	Punktschätzung	95% Konfidenzintervall	
				Unterer Wert	Oberer Wert
BRCSersterZP	Cohen's d	3,26888	,304	-,293	,897
	Hedges' Korrektur	3,32874	,299	-,288	,881
	Glass' Delta	3,41426	,291	-,311	,886

a. Der bei der Schätzung der Effektgrößen verwendete Nenner.
Cohen's d verwendet die zusammengefasste Standardabweichung.
Hedges' Korrektur verwendet die zusammengefasste Standardabweichung und einen Korrekturfaktor.
Glass' Delta verwendet die Standardabweichung einer Stichprobe von der Kontrollgruppe.

```
T-TEST GROUPS=STAITraitMedianaufteilungErsterZP(1 2)
  /MISSING=ANALYSIS
  /VARIABLES=AVEMzweiterZP
  /ES DISPLAY(TRUE)
  /CRITERIA=CI(.95).
```

t-Test

Gruppenstatistiken

	STAITraitMedianaufteilung ErsterZP	N	Mittelwert	Std.-Abweichung	Standardfehler des Mittelwertes
AVEMzweiterZP	1bis40	11	5,4545	1,86353	,56187
	ab41	11	4,5455	2,76997	,83518

Test bei unabhängigen Stichproben

		Levene-Test der Varianzgleichheit		t-Test für die Mittelwertgleichheit	
		F	Sig.	T	df
AVEMzweiterZP	Varianzen sind gleich	1,278	,272	,903	20
	Varianzen sind nicht gleich			,903	17,513

Test bei unabhängigen Stichproben

		t-Test für die Mittelwertgleichheit		
		Sig. (2-seitig)	Mittlere Differenz	Differenz für Standardfehler
AVEMzweiterZP	Varianzen sind gleich	,377	,90909	1,00659
	Varianzen sind nicht gleich	,379	,90909	1,00659

Test bei unabhängigen Stichproben

		t-Test für die Mittelwertgleichheit 95% Konfidenzintervall der Differenz	
		Unterer Wert	Oberer Wert
AVEMzweiterZP	Varianzen sind gleich	-1,19062	3,00880
	Varianzen sind nicht gleich	-1,20990	3,02808

Effektgrößen bei unabhängigen Stichproben

		Standardisierer[a]	Punktschätzung	95% Konfidenzintervall	
				Unterer Wert	Oberer Wert
AVEMzweiterZP	Cohen's d	2,36066	,385	-,464	1,225
	Hedges' Korrektur	2,45405	,370	-,446	1,178
	Glass' Delta	2,76997	,328	-,527	1,168

a. Der bei der Schätzung der Effektgrößen verwendete Nenner.
Cohen's d verwendet die zusammengefasste Standardabweichung.
Hedges' Korrektur verwendet die zusammengefasste Standardabweichung und einen Korrekturfaktor.
Glass' Delta verwendet die Standardabweichung einer Stichprobe von der Kontrollgruppe.

```
T-TEST GROUPS=STAITraitMedianaufteilungErsterZP(1 2)
  /MISSING=ANALYSIS
  /VARIABLES=ISIersterZP
  /ES DISPLAY(TRUE)
  /CRITERIA=CI(.95).
```

t-Test

Gruppenstatistiken

	STAITraitMedianaufteilung ErsterZP	N	Mittelwert	Std.-Abweichung	Standardfehler des Mittelwertes
ISIersterZP	1bis40	23	9,1304	5,91842	1,23408
	ab41	21	13,1429	4,13867	,90313

Test bei unabhängigen Stichproben

		Levene-Test der Varianzgleichheit		t-Test für die Mittelwertgleichheit	
		F	Sig.	T	df
ISIersterZP	Varianzen sind gleich	3,216	,080	-2,582	42
	Varianzen sind nicht gleich			-2,624	39,434

Test bei unabhängigen Stichproben

		t-Test für die Mittelwertgleichheit		
		Sig. (2-seitig)	Mittlere Differenz	Differenz für Standardfehler
ISIersterZP	Varianzen sind gleich	,013	-4,01242	1,55386
	Varianzen sind nicht gleich	,012	-4,01242	1,52924

Test bei unabhängigen Stichproben

		t-Test für die Mittelwertgleichheit: 95% Konfidenzintervall der Differenz	
		Unterer Wert	Oberer Wert
ISIersterZP	Varianzen sind gleich	-7,14823	-,87661
	Varianzen sind nicht gleich	-7,10452	-,92032

Effektgrößen bei unabhängigen Stichproben

		Standardisierer[a]	Punktschätzung	95% Konfidenzintervall	
				Unterer Wert	Oberer Wert
ISIersterZP	Cohen's d	5,14823	-,779	-1,390	-,161
	Hedges' Korrektur	5,24250	-,765	-1,365	-,158
	Glass' Delta	4,13867	-,969	-1,622	-,297

a. Der bei der Schätzung der Effektgrößen verwendete Nenner.
Cohen's d verwendet die zusammengefasste Standardabweichung.
Hedges' Korrektur verwendet die zusammengefasste Standardabweichung und einen Korrekturfaktor.
Glass' Delta verwendet die Standardabweichung einer Stichprobe von der Kontrollgruppe.

```
T-TEST GROUPS=STAITraitMedianaufteilungErsterZP(1 2)
  /MISSING=ANALYSIS
  /VARIABLES=ISIzweiterZP
  /ES DISPLAY(TRUE)
  /CRITERIA=CI(.95).
```

t-Test

Gruppenstatistiken

	STAITraitMedianaufteilung ErsterZP	N	Mittelwert	Std.-Abweichung	Standardfehler des Mittelwertes
ISIzweiterZP	1bis40	13	8,3846	5,60563	1,55472
	ab41	14	12,6429	5,78602	1,54638

Test bei unabhängigen Stichproben

		Levene-Test der Varianzgleichheit F	Levene-Test der Varianzgleichheit Sig.	t-Test für die Mittelwertgleichheit T	t-Test für die Mittelwertgleichheit df
ISIzweiterZP	Varianzen sind gleich	,054	,818	-1,940	25
	Varianzen sind nicht gleich			-1,942	24,949

Test bei unabhängigen Stichproben

		t-Test für die Mittelwertgleichheit		
		Sig. (2-seitig)	Mittlere Differenz	Differenz für Standardfehler
ISIzweiterZP	Varianzen sind gleich	,064	-4,25824	2,19549
	Varianzen sind nicht gleich	,064	-4,25824	2,19282

Test bei unabhängigen Stichproben

		t-Test für die Mittelwertgleichheit	
		95% Konfidenzintervall der Differenz	
		Unterer Wert	Oberer Wert
ISIzweiterZP	Varianzen sind gleich	-8,77994	,26346
	Varianzen sind nicht gleich	-8,77491	,25842

Effektgrößen bei unabhängigen Stichproben

		Standardisierer[a]	Punktschätzung	95% Konfidenzintervall Unterer Wert	95% Konfidenzintervall Oberer Wert
ISIzweiterZP	Cohen's d	5,70014	-,747	-1,523	,042
	Hedges' Korrektur	5,87859	-,724	-1,476	,041
	Glass' Delta	5,78602	-,736	-1,529	,082

a. Der bei der Schätzung der Effektgrößen verwendete Nenner.
Cohen's d verwendet die zusammengefasste Standardabweichung.
Hedges' Korrektur verwendet die zusammengefasste Standardabweichung und einen Korrekturfaktor.
Glass' Delta verwendet die Standardabweichung einer Stichprobe von der Kontrollgruppe.

```
T-TEST GROUPS=STAITraitMedianaufteilungErsterZP(1 2)
  /MISSING=ANALYSIS
  /VARIABLES=ESSersterZP
```

```
/ES DISPLAY(TRUE)
/CRITERIA=CI(.95).
```

t-Test

Gruppenstatistiken

	STAITraitMedianaufteilung ErsterZP	N	Mittelwert	Std.-Abweichung	Standardfehler des Mittelwertes
ESSersterZP	1bis40	23	8,6957	4,39457	,91633
	ab41	21	10,5238	3,98270	,86910

Test bei unabhängigen Stichproben

		Levene-Test der Varianzgleichheit		t-Test für die Mittelwertgleichheit	
		F	Sig.	T	df
ESSersterZP	Varianzen sind gleich	,127	,723	-1,441	42
	Varianzen sind nicht gleich			-1,448	41,999

Test bei unabhängigen Stichproben

		t-Test für die Mittelwertgleichheit		
		Sig. (2-seitig)	Mittlere Differenz	Differenz für Standardfehler
ESSersterZP	Varianzen sind gleich	,157	-1,82816	1,26871
	Varianzen sind nicht gleich	,155	-1,82816	1,26293

Test bei unabhängigen Stichproben

		t-Test für die Mittelwertgleichheit	
		95% Konfidenzintervall der Differenz	
		Unterer Wert	Oberer Wert
ESSersterZP	Varianzen sind gleich	-4,38851	,73220
	Varianzen sind nicht gleich	-4,37686	,72054

Effektgrößen bei unabhängigen Stichproben

		Standardisierer[a]	Punktschätzung	95% Konfidenzintervall	
				Unterer Wert	Oberer Wert
ESSersterZP	Cohen's d	4,20348	-,435	-1,031	,166
	Hedges' Korrektur	4,28045	-,427	-1,013	,163
	Glass' Delta	3,98270	-,459	-1,062	,155

a. Der bei der Schätzung der Effektgrößen verwendete Nenner.
Cohen's d verwendet die zusammengefasste Standardabweichung.
Hedges' Korrektur verwendet die zusammengefasste Standardabweichung und einen Korrekturfaktor.
Glass' Delta verwendet die Standardabweichung einer Stichprobe von der Kontrollgruppe.

```
T-TEST GROUPS=STAITraitMedianaufteilungErsterZP(1 2)
  /MISSING=ANALYSIS
  /VARIABLES=ESSzweiterZP
  /ES DISPLAY(TRUE)
  /CRITERIA=CI(.95).
```

t-Test

Gruppenstatistiken

	STAITraitMedianaufteilung ErsterZP	N	Mittelwert	Std.-Abweichung	Standardfehler des Mittelwertes
ESSzweiterZP	1bis40	13	10,0769	5,05736	1,40266
	ab41	14	11,1429	4,68807	1,25294

Test bei unabhängigen Stichproben

		Levene-Test der Varianzgleichheit		t-Test für die Mittelwertgleichheit	
		F	Sig.	T	df
ESSzweiterZP	Varianzen sind gleich	,494	,489	-,568	25
	Varianzen sind nicht gleich			-,567	24,432

Test bei unabhängigen Stichproben

		t-Test für die Mittelwertgleichheit		
		Sig. (2-seitig)	Mittlere Differenz	Differenz für Standardfehler
ESSzweiterZP	Varianzen sind gleich	,575	-1,06593	1,87530
	Varianzen sind nicht gleich	,576	-1,06593	1,88077

Test bei unabhängigen Stichproben

		t-Test für die Mittelwertgleichheit	
		95% Konfidenzintervall der Differenz	
		Unterer Wert	Oberer Wert
ESSzweiterZP	Varianzen sind gleich	-4,92819	2,79632
	Varianzen sind nicht gleich	-4,94404	2,81217

Effektgrößen bei unabhängigen Stichproben

		Standardisierer[a]	Punktschätzung	95% Konfidenzintervall	
				Unterer Wert	Oberer Wert
ESSzweiterZP	Cohen's d	4,86883	-,219	-,974	,541
	Hedges' Korrektur	5,02125	-,212	-,945	,524
	Glass' Delta	4,68807	-,227	-,983	,537

a. Der bei der Schätzung der Effektgrößen verwendete Nenner.
Cohen's d verwendet die zusammengefasste Standardabweichung.
Hedges' Korrektur verwendet die zusammengefasste Standardabweichung und einen Korrekturfaktor.
Glass' Delta verwendet die Standardabweichung einer Stichprobe von der Kontrollgruppe.

```
T-TEST GROUPS=STAITraitMedianaufteilungErsterZP(1 2)
  /MISSING=ANALYSIS
  /VARIABLES=PSSersterZP
  /ES DISPLAY(TRUE)
  /CRITERIA=CI(.95).
```

t-Test

Gruppenstatistiken

	STAITraitMedianaufteilung ErsterZP	N	Mittelwert	Std.-Abweichung	Standardfehler des Mittelwertes
PSSersterZP	1bis40	23	16,0435	4,12837	,86083
	ab41	21	23,8571	5,04268	1,10040

Test bei unabhängigen Stichproben

		Levene-Test der Varianzgleichheit		t-Test für die Mittelwertgleichheit	
		F	Sig.	T	df
PSSersterZP	Varianzen sind gleich	2,585	,115	-5,644	42
	Varianzen sind nicht gleich			-5,593	38,769

Test bei unabhängigen Stichproben

		t-Test für die Mittelwertgleichheit		
		Sig. (2-seitig)	Mittlere Differenz	Differenz für Standardfehler
PSSersterZP	Varianzen sind gleich	,000	-7,81366	1,38433
	Varianzen sind nicht gleich	,000	-7,81366	1,39711

Test bei unabhängigen Stichproben

		t-Test für die Mittelwertgleichheit	
		95% Konfidenzintervall der Differenz	
		Unterer Wert	Oberer Wert
PSSersterZP	Varianzen sind gleich	-10,60735	-5,01998
	Varianzen sind nicht gleich	-10,64012	-4,98721

Effektgrößen bei unabhängigen Stichproben

		Standardisierer[a]	Punktschätzung	95% Konfidenzintervall	
				Unterer Wert	Oberer Wert
PSSersterZP	Cohen's d	4,58654	-1,704	-2,390	-1,002
	Hedges' Korrektur	4,67053	-1,673	-2,347	-,984
	Glass' Delta	5,04268	-1,550	-2,298	-,778

a. Der bei der Schätzung der Effektgrößen verwendete Nenner.
Cohen's d verwendet die zusammengefasste Standardabweichung.
Hedges' Korrektur verwendet die zusammengefasste Standardabweichung und einen Korrekturfaktor.
Glass' Delta verwendet die Standardabweichung einer Stichprobe von der Kontrollgruppe.

```
T-TEST GROUPS=STAITraitMedianaufteilungErsterZP(1 2)
  /MISSING=ANALYSIS
  /VARIABLES=PSSzweiterZP
```

```
/ES DISPLAY(TRUE)
/CRITERIA=CI(.95).
```

t-Test

Gruppenstatistiken

	STAITraitMedianaufteilung ErsterZP	N	Mittelwert	Std.-Abweichung	Standardfehler des Mittelwertes
PSSzweiterZP	1bis40	13	14,6923	4,62574	1,28295
	ab41	14	24,0714	5,13606	1,37267

Test bei unabhängigen Stichproben

		Levene-Test der Varianzgleichheit		t-Test für die Mittelwertgleichheit	
		F	Sig.	T	df
PSSzweiterZP	Varianzen sind gleich	,371	,548	-4,972	25
	Varianzen sind nicht gleich			-4,992	24,981

Test bei unabhängigen Stichproben

		t-Test für die Mittelwertgleichheit		
		Sig. (2-seitig)	Mittlere Differenz	Differenz für Standardfehler
PSSzweiterZP	Varianzen sind gleich	,000	-9,37912	1,88644
	Varianzen sind nicht gleich	,000	-9,37912	1,87888

Test bei unabhängigen Stichproben

		t-Test für die Mittelwertgleichheit	
		95% Konfidenzintervall der Differenz	
		Unterer Wert	Oberer Wert
PSSzweiterZP	Varianzen sind gleich	-13,26431	-5,49393
	Varianzen sind nicht gleich	-13,24889	-5,50935

Effektgrößen bei unabhängigen Stichproben

		Standardisierer[a]	Punktschätzung	95% Konfidenzintervall Unterer Wert	Oberer Wert
PSSzweiterZP	Cohen's d	4,89775	-1,915	-2,823	-,981
	Hedges' Korrektur	5,05107	-1,857	-2,738	-,951
	Glass' Delta	5,13606	-1,826	-2,833	-,782

a. Der bei der Schätzung der Effektgrößen verwendete Nenner.
Cohen's d verwendet die zusammengefasste Standardabweichung.
Hedges' Korrektur verwendet die zusammengefasste Standardabweichung und einen Korrekturfaktor.
Glass' Delta verwendet die Standardabweichung einer Stichprobe von der Kontrollgruppe.

```
T-TEST GROUPS=STAITraitMedianaufteilungErsterZP(1 2)
  /MISSING=ANALYSIS
  /VARIABLES=GBBGesamtskalaBersterZP
  /ES DISPLAY(TRUE)
  /CRITERIA=CI(.95).
```

t-Test

Gruppenstatistiken

	STAITraitMedianaufteilung ErsterZP	N	Mittelwert	Std.-Abweichung
GBBGesamtskalaBersterZP	1bis40	22	22,4091	11,79451
	ab41	21	22,7143	13,03128

Gruppenstatistiken

	STAITraitMedianaufteilung ErsterZP	Standardfehler des Mittelwertes
GBBGesamtskalaBersterZP	1bis40	2,51460
	ab41	2,84366

Test bei unabhängigen Stichproben

		Levene-Test der Varianzgleichheit F	Sig.	t-Test für die ... T
GBBGesamtskalaBersterZP	Varianzen sind gleich	1,671	,203	-,081
	Varianzen sind nicht gleich			-,080

Test bei unabhängigen Stichproben

		t-Test für die Mittelwertgleichheit		
		df	Sig. (2-seitig)	Mittlere Differenz
GBBGesamtskalaBersterZP	Varianzen sind gleich	41	,936	-,30519
	Varianzen sind nicht gleich	40,135	,936	-,30519

Test bei unabhängigen Stichproben

		t-Test für die Mittelwertgleichheit	
			95% Konfidenzinterv..
		Differenz für Standardfehler	Unterer Wert
GBBGesamtskalaBersterZP	Varianzen sind gleich	3,78702	-7,95324
	Varianzen sind nicht gleich	3,79600	-7,97639

Test bei unabhängigen Stichproben

		t-Test für die Mittelwertgleichh..
		95% Konfidenzinterval..
		Oberer Wert
GBBGesamtskalaBersterZP	Varianzen sind gleich	7,34285
	Varianzen sind nicht gleich	7,36600

Effektgrößen bei unabhängigen Stichproben

		Standardisierer[a]	Punktschätzung	95% ...
				Unterer Wert
GBBGesamtskalaBersterZP	Cohen's d	12,41321	-,025	-,622
	Hedges' Korrektur	12,64621	-,024	-,611
	Glass' Delta	13,03128	-,023	-,621

Effektgrößen bei unabhängigen Stichproben

		95% ...
		Oberer Wert
GBBGesamtskalaBersterZP	Cohen's d	,574
	Hedges' Korrektur	,563
	Glass' Delta	,575

a. Der bei der Schätzung der Effektgrößen verwendete Nenner.
Cohen's d verwendet die zusammengefasste Standardabweichung.
Hedges' Korrektur verwendet die zusammengefasste Standardabweichung und einen Korrekturfaktor.
Glass' Delta verwendet die Standardabweichung einer Stichprobe von der Kontrollgruppe.

```
T-TEST GROUPS=STAITraitMedianaufteilungErsterZP(1 2)
  /MISSING=ANALYSIS
  /VARIABLES=GBBGesamtskalaBzweiterZP
  /ES DISPLAY(TRUE)
  /CRITERIA=CI(.95).
```

t-Test

Gruppenstatistiken

	STAITraitMedianaufteilung ErsterZP	N	Mittelwert	Std.-Abweichung
GBBGesamtskalaBzweiterZP	1bis40	12	22,0000	8,48528
	ab41	14	25,7857	13,66173

Gruppenstatistiken

	STAITraitMedianaufteilung ErsterZP	Standardfehler des Mittelwertes
GBBGesamtskalaBzweiterZP	1bis40	2,44949
	ab41	3,65125

Test bei unabhängigen Stichproben

		Levene-Test der Varianzgleichheit		t-Test für die ...
		F	Sig.	T
GBBGesamtskalaBzweiterZP	Varianzen sind gleich	4,369	,047	-,831
	Varianzen sind nicht gleich			-,861

Test bei unabhängigen Stichproben

		t-Test für die Mittelwertgleichheit		
		df	Sig. (2-seitig)	Mittlere Differenz
GBBGesamtskalaBzweiterZP	Varianzen sind gleich	24	,414	-3,78571
	Varianzen sind nicht gleich	22,055	,398	-3,78571

Test bei unabhängigen Stichproben

		t-Test für die Mittelwertgleichheit	
			95% Konfidenzinterv..
		Differenz für Standardfehler	Unterer Wert
GBBGesamtskalaBzweiterZP	Varianzen sind gleich	4,55558	-13,18797
	Varianzen sind nicht gleich	4,39678	-12,90275

Test bei unabhängigen Stichproben

		t-Test für die Mittelwertgleichh..
		95% Konfidenzinterval..
		Oberer Wert
GBBGesamtskalaBzweiterZP	Varianzen sind gleich	5,61654
	Varianzen sind nicht gleich	5,33132

Effektgrößen bei unabhängigen Stichproben

		Standardisierer[a]		95% ...
			Punktschätzung	Unterer Wert
GBBGesamtskalaBzweiterZP	Cohen's d	11,58008	-,327	-1,100
	Hedges' Korrektur	11,95838	-,317	-1,065
	Glass' Delta	13,66173	-,277	-1,050

Effektgrößen bei unabhängigen Stichproben

		95% ...
		Oberer Wert
GBBGesamtskalaBzweiterZP	Cohen's d	,453
	Hedges' Korrektur	,439
	Glass' Delta	,506

a. Der bei der Schätzung der Effektgrößen verwendete Nenner.
Cohen's d verwendet die zusammengefasste Standardabweichung.
Hedges' Korrektur verwendet die zusammengefasste Standardabweichung und einen Korrekturfaktor.
Glass' Delta verwendet die Standardabweichung einer Stichprobe von der Kontrollgruppe.

```
T-TEST GROUPS=STAITraitMedianaufteilungErsterZP(1 2)
  /MISSING=ANALYSIS
  /VARIABLES=TICSersterZPsscs
  /ES DISPLAY(TRUE)
  /CRITERIA=CI(.95).
```

t-Test

Gruppenstatistiken

	STAITraitMedianaufteilung ErsterZP	N	Mittelwert	Std.-Abweichung
TICSersterZPsscs	1bis40	22	19,5455	7,88445
	ab41	20	16,2000	7,35992

Gruppenstatistiken

	STAITraitMedianaufteilung ErsterZP	Standardfehler des Mittelwertes
TICSersterZPsscs	1bis40	1,68097
	ab41	1,64573

Test bei unabhängigen Stichproben

		Levene-Test der Varianzgleichheit		t-Test für die ...
		F	Sig.	T
TICSersterZPsscs	Varianzen sind gleich	,104	,749	1,417
	Varianzen sind nicht gleich			1,422

Test bei unabhängigen Stichproben

		t-Test für die Mittelwertgleichheit		
		df	Sig. (2-seitig)	Mittlere Differenz
TICSersterZPsscs	Varianzen sind gleich	40	,164	3,34545
	Varianzen sind nicht gleich	39,967	,163	3,34545

Test bei unabhängigen Stichproben

		t-Test für die Mittelwertgleichheit		
			95% Konfidenzintervall der Differenz	
		Differenz für Standardfehler	Unterer Wert	Oberer Wert
TICSersterZPsscs	Varianzen sind gleich	2,36037	-1,42503	8,11594
	Varianzen sind nicht gleich	2,35246	-1,40917	8,10008

Effektgrößen bei unabhängigen Stichproben

		Standardisierer[a]	Punktschätzung	95% ... Unterer Wert	95% ... Oberer Wert
TICSersterZPsscs	Cohen's d	7,63979	,438	-,178	1,048
	Hedges' Korrektur	7,78686	,430	-,174	1,028
	Glass' Delta	7,35992	,455	-,173	1,071

a. Der bei der Schätzung der Effektgrößen verwendete Nenner.
Cohen's d verwendet die zusammengefasste Standardabweichung.
Hedges' Korrektur verwendet die zusammengefasste Standardabweichung und einen Korrekturfaktor.
Glass' Delta verwendet die Standardabweichung einer Stichprobe von der Kontrollgruppe.

```
T-TEST GROUPS=STAITraitMedianaufteilungErsterZP(1 2)
  /MISSING=ANALYSIS
  /VARIABLES=TICSzweiterZPsscs
  /ES DISPLAY(TRUE)
  /CRITERIA=CI(.95).
```

t-Test

Gruppenstatistiken

	STAITraitMedianaufteilung ErsterZP	N	Mittelwert	Std.-Abweichung	Standardfehler des Mittelwertes
TICSzweiterZPsscs	1bis40	12	11,7500	6,19567	1,78854
	ab41	14	21,1429	10,78359	2,88203

Test bei unabhängigen Stichproben

		Levene-Test der Varianzgleichheit		t-Test für die ...
		F	Sig.	T
TICSzweiterZPsscs	Varianzen sind gleich	3,218	,085	-2,660
	Varianzen sind nicht gleich			-2,769

Test bei unabhängigen Stichproben

		t-Test für die Mittelwertgleichheit		
		df	Sig. (2-seitig)	Mittlere Differenz
TICSzweiterZPsscs	Varianzen sind gleich	24	,014	-9,39286
	Varianzen sind nicht gleich	21,221	,011	-9,39286

Test bei unabhängigen Stichproben

		t-Test für die Mittelwertgleichheit		
		Differenz für Standardfehler	95% Konfidenzintervall der Differenz	
			Unterer Wert	Oberer Wert
TICSzweiterZPsscs	Varianzen sind gleich	3,53143	-16,68137	-2,10434
	Varianzen sind nicht gleich	3,39190	-16,44222	-2,34349

Effektgrößen bei unabhängigen Stichproben

		Standardisierer[a]	Punktschätzung	95% ... Unterer Wert
TICSzweiterZPsscs	Cohen's d	8,97674	-1,046	-1,862
	Hedges' Korrektur	9,26999	-1,013	-1,803
	Glass' Delta	10,78359	-,871	-1,696

Effektgrößen bei unabhängigen Stichproben

		95% ... Oberer Wert
TICSzweiterZPsscs	Cohen's d	-,212
	Hedges' Korrektur	-,205
	Glass' Delta	-,018

a. Der bei der Schätzung der Effektgrößen verwendete Nenner.
Cohen's d verwendet die zusammengefasste Standardabweichung.
Hedges' Korrektur verwendet die zusammengefasste Standardabweichung und einen Korrekturfaktor.
Glass' Delta verwendet die Standardabweichung einer Stichprobe von der Kontrollgruppe.

```
T-TEST GROUPS=STAITraitMedianaufteilungErsterZP(1 2)
  /MISSING=ANALYSIS
  /VARIABLES=MBIeeersterZP
  /ES DISPLAY(TRUE)
  /CRITERIA=CI(.95).
```

t-Test

Gruppenstatistiken

	STAITraitMedianaufteilung ErsterZP	N	Mittelwert	Std.-Abweichung	Standardfehler des Mittelwertes
MBIeeersterZP	1bis40	23	13,7391	9,99189	2,08345
	ab41	18	25,1111	10,47062	2,46795

Test bei unabhängigen Stichproben

		Levene-Test der Varianzgleichheit		t-Test für die Mittelwertgleichheit	
		F	Sig.	T	df
MBIeeersterZP	Varianzen sind gleich	,126	,724	-3,542	39
	Varianzen sind nicht gleich			-3,521	35,811

Test bei unabhängigen Stichproben

		t-Test für die Mittelwertgleichheit		
		Sig. (2-seitig)	Mittlere Differenz	Differenz für Standardfehler
MBIeeersterZP	Varianzen sind gleich	,001	-11,37198	3,21095
	Varianzen sind nicht gleich	,001	-11,37198	3,22979

Test bei unabhängigen Stichproben

		t-Test für die Mittelwertgleichheit	
		95% Konfidenzintervall der Differenz	
		Unterer Wert	Oberer Wert
MBIeeersterZP	Varianzen sind gleich	-17,86674	-4,87722
	Varianzen sind nicht gleich	-17,92351	-4,82046

Effektgrößen bei unabhängigen Stichproben

		Standardisierer[a]	Punktschätzung	95% Konfidenzintervall	
				Unterer Wert	Oberer Wert
MBIeeersterZP	Cohen's d	10,20333	-1,115	-1,773	-,444
	Hedges' Korrektur	10,40494	-1,093	-1,738	-,436
	Glass' Delta	10,47062	-1,086	-1,790	-,359

a. Der bei der Schätzung der Effektgrößen verwendete Nenner.
Cohen's d verwendet die zusammengefasste Standardabweichung.
Hedges' Korrektur verwendet die zusammengefasste Standardabweichung und einen Korrekturfaktor.
Glass' Delta verwendet die Standardabweichung einer Stichprobe von der Kontrollgruppe.

```
T-TEST GROUPS=STAITraitMedianaufteilungErsterZP(1 2)
  /MISSING=ANALYSIS
  /VARIABLES=MBIeezweiterZP
  /ES DISPLAY(TRUE)
  /CRITERIA=CI(.95).
```

t-Test

Gruppenstatistiken

	STAITraitMedianaufteilung ErsterZP	N	Mittelwert	Std.-Abweichung	Standardfehler des Mittelwertes
MBIeezweiterZP	1bis40	13	15,0769	12,77668	3,54361
	ab41	13	22,6923	12,18869	3,38053

Test bei unabhängigen Stichproben

		Levene-Test der Varianzgleichheit		t-Test für die Mittelwertgleichheit	
		F	Sig.	T	df
MBleezweiterZP	Varianzen sind gleich	,048	,828	-1,555	24
	Varianzen sind nicht gleich			-1,555	23,947

Test bei unabhängigen Stichproben

		t-Test für die Mittelwertgleichheit		
		Sig. (2-seitig)	Mittlere Differenz	Differenz für Standardfehler
MBleezweiterZP	Varianzen sind gleich	,133	-7,61538	4,89747
	Varianzen sind nicht gleich	,133	-7,61538	4,89747

Test bei unabhängigen Stichproben

		t-Test für die Mittelwertgleichheit	
		95% Konfidenzintervall der Differenz	
		Unterer Wert	Oberer Wert
MBleezweiterZP	Varianzen sind gleich	-17,72326	2,49250
	Varianzen sind nicht gleich	-17,72445	2,49368

Effektgrößen bei unabhängigen Stichproben

		Standardisierer[a]	Punktschätzung	95% Konfidenzintervall	
				Unterer Wert	Oberer Wert
MBleezweiterZP	Cohen's d	12,48615	-,610	-1,392	,184
	Hedges' Korrektur	12,89405	-,591	-1,348	,178
	Glass' Delta	12,18869	-,625	-1,421	,194

a. Der bei der Schätzung der Effektgrößen verwendete Nenner.
Cohen's d verwendet die zusammengefasste Standardabweichung.
Hedges' Korrektur verwendet die zusammengefasste Standardabweichung und einen Korrekturfaktor.
Glass' Delta verwendet die Standardabweichung einer Stichprobe von der Kontrollgruppe.

```
T-TEST GROUPS=STAITraitMedianaufteilungErsterZP(1 2)
  /MISSING=ANALYSIS
  /VARIABLES=MBIdersterZP
```

```
/ES DISPLAY(TRUE)
/CRITERIA=CI(.95).
```

t-Test

Gruppenstatistiken

	STAITraitMedianaufteilung ErsterZP	N	Mittelwert	Std.-Abweichung	Standardfehler des Mittelwertes
MBIdersterZP	1bis40	23	1,2174	1,44463	,30123
	ab41	18	4,9444	4,16529	,98177

Test bei unabhängigen Stichproben

		Levene-Test der Varianzgleichheit		t-Test für die Mittelwertgleichheit	
		F	Sig.	T	df
MBIdersterZP	Varianzen sind gleich	31,990	,000	-4,006	39
	Varianzen sind nicht gleich			-3,629	20,213

Test bei unabhängigen Stichproben

		t-Test für die Mittelwertgleichheit		
		Sig. (2-seitig)	Mittlere Differenz	Differenz für Standardfehler
MBIdersterZP	Varianzen sind gleich	,000	-3,72705	,93035
	Varianzen sind nicht gleich	,002	-3,72705	1,02694

Test bei unabhängigen Stichproben

		t-Test für die Mittelwertgleichheit 95% Konfidenzintervall der Differenz	
		Unterer Wert	Oberer Wert
MBIdersterZP	Varianzen sind gleich	-5,60886	-1,84524
	Varianzen sind nicht gleich	-5,86777	-1,58634

Effektgrößen bei unabhängigen Stichproben

		Standardisierer[a]	Punktschätzung	95% Konfidenzintervall	
				Unterer Wert	Oberer Wert
MBIdersterZP	Cohen's d	2,95634	-1,261	-1,931	-,577
	Hedges' Korrektur	3,01475	-1,236	-1,893	-,566
	Glass' Delta	4,16529	-,895	-1,569	-,199

a. Der bei der Schätzung der Effektgrößen verwendete Nenner.
Cohen's d verwendet die zusammengefasste Standardabweichung.
Hedges' Korrektur verwendet die zusammengefasste Standardabweichung und einen Korrekturfaktor.
Glass' Delta verwendet die Standardabweichung einer Stichprobe von der Kontrollgruppe.

```
T-TEST GROUPS=STAITraitMedianaufteilungErsterZP(1 2)
  /MISSING=ANALYSIS
  /VARIABLES=MBIdzweiterZP
  /ES DISPLAY(TRUE)
  /CRITERIA=CI(.95).
```

t-Test

Gruppenstatistiken

	STAITraitMedianaufteilung ErsterZP	N	Mittelwert	Std.-Abweichung	Standardfehler des Mittelwertes
MBIdzweiterZP	1bis40	13	2,3846	2,25605	,62571
	ab41	13	5,4615	4,66575	1,29405

Test bei unabhängigen Stichproben

		Levene-Test der Varianzgleichheit		t-Test für die Mittelwertgleichheit	
		F	Sig.	T	df
MBIdzweiterZP	Varianzen sind gleich	3,497	,074	-2,141	24
	Varianzen sind nicht gleich			-2,141	17,320

Test bei unabhängigen Stichproben

		t-Test für die Mittelwertgleichheit		
		Sig. (2-seitig)	Mittlere Differenz	Differenz für Standardfehler
MBIdzweiterZP	Varianzen sind gleich	,043	-3,07692	1,43738
	Varianzen sind nicht gleich	,047	-3,07692	1,43738

Test bei unabhängigen Stichproben

		t-Test für die Mittelwertgleichheit	
		95% Konfidenzintervall der Differenz	
		Unterer Wert	Oberer Wert
MBIdzweiterZP	Varianzen sind gleich	-6,04354	-,11031
	Varianzen sind nicht gleich	-6,10527	-,04857

Effektgrößen bei unabhängigen Stichproben

		Standardisierer[a]	Punktschätzung	95% Konfidenzintervall	
				Unterer Wert	Oberer Wert
MBIdzweiterZP	Cohen's d	3,66463	-,840	-1,636	-,027
	Hedges' Korrektur	3,78434	-,813	-1,584	-,027
	Glass' Delta	4,66575	-,659	-1,459	,165

a. Der bei der Schätzung der Effektgrößen verwendete Nenner.
Cohen's d verwendet die zusammengefasste Standardabweichung.
Hedges' Korrektur verwendet die zusammengefasste Standardabweichung und einen Korrekturfaktor.
Glass' Delta verwendet die Standardabweichung einer Stichprobe von der Kontrollgruppe.

```
T-TEST GROUPS=STAITraitMedianaufteilungErsterZP(1 2)
  /MISSING=ANALYSIS
  /VARIABLES=MBIpaersterZP
  /ES DISPLAY(TRUE)
  /CRITERIA=CI(.95).
```

t-Test

Gruppenstatistiken

	STAITraitMedianaufteilung ErsterZP	N	Mittelwert	Std.-Abweichung	Standardfehler des Mittelwertes
MBIpaersterZP	1bis40	23	38,9130	6,81518	1,42106
	ab41	18	37,9444	8,22101	1,93771

Test bei unabhängigen Stichproben

		Levene-Test der Varianzgleichheit F	Levene-Test der Varianzgleichheit Sig.	t-Test für die Mittelwertgleichheit T	t-Test für die Mittelwertgleichheit df
MBIpaersterZP	Varianzen sind gleich	1,776	,190	,413	39
	Varianzen sind nicht gleich			,403	32,859

Test bei unabhängigen Stichproben

		t-Test für die Mittelwertgleichheit Sig. (2-seitig)	Mittlere Differenz	Differenz für Standardfehler
MBIpaersterZP	Varianzen sind gleich	,682	,96860	2,34783
	Varianzen sind nicht gleich	,689	,96860	2,40294

Test bei unabhängigen Stichproben

		t-Test für die Mittelwertgleichheit 95% Konfidenzintervall der Differenz Unterer Wert	Oberer Wert
MBIpaersterZP	Varianzen sind gleich	-3,78033	5,71753
	Varianzen sind nicht gleich	-3,92102	5,85822

Effektgrößen bei unabhängigen Stichproben

		Standardisierer[a]	Punktschätzung	95% Konfidenzintervall Unterer Wert	95% Konfidenzintervall Oberer Wert
MBIpaersterZP	Cohen's d	7,46062	,130	-,488	,746
	Hedges' Korrektur	7,60803	,127	-,479	,732
	Glass' Delta	8,22101	,118	-,502	,734

a. Der bei der Schätzung der Effektgrößen verwendete Nenner.
Cohen's d verwendet die zusammengefasste Standardabweichung.
Hedges' Korrektur verwendet die zusammengefasste Standardabweichung und einen Korrekturfaktor.
Glass' Delta verwendet die Standardabweichung einer Stichprobe von der Kontrollgruppe.

```
T-TEST GROUPS=STAITraitMedianaufteilungErsterZP(1 2)
  /MISSING=ANALYSIS
  /VARIABLES=MBIpazweiterZP
```

```
/ES DISPLAY(TRUE)
/CRITERIA=CI(.95).
```

t-Test

Gruppenstatistiken

	STAITraitMedianaufteilung ErsterZP	N	Mittelwert	Std.-Abweichung	Standardfehler des Mittelwertes
MBIpazweiterZP	1bis40	13	40,2308	6,05742	1,68003
	ab41	13	33,0769	12,65874	3,51090

Test bei unabhängigen Stichproben

		Levene-Test der Varianzgleichheit		t-Test für die Mittelwertgleichheit	
		F	Sig.	T	df
MBIpazweiterZP	Varianzen sind gleich	7,477	,012	1,838	24
	Varianzen sind nicht gleich			1,838	17,222

Test bei unabhängigen Stichproben

		t-Test für die Mittelwertgleichheit		
		Sig. (2-seitig)	Mittlere Differenz	Differenz für Standardfehler
MBIpazweiterZP	Varianzen sind gleich	,078	7,15385	3,89216
	Varianzen sind nicht gleich	,083	7,15385	3,89216

Test bei unabhängigen Stichproben

		t-Test für die Mittelwertgleichheit: 95% Konfidenzintervall der Differenz	
		Unterer Wert	Oberer Wert
MBIpazweiterZP	Varianzen sind gleich	-,87918	15,18687
	Varianzen sind nicht gleich	-1,04985	15,35754

Effektgrößen bei unabhängigen Stichproben

		Standardisierer[a]	Punktschätzung	95% Konfidenzintervall	
				Unterer Wert	Oberer Wert
MBIpazweiterZP	Cohen's d	9,92310	,721	-,081	1,509
	Hedges' Korrektur	10,24727	,698	-,079	1,461
	Glass' Delta	12,65874	,565	-,246	1,355

a. Der bei der Schätzung der Effektgrößen verwendete Nenner.
Cohen's d verwendet die zusammengefasste Standardabweichung.
Hedges' Korrektur verwendet die zusammengefasste Standardabweichung und einen Korrekturfaktor.
Glass' Delta verwendet die Standardabweichung einer Stichprobe von der Kontrollgruppe.

```
T-TEST GROUPS=STAITraitMedianaufteilungErsterZP(1 2)
  /MISSING=ANALYSIS
  /VARIABLES=WIersterZP
  /ES DISPLAY(TRUE)
  /CRITERIA=CI(.95).
```

t-Test

Gruppenstatistiken

	STAITraitMedianaufteilung ErsterZP	N	Mittelwert	Std.-Abweichung	Standardfehler des Mittelwertes
WIersterZP	1bis40	23	2,5652	1,97314	,41143
	ab41	21	4,4762	2,46209	,53727

Test bei unabhängigen Stichproben

		Levene-Test der Varianzgleichheit		t-Test für die Mittelwertgleichheit	
		F	Sig.	T	df
WIersterZP	Varianzen sind gleich	,591	,446	-2,853	42
	Varianzen sind nicht gleich			-2,824	38,346

Test bei unabhängigen Stichproben

		t-Test für die Mittelwertgleichheit		
		Sig. (2-seitig)	Mittlere Differenz	Differenz für Standardfehler
WIersterZP	Varianzen sind gleich	,007	-1,91097	,66988
	Varianzen sind nicht gleich	,007	-1,91097	,67671

Test bei unabhängigen Stichproben

		t-Test für die Mittelwertgleichheit	
		95% Konfidenzintervall der Differenz	
		Unterer Wert	Oberer Wert
WIersterZP	Varianzen sind gleich	-3,26285	-,55910
	Varianzen sind nicht gleich	-3,28049	-,54145

Effektgrößen bei unabhängigen Stichproben

		Standardisierer[a]	Punktschätzung	95% Konfidenzintervall	
				Unterer Wert	Oberer Wert
WIersterZP	Cohen's d	2,21945	-,861	-1,476	-,237
	Hedges' Korrektur	2,26009	-,846	-1,449	-,233
	Glass' Delta	2,46209	-,776	-1,406	-,130

a. Der bei der Schätzung der Effektgrößen verwendete Nenner.
Cohen's d verwendet die zusammengefasste Standardabweichung.
Hedges' Korrektur verwendet die zusammengefasste Standardabweichung und einen Korrekturfaktor.
Glass' Delta verwendet die Standardabweichung einer Stichprobe von der Kontrollgruppe.

```
T-TEST GROUPS=STAITraitMedianaufteilungErsterZP(1 2)
  /MISSING=ANALYSIS
  /VARIABLES=WIzweiterZP
  /ES DISPLAY(TRUE)
  /CRITERIA=CI(.95).
```

t-Test

Gruppenstatistiken

	STAITraitMedianaufteilung ErsterZP	N	Mittelwert	Std.-Abweichung	Standardfehler des Mittelwertes
WIzweiterZP	1bis40	13	1,9231	1,84669	,51218
	ab41	14	4,0714	2,61547	,69901

Test bei unabhängigen Stichproben

		Levene-Test der Varianzgleichheit		t-Test für die Mittelwertgleichheit	
		F	Sig.	T	df
WIzweiterZP	Varianzen sind gleich	1,058	,314	-2,447	25
	Varianzen sind nicht gleich			-2,479	23,399

Test bei unabhängigen Stichproben

		t-Test für die Mittelwertgleichheit		
		Sig. (2-seitig)	Mittlere Differenz	Differenz für Standardfehler
WIzweiterZP	Varianzen sind gleich	,022	-2,14835	,87781
	Varianzen sind nicht gleich	,021	-2,14835	,86657

Test bei unabhängigen Stichproben

		t-Test für die Mittelwertgleichheit	
		95% Konfidenzintervall der Differenz	
		Unterer Wert	Oberer Wert
WIzweiterZP	Varianzen sind gleich	-3,95623	-,34047
	Varianzen sind nicht gleich	-3,93930	-,35740

Effektgrößen bei unabhängigen Stichproben

		Standardisierer[a]	Punktschätzung	95% Konfidenzintervall	
				Unterer Wert	Oberer Wert
WIzweiterZP	Cohen's d	2,27905	-,943	-1,733	-,136
	Hedges' Korrektur	2,35039	-,914	-1,680	-,132
	Glass' Delta	2,61547	-,821	-1,625	,009

a. Der bei der Schätzung der Effektgrößen verwendete Nenner.
Cohen's d verwendet die zusammengefasste Standardabweichung.
Hedges' Korrektur verwendet die zusammengefasste Standardabweichung und einen Korrekturfaktor.
Glass' Delta verwendet die Standardabweichung einer Stichprobe von der Kontrollgruppe.

```
T-TEST GROUPS=STAITraitMedianaufteilungErsterZP(1 2)
  /MISSING=ANALYSIS
  /VARIABLES=BVIersterZP
```

```
/ES DISPLAY(TRUE)
/CRITERIA=CI(.95).
```

t-Test

Gruppenstatistiken

	STAITraitMedianaufteilung ErsterZP	N	Mittelwert	Std.-Abweichung	Standardfehler des Mittelwertes
BVIersterZP	1bis40	23	39,5626	6,43984	1,34280
	ab41	21	48,5381	6,11896	1,33527

Test bei unabhängigen Stichproben

		Levene-Test der Varianzgleichheit		t-Test für die Mittelwertgleichheit	
		F	Sig.	T	df
BVIersterZP	Varianzen sind gleich	,008	,927	-4,728	42
	Varianzen sind nicht gleich			-4,740	41,926

Test bei unabhängigen Stichproben

		t-Test für die Mittelwertgleichheit		
		Sig. (2-seitig)	Mittlere Differenz	Differenz für Standardfehler
BVIersterZP	Varianzen sind gleich	,000	-8,97549	1,89819
	Varianzen sind nicht gleich	,000	-8,97549	1,89369

Test bei unabhängigen Stichproben

		t-Test für die Mittelwertgleichheit	
		95% Konfidenzintervall der Differenz	
		Unterer Wert	Oberer Wert
BVIersterZP	Varianzen sind gleich	-12,80619	-5,14478
	Varianzen sind nicht gleich	-12,79730	-5,15367

Effektgrößen bei unabhängigen Stichproben

		Standardisierer[a]	Punktschätzung	95% Konfidenzintervall	
				Unterer Wert	Oberer Wert
BVIersterZP	Cohen's d	6,28908	-1,427	-2,086	-,755
	Hedges' Korrektur	6,40425	-1,401	-2,048	-,742
	Glass' Delta	6,11896	-1,467	-2,199	-,711

a. Der bei der Schätzung der Effektgrößen verwendete Nenner.
Cohen's d verwendet die zusammengefasste Standardabweichung.
Hedges' Korrektur verwendet die zusammengefasste Standardabweichung und einen Korrekturfaktor.
Glass' Delta verwendet die Standardabweichung einer Stichprobe von der Kontrollgruppe.

```
T-TEST GROUPS=STAITraitMedianaufteilungErsterZP(1 2)
  /MISSING=ANALYSIS
  /VARIABLES=BVIzweiterZP
  /ES DISPLAY(TRUE)
  /CRITERIA=CI(.95).
```

t-Test

Gruppenstatistiken

	STAITraitMedianaufteilung ErsterZP	N	Mittelwert	Std.-Abweichung	Standardfehler des Mittelwertes
BVIzweiterZP	1bis40	13	37,2477	6,30637	1,74907
	ab41	14	48,7336	8,79323	2,35009

Test bei unabhängigen Stichproben

		Levene-Test der Varianzgleichheit		t-Test für die Mittelwertgleichheit	
		F	Sig.	T	df
BVIzweiterZP	Varianzen sind gleich	,693	,413	-3,873	25
	Varianzen sind nicht gleich			-3,921	23,560

Test bei unabhängigen Stichproben

		t-Test für die Mittelwertgleichheit		
		Sig. (2-seitig)	Mittlere Differenz	Differenz für Standardfehler
BVIzweiterZP	Varianzen sind gleich	,001	-11,48588	2,96593
	Varianzen sind nicht gleich	,001	-11,48588	2,92954

Test bei unabhängigen Stichproben

		t-Test für die Mittelwertgleichheit	
		95% Konfidenzintervall der Differenz	
		Unterer Wert	Oberer Wert
BVIzweiterZP	Varianzen sind gleich	-17,59433	-5,37743
	Varianzen sind nicht gleich	-17,53813	-5,43363

Effektgrößen bei unabhängigen Stichproben

		Standardisierer[a]	Punktschätzung	95% Konfidenzintervall	
				Unterer Wert	Oberer Wert
BVIzweiterZP	Cohen's d	7,70043	-1,492	-2,340	-,620
	Hedges' Korrektur	7,94149	-1,446	-2,269	-,602
	Glass' Delta	8,79323	-1,306	-2,193	-,385

a. Der bei der Schätzung der Effektgrößen verwendete Nenner.
Cohen's d verwendet die zusammengefasste Standardabweichung.
Hedges' Korrektur verwendet die zusammengefasste Standardabweichung und einen Korrekturfaktor.
Glass' Delta verwendet die Standardabweichung einer Stichprobe von der Kontrollgruppe.

```
GLM STAIStateersterZP STAIStatezweiterZP BY STAITraitMedianaufteilungErsterZP
  /WSFACTOR=Faktor1 2 Polynomial
  /METHOD=SSTYPE(3)
  /CRITERIA=ALPHA(.05)
  /WSDESIGN=Faktor1
  /DESIGN=STAITraitMedianaufteilungErsterZP.
```

Allgemeines lineares Modell

Innersubjektfaktoren

Maß: MASS_1

Faktor1	Abhängige Variable
1	STAIStateersterZP
2	STAIStatezweiterZP

Zwischensubjektfaktoren

		Wertbeschriftung	N
STAITraitMedianaufteilung ErsterZP	1,00	1bis40	13
	2,00	ab41	14

Multivariate Tests[a]

Effekt		Wert	F	Hypothese df
Faktor1	Pillai-Spur	,099	2,755[b]	1,000
	Wilks-Lambda	,901	2,755[b]	1,000
	Hotelling-Spur	,110	2,755[b]	1,000
	Größte charakteristische Wurzel nach Roy	,110	2,755[b]	1,000
Faktor1 * STAITraitMedianaufteilung ErsterZP	Pillai-Spur	,015	,369[b]	1,000
	Wilks-Lambda	,985	,369[b]	1,000
	Hotelling-Spur	,015	,369[b]	1,000
	Größte charakteristische Wurzel nach Roy	,015	,369[b]	1,000

Multivariate Tests[a]

Effekt		Fehler df	Sig.
Faktor1	Pillai-Spur	25,000	,109
	Wilks-Lambda	25,000	,109
	Hotelling-Spur	25,000	,109
	Größte charakteristische Wurzel nach Roy	25,000	,109
Faktor1 * STAITraitMedianaufteilung ErsterZP	Pillai-Spur	25,000	,549
	Wilks-Lambda	25,000	,549
	Hotelling-Spur	25,000	,549
	Größte charakteristische Wurzel nach Roy	25,000	,549

a. Design: Konstanter Term + STAITraitMedianaufteilungErsterZP
Innersubjektdesign: Faktor1

b. Exakte Statistik

Mauchly-Test auf Sphärizität[a]

Maß: MASS_1

Innersubjekteffekt	Mauchly-W	Ungefähres Chi-Quadrat	df	Sig.	Epsilon[b] Greenhouse-Geisser
Faktor1	1,000	,000	0	.	1,000

Mauchly-Test auf Sphärizität[a]

Maß: MASS_1

	Epsilon[b]	
Innersubjekteffekt	Huynh-Feldt (HF)	Untergrenze
Faktor1	1,000	1,000

Prüft die Nullhypothese, dass sich die Fehlerkovarianz-Matrix der orthonormalisierten transformierten abhängigen Variablen proportional zur Einheitsmatrix verhält.

a. Design: Konstanter Term + STAITraitMedianaufteilungErsterZP
Innersubjektdesign: Faktor1

b. Kann zum Korrigieren der Freiheitsgrade für die gemittelten Signifikanztests verwendet werden. In der Tabelle mit den Tests der Effekte innerhalb der Subjekte werden korrigierte Tests angezeigt.

Tests der Innersubjekteffekte

Maß: MASS_1

Quelle		Typ III Quadratsumme	df	Mittel der Quadrate
Faktor1	Sphärizität angenommen	106,901	1	106,901
	Greenhouse-Geisser	106,901	1,000	106,901
	Huynh-Feldt (HF)	106,901	1,000	106,901
	Untergrenze	106,901	1,000	106,901
Faktor1 * STAITraitMedianaufteilung ErsterZP	Sphärizität angenommen	14,309	1	14,309
	Greenhouse-Geisser	14,309	1,000	14,309
	Huynh-Feldt (HF)	14,309	1,000	14,309
	Untergrenze	14,309	1,000	14,309
Fehler(Faktor1)	Sphärizität angenommen	970,025	25	38,801
	Greenhouse-Geisser	970,025	25,000	38,801
	Huynh-Feldt (HF)	970,025	25,000	38,801
	Untergrenze	970,025	25,000	38,801

Tests der Innersubjekteffekte

Maß: MASS_1

Quelle		F	Sig.
Faktor1	Sphärizität angenommen	2,755	,109
	Greenhouse-Geisser	2,755	,109
	Huynh-Feldt (HF)	2,755	,109
	Untergrenze	2,755	,109
Faktor1 * STAITraitMedianaufteilung ErsterZP	Sphärizität angenommen	,369	,549
	Greenhouse-Geisser	,369	,549
	Huynh-Feldt (HF)	,369	,549
	Untergrenze	,369	,549
Fehler(Faktor1)	Sphärizität angenommen		
	Greenhouse-Geisser		
	Huynh-Feldt (HF)		
	Untergrenze		

Tests der Innersubjektkontraste

Maß: MASS_1

Quelle	Faktor1	Typ III Quadratsumme	df	Mittel der Quadrate	F
Faktor1	Linear	106,901	1	106,901	2,755
Faktor1 * STAITraitMedianaufteilung ErsterZP	Linear	14,309	1	14,309	,369
Fehler(Faktor1)	Linear	970,025	25	38,801	

Tests der Innersubjektkontraste

Maß: MASS_1

Quelle	Faktor1	Sig.
Faktor1	Linear	,109
Faktor1 * STAITraitMedianaufteilung ErsterZP	Linear	,549
Fehler(Faktor1)	Linear	

Tests der Zwischensubjekteffekte

Maß: MASS_1

Transformierte Variable: Mittel

Quelle	Typ III Quadratsumme	df	Mittel der Quadrate	F	Sig.
Konstanter Term	128310,045	1	128310,045	628,935	,000
STAITraitMedianaufteilung ErsterZP	3010,045	1	3010,045	14,754	,001
Fehler	5100,288	25	204,012		

```
GLM STAIStateersterZP STAIStatezweiterZP BY STAITraitMedianaufteilungErsterZP
  /WSFACTOR=Faktor1 2 Polynomial
  /METHOD=SSTYPE(3)
  /PLOT=PROFILE(Faktor1*STAITraitMedianaufteilungErsterZP) TYPE=LINE ERRORBAR=NO MEANR
EFERENCE=NO
    YAXIS=AUTO
  /CRITERIA=ALPHA(.05)
  /WSDESIGN=Faktor1
  /DESIGN=STAITraitMedianaufteilungErsterZP.
```

Allgemeines lineares Modell

Innersubjektfaktoren

Maß: MASS_1

Faktor1	Abhängige Variable
1	STAIStateerstZP
2	STAIStatezweiterZP

Zwischensubjektfaktoren

		Wertbeschriftung	N
STAITraitMedianaufteilung ErsterZP	1,00	1bis40	13
	2,00	ab41	14

Multivariate Tests[a]

Effekt		Wert	F	Hypothese df
Faktor1	Pillai-Spur	,099	2,755[b]	1,000
	Wilks-Lambda	,901	2,755[b]	1,000
	Hotelling-Spur	,110	2,755[b]	1,000
	Größte charakteristische Wurzel nach Roy	,110	2,755[b]	1,000
Faktor1 * STAITraitMedianaufteilung ErsterZP	Pillai-Spur	,015	,369[b]	1,000
	Wilks-Lambda	,985	,369[b]	1,000
	Hotelling-Spur	,015	,369[b]	1,000
	Größte charakteristische Wurzel nach Roy	,015	,369[b]	1,000

Multivariate Tests[a]

Effekt		Fehler df	Sig.
Faktor1	Pillai-Spur	25,000	,109
	Wilks-Lambda	25,000	,109
	Hotelling-Spur	25,000	,109
	Größte charakteristische Wurzel nach Roy	25,000	,109
Faktor1 * STAITraitMedianaufteilung ErsterZP	Pillai-Spur	25,000	,549
	Wilks-Lambda	25,000	,549
	Hotelling-Spur	25,000	,549
	Größte charakteristische Wurzel nach Roy	25,000	,549

a. Design: Konstanter Term + STAITraitMedianaufteilungErsterZP
Innersubjektdesign: Faktor1

b. Exakte Statistik

Mauchly-Test auf Sphärizität[a]

Maß: MASS_1

Innersubjekteffekt	Mauchly-W	Ungefähres Chi-Quadrat	df	Sig.	Epsilon[b] Greenhouse-Geisser
Faktor1	1,000	,000	0	.	1,000

Mauchly-Test auf Sphärizität[a]

Maß: MASS_1

Innersubjekteffekt	Epsilon[b] Huynh-Feldt (HF)	Untergrenze
Faktor1	1,000	1,000

Prüft die Nullhypothese, dass sich die Fehlerkovarianz-Matrix der orthonormalisierten transformierten abhängigen Variablen proportional zur Einheitsmatrix verhält.

a. Design: Konstanter Term + STAITraitMedianaufteilungErsterZP
Innersubjektdesign: Faktor1

b. Kann zum Korrigieren der Freiheitsgrade für die gemittelten Signifikanztests verwendet werden. In der Tabelle mit den Tests der Effekte innerhalb der Subjekte werden korrigierte Tests angezeigt.

Tests der Innersubjekteffekte

Maß: MASS_1

Quelle		Typ III Quadratsumme	df	Mittel der Quadrate
Faktor1	Sphärizität angenommen	106,901	1	106,901
	Greenhouse-Geisser	106,901	1,000	106,901
	Huynh-Feldt (HF)	106,901	1,000	106,901
	Untergrenze	106,901	1,000	106,901
Faktor1 * STAITraitMedianaufteilung ErsterZP	Sphärizität angenommen	14,309	1	14,309
	Greenhouse-Geisser	14,309	1,000	14,309
	Huynh-Feldt (HF)	14,309	1,000	14,309
	Untergrenze	14,309	1,000	14,309
Fehler(Faktor1)	Sphärizität angenommen	970,025	25	38,801
	Greenhouse-Geisser	970,025	25,000	38,801
	Huynh-Feldt (HF)	970,025	25,000	38,801
	Untergrenze	970,025	25,000	38,801

Tests der Innersubjekteffekte

Maß: MASS_1

Quelle		F	Sig.
Faktor1	Sphärizität angenommen	2,755	,109
	Greenhouse-Geisser	2,755	,109
	Huynh-Feldt (HF)	2,755	,109
	Untergrenze	2,755	,109
Faktor1 * STAITraitMedianaufteilung ErsterZP	Sphärizität angenommen	,369	,549
	Greenhouse-Geisser	,369	,549
	Huynh-Feldt (HF)	,369	,549
	Untergrenze	,369	,549
Fehler(Faktor1)	Sphärizität angenommen		
	Greenhouse-Geisser		
	Huynh-Feldt (HF)		
	Untergrenze		

Tests der Innersubjektkontraste

Maß: MASS_1

Quelle	Faktor1	Typ III Quadratsumme	df	Mittel der Quadrate	F
Faktor1	Linear	106,901	1	106,901	2,755
Faktor1 * STAITraitMedianaufteilung ErsterZP	Linear	14,309	1	14,309	,369
Fehler(Faktor1)	Linear	970,025	25	38,801	

Tests der Innersubjektkontraste

Maß: MASS_1

Quelle	Faktor1	Sig.
Faktor1	Linear	,109
Faktor1 * STAITraitMedianaufteilung ErsterZP	Linear	,549
Fehler(Faktor1)	Linear	

Tests der Zwischensubjekteffekte

Maß: MASS_1

Transformierte Variable: Mittel

Quelle	Typ III Quadratsumme	df	Mittel der Quadrate	F	Sig.
Konstanter Term	128310,045	1	128310,045	628,935	,000
STAITraitMedianaufteilung ErsterZP	3010,045	1	3010,045	14,754	,001
Fehler	5100,288	25	204,012		

Profilplots

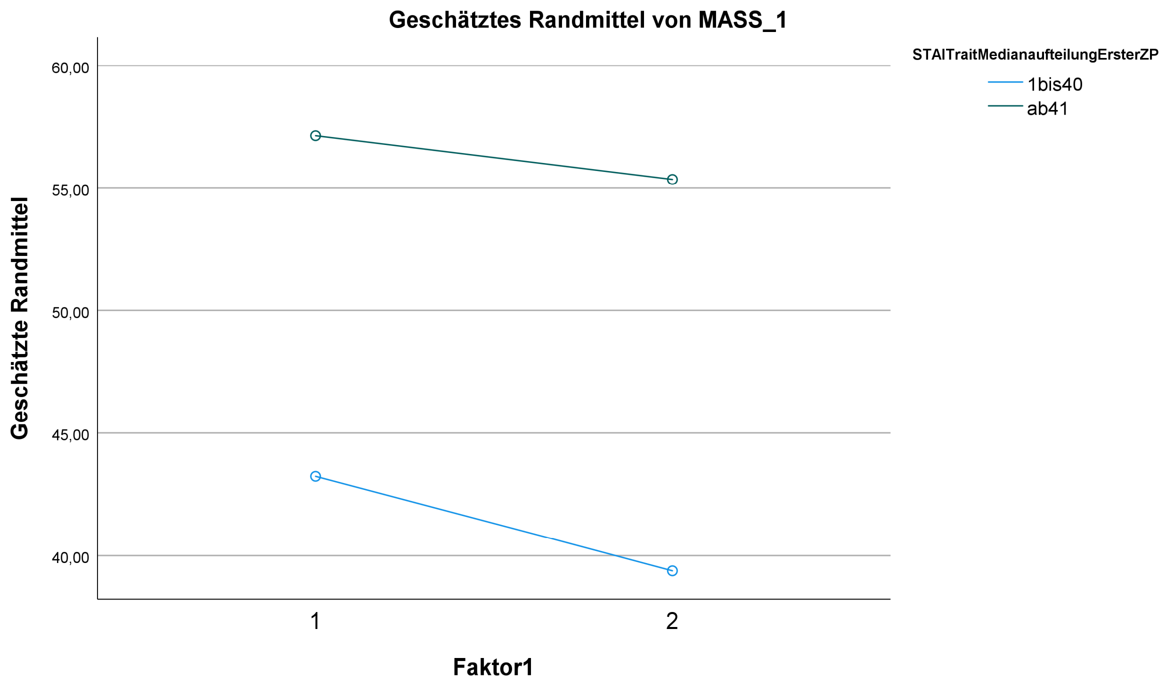


```
T-TEST PAIRS=STAIStateersterZP WITH STAIStatezweiterZP (PAIRED)
  /ES DISPLAY(TRUE) STANDARDIZER(SD)
  /CRITERIA=CI(.9500)
  /MISSING=ANALYSIS.
```

t-Test

Statistik bei gepaarten Stichproben

		Mittelwert	N	Std.-Abweichung	Standardfehler des Mittelwertes
Paaren 1	STAIStateersterZP	50,4444	27	11,74188	2,25973
	STAIStatezweiterZP	47,6667	27	14,55758	2,80161

Korrelationen bei gepaarten Stichproben

		N	Korrelation	Sig.
Paaren 1	STAIStateersterZP & STAIStatezweiterZP	27	,802	,000

Test bei gepaarten Stichproben

		Gepaarte Differenzen			
		Mittelwert	Std.-Abweichung	Standardfehler des Mittelwertes	95% Konfidenzinterv... Unterer Wert
Paaren 1	STAIStateersterZP - STAIStatezweiterZP	2,77778	8,70161	1,67462	-,66446

Test bei gepaarten Stichproben

		Gepaarte ... 95% Konfidenzinterval...			
		Oberer Wert	T	df	Sig. (2-seitig)
Paaren 1	STAIStateersterZP - STAIStatezweiterZP	6,22002	1,659	26	,109

Effektgrößen bei Stichproben mit paarigen Werten

			Standardisierer[a]	Punktschätzung
Paaren 1	STAIStateersterZP - STAIStatezweiterZP	Cohen's d	8,70161	,319
		Hedges' Korrektur	8,82968	,315

Effektgrößen bei Stichproben mit paarigen Werten

			95% Konfidenzintervall	
			Unterer Wert	Oberer Wert
Paaren 1	STAIStateersterZP - STAIStatezweiterZP	Cohen's d	-,071	,703
		Hedges' Korrektur	-,070	,693

a. Der bei der Schätzung der Effektgrößen verwendete Nenner.
Cohen's d verwendet die Standardabweichung einer Stichprobe der Mittelwertdifferenz.
Hedges' Korrektur verwendet die Standardabweichung einer Stichprobe der Mittelwertdifferenz und einen Korrekturfaktor.

```
T-TEST PAIRS=ISIersterZP WITH ISIzweiterZP (PAIRED)
  /ES DISPLAY(TRUE) STANDARDIZER(SD)
  /CRITERIA=CI(.9500)
  /MISSING=ANALYSIS.
```

t-Test

Statistik bei gepaarten Stichproben

		Mittelwert	N	Std.-Abweichung	Standardfehler des Mittelwertes
Paaren 1	ISIersterZP	10,3103	29	6,38695	1,18603
	ISIzweiterZP	10,0690	29	6,09995	1,13273

Korrelationen bei gepaarten Stichproben

		N	Korrelation	Sig.
Paaren 1	ISIersterZP & ISIzweiterZP	29	,669	,000

Test bei gepaarten Stichproben

		Gepaarte Differenzen			
		Mittelwert	Std.-Abweichung	Standardfehler des Mittelwertes	95% Konfidenzinterv... Unterer Wert
Paaren 1	ISIersterZP - ISIzweiterZP	,24138	5,08959	,94511	-1,69460

Test bei gepaarten Stichproben

		Gepaarte ... 95% Konfidenzinterval...			
		Oberer Wert	T	df	Sig. (2-seitig)
Paaren 1	ISIersterZP - ISIzweiterZP	2,17736	,255	28	,800

Effektgrößen bei Stichproben mit paarigen Werten

			Standardisierer[a]	Punktschätzung
Paaren 1	ISIersterZP - ISIzweiterZP	Cohen's d	5,08959	,047
		Hedges' Korrektur	5,15905	,047

Effektgrößen bei Stichproben mit paarigen Werten

			95% Konfidenzintervall	
			Unterer Wert	Oberer Wert
Paaren 1	ISIersterZP - ISIzweiterZP	Cohen's d	-,317	,411
		Hedges' Korrektur	-,313	,406

a. Der bei der Schätzung der Effektgrößen verwendete Nenner.
Cohen's d verwendet die Standardabweichung einer Stichprobe der Mittelwertdifferenz.
Hedges' Korrektur verwendet die Standardabweichung einer Stichprobe der Mittelwertdifferenz und einen Korrekturfaktor.

```
T-TEST PAIRS=ESSersterZP WITH ESSzweiterZP (PAIRED)
  /ES DISPLAY(TRUE) STANDARDIZER(SD)
  /CRITERIA=CI(.9500)
  /MISSING=ANALYSIS.
```

t-Test

Statistik bei gepaarten Stichproben

		Mittelwert	N	Std.-Abweichung	Standardfehler des Mittelwertes
Paaren 1	ESSersterZP	10,7143	28	4,24139	,80155
	ESSzweiterZP	10,8571	28	4,86647	,91968

Korrelationen bei gepaarten Stichproben

		N	Korrelation	Sig.
Paaren 1	ESSersterZP & ESSzweiterZP	28	,701	,000

Test bei gepaarten Stichproben

		Gepaarte Differenzen			
					95% Konfidenzinterv...
		Mittelwert	Std.-Abweichung	Standardfehler des Mittelwertes	Unterer Wert
Paaren 1	ESSersterZP - ESSzweiterZP	-,14286	3,56645	,67400	-1,52578

Test bei gepaarten Stichproben

		Gepaarte ...			
		95% Konfidenzinterval...			
		Oberer Wert	T	df	Sig. (2-seitig)
Paaren 1	ESSersterZP - ESSzweiterZP	1,24007	-,212	27	,834

Effektgrößen bei Stichproben mit paarigen Werten

			Standardisierer[a]	Punktschätzung
Paaren 1	ESSersterZP - ESSzweiterZP	Cohen's d	3,56645	-,040
		Hedges' Korrektur	3,61696	-,039

Effektgrößen bei Stichproben mit paarigen Werten

			95% Konfidenzintervall	
			Unterer Wert	Oberer Wert
Paaren 1	ESSersterZP - ESSzweiterZP	Cohen's d	-,410	,331
		Hedges' Korrektur	-,405	,326

a. Der bei der Schätzung der Effektgrößen verwendete Nenner.
Cohen's d verwendet die Standardabweichung einer Stichprobe der Mittelwertdifferenz.
Hedges' Korrektur verwendet die Standardabweichung einer Stichprobe der Mittelwertdifferenz und einen Korrekturfaktor.

```
T-TEST PAIRS=PSSersterZP WITH PSSzweiterZP (PAIRED)
  /ES DISPLAY(TRUE) STANDARDIZER(SD)
  /CRITERIA=CI(.9500)
  /MISSING=ANALYSIS.
```

t-Test

Statistik bei gepaarten Stichproben

		Mittelwert	N	Std.-Abweichung	Standardfehler des Mittelwertes
Paaren 1	PSSersterZP	20,6207	29	6,33253	1,17592
	PSSzweiterZP	19,9310	29	6,68116	1,24066

Korrelationen bei gepaarten Stichproben

		N	Korrelation	Sig.
Paaren 1	PSSersterZP & PSSzweiterZP	29	,867	,000

Test bei gepaarten Stichproben

		Gepaarte Differenzen			
					95% Konfidenzinterv...
		Mittelwert	Std.-Abweichung	Standardfehler des Mittelwertes	Unterer Wert
Paaren 1	PSSersterZP - PSSzweiterZP	,68966	3,37113	,62600	-,59265

Test bei gepaarten Stichproben

		Gepaarte ...			
		95% Konfidenzinterval...			
		Oberer Wert	T	df	Sig. (2-seitig)
Paaren 1	PSSersterZP - PSSzweiterZP	1,97197	1,102	28	,280

Effektgrößen bei Stichproben mit paarigen Werten

			Standardisierer[a]	Punktschätzung
Paaren 1	PSSersterZP - PSSzweiterZP	Cohen's d	3,37113	,205
		Hedges' Korrektur	3,41714	,202

Effektgrößen bei Stichproben mit paarigen Werten

			95% Konfidenzintervall	
			Unterer Wert	Oberer Wert
Paaren 1	PSSersterZP - PSSzweiterZP	Cohen's d	-,165	,571
		Hedges' Korrektur	-,163	,563

a. Der bei der Schätzung der Effektgrößen verwendete Nenner.
Cohen's d verwendet die Standardabweichung einer Stichprobe der Mittelwertdifferenz.
Hedges' Korrektur verwendet die Standardabweichung einer Stichprobe der Mittelwertdifferenz und einen Korrekturfaktor.

```
T-TEST PAIRS=GBBGesamtskalaBersterZP WITH GBBGesamtskalaBzweiterZP (PAIRED)
  /ES DISPLAY(TRUE) STANDARDIZER(SD)
  /CRITERIA=CI(.9500)
  /MISSING=ANALYSIS.
```

t-Test

Statistik bei gepaarten Stichproben

		Mittelwert	N	Std.-Abweichung	Standardfehler des Mittelwertes
Paaren 1	GBBGesamtskalaBersterZP	21,7500	28	12,14000	2,29424
	GBBGesamtskalaBzweiterZP	22,7500	28	12,06963	2,28095

Korrelationen bei gepaarten Stichproben

		N	Korrelation	Sig.
Paaren 1	GBBGesamtskalaBersterZP & GBBGesamtskalaBzweiterZP	28	,527	,004

Test bei gepaarten Stichproben

		Gepaarte Differenzen			
					95% Konfidenzinterv...
		Mittelwert	Std.-Abweichung	Standardfehler des Mittelwertes	Unterer Wert
Paaren 1	GBBGesamtskalaBersterZP - GBBGesamtskalaBzweiterZP	-1,00000	11,77254	2,22480	-5,56491

Test bei gepaarten Stichproben

		Gepaarte ...			
		95% Konfidenzinterval...			
		Oberer Wert	T	df	Sig. (2-seitig)
Paaren 1	GBBGesamtskalaBersterZP - GBBGesamtskalaBzweiterZP	3,56491	-,449	27	,657

Effektgrößen bei Stichproben mit paarigen Werten

			Standardisierer[a]	Punktschätzung
Paaren 1	GBBGesamtskalaBersterZP - GBBGesamtskalaBzweiterZP	Cohen's d	11,77254	-,085
		Hedges' Korrektur	11,93926	-,084

Effektgrößen bei Stichproben mit paarigen Werten

			95% Konfidenzintervall	
			Unterer Wert	Oberer Wert
Paaren 1	GBBGesamtskalaBersterZP - GBBGesamtskalaBzweiterZP	Cohen's d	-,455	,287
		Hedges' Korrektur	-,449	,283

a. Der bei der Schätzung der Effektgrößen verwendete Nenner.
Cohen's d verwendet die Standardabweichung einer Stichprobe der Mittelwertdifferenz.
Hedges' Korrektur verwendet die Standardabweichung einer Stichprobe der Mittelwertdifferenz und einen Korrekturfaktor.

```
T-TEST PAIRS=TICSersterZPsscs WITH TICSzweiterZPsscs (PAIRED)
  /ES DISPLAY(TRUE) STANDARDIZER(SD)
  /CRITERIA=CI(.9500)
  /MISSING=ANALYSIS.
```

t-Test

Statistik bei gepaarten Stichproben

		Mittelwert	N	Std.-Abweichung	Standardfehler des Mittelwertes
Paaren 1	TICSersterZPsscs	19,2143	28	8,20279	1,55018
	TICSzweiterZPsscs	17,2857	28	9,78986	1,85011

Korrelationen bei gepaarten Stichproben

		N	Korrelation	Sig.
Paaren 1	TICSersterZPsscs & TICSzweiterZPsscs	28	-,228	,244

Test bei gepaarten Stichproben

		Gepaarte Differenzen			
		Mittelwert	Std.-Abweichung	Standardfehler des Mittelwertes	95% Konfidenzinterv... Unterer Wert
Paaren 1	TICSersterZPsscs - TICSzweiterZPsscs	1,92857	14,13147	2,67060	-3,55104

Test bei gepaarten Stichproben

		Gepaarte ... 95% Konfidenzinterval...			
		Oberer Wert	T	df	Sig. (2-seitig)
Paaren 1	TICSersterZPsscs - TICSzweiterZPsscs	7,40818	,722	27	,476

Effektgrößen bei Stichproben mit paarigen Werten

			Standardisierer[a]	Punktschätzung
Paaren 1	TICSersterZPsscs - TICSzweiterZPsscs	Cohen's d	14,13147	,136
		Hedges' Korrektur	14,33160	,135

Effektgrößen bei Stichproben mit paarigen Werten

			95% Konfidenzintervall	
			Unterer Wert	Oberer Wert
Paaren 1	TICSersterZPsscs - TICSzweiterZPsscs	Cohen's d	-,237	,507
		Hedges' Korrektur	-,234	,500

a. Der bei der Schätzung der Effektgrößen verwendete Nenner.
Cohen's d verwendet die Standardabweichung einer Stichprobe der Mittelwertdifferenz.
Hedges' Korrektur verwendet die Standardabweichung einer Stichprobe der Mittelwertdifferenz und einen Korrekturfaktor.

```
T-TEST PAIRS=MBIeeersterZP WITH MBIeezweiterZP (PAIRED)
  /ES DISPLAY(TRUE) STANDARDIZER(SD)
  /CRITERIA=CI(.9500)
  /MISSING=ANALYSIS.
```

t-Test

Statistik bei gepaarten Stichproben

		Mittelwert	N	Std.-Abweichung	Standardfehler des Mittelwertes
Paaren 1	MBIeeersterZP	18,8846	26	12,07419	2,36794
	MBIeezweiterZP	18,3077	26	12,93296	2,53636

Korrelationen bei gepaarten Stichproben

		N	Korrelation	Sig.
Paaren 1	MBIeeersterZP & MBIeezweiterZP	26	,686	,000

Test bei gepaarten Stichproben

		Gepaarte Differenzen			
		Mittelwert	Std.-Abweichung	Standardfehler des Mittelwertes	95% Konfidenzinterv... Unterer Wert
Paaren 1	MBIeeersterZP - MBIeezweiterZP	,57692	9,93246	1,94792	-3,43489

Test bei gepaarten Stichproben

		Gepaarte ... 95% Konfidenzinterval...			
		Oberer Wert	T	df	Sig. (2-seitig)
Paaren 1	MBIeeersterZP - MBIeezweiterZP	4,58873	,296	25	,770

Effektgrößen bei Stichproben mit paarigen Werten

			Standardisierer[a]	Punktschätzung
Paaren 1	MBIeeersterZP - MBIeezweiterZP	Cohen's d	9,93246	,058
		Hedges' Korrektur	10,08462	,057

Effektgrößen bei Stichproben mit paarigen Werten

			95% Konfidenzintervall	
			Unterer Wert	Oberer Wert
Paaren 1	MBleeersterZP - MBleezweiterZP	Cohen's d	-,327	,442
		Hedges' Korrektur	-,322	,436

a. Der bei der Schätzung der Effektgrößen verwendete Nenner.
Cohen's d verwendet die Standardabweichung einer Stichprobe der Mittelwertdifferenz.
Hedges' Korrektur verwendet die Standardabweichung einer Stichprobe der Mittelwertdifferenz und einen Korrekturfaktor.

```
T-TEST PAIRS=MBIdersterZP WITH MBIdzweiterZP (PAIRED)
  /ES DISPLAY(TRUE) STANDARDIZER(SD)
  /CRITERIA=CI(.9500)
  /MISSING=ANALYSIS.
```

t-Test

Statistik bei gepaarten Stichproben

		Mittelwert	N	Std.-Abweichung	Standardfehler des Mittelwertes
Paaren 1	MBIdersterZP	3,1154	26	3,53640	,69355
	MBIdzweiterZP	3,6923	26	3,65261	,71634

Korrelationen bei gepaarten Stichproben

		N	Korrelation	Sig.
Paaren 1	MBIdersterZP & MBIdzweiterZP	26	,684	,000

Test bei gepaarten Stichproben

		Gepaarte Differenzen			
		Mittelwert	Std.-Abweichung	Standardfehler des Mittelwertes	95% Konfidenzinterv... Unterer Wert
Paaren 1	MBIdersterZP - MBIdzweiterZP	-,57692	2,85899	,56069	-1,73170

Test bei gepaarten Stichproben

		Gepaarte ... 95% Konfidenzinterval...			
		Oberer Wert	T	df	Sig. (2-seitig)
Paaren 1	MBIdersterZP - MBIdzweiterZP	,57785	-1,029	25	,313

Effektgrößen bei Stichproben mit paarigen Werten

			Standardisierer[a]	Punktschätzung
Paaren 1	MBIdersterZP - MBIdzweiterZP	Cohen's d	2,85899	-,202
		Hedges' Korrektur	2,90279	-,199

Effektgrößen bei Stichproben mit paarigen Werten

			95% Konfidenzintervall	
			Unterer Wert	Oberer Wert
Paaren 1	MBIdersterZP - MBIdzweiterZP	Cohen's d	-,588	,189
		Hedges' Korrektur	-,579	,186

a. Der bei der Schätzung der Effektgrößen verwendete Nenner.
Cohen's d verwendet die Standardabweichung einer Stichprobe der Mittelwertdifferenz.
Hedges' Korrektur verwendet die Standardabweichung einer Stichprobe der Mittelwertdifferenz und einen Korrekturfaktor.

```
T-TEST PAIRS=MBIpaersterZP WITH MBIpazweiterZP (PAIRED)
  /ES DISPLAY(TRUE) STANDARDIZER(SD)
  /CRITERIA=CI(.9500)
  /MISSING=ANALYSIS.
```

t-Test

Statistik bei gepaarten Stichproben

		Mittelwert	N	Std.-Abweichung	Standardfehler des Mittelwertes
Paaren 1	MBIpaersterZP	35,5000	26	10,81573	2,12114
	MBIpazweiterZP	35,8462	26	11,73266	2,30096

Korrelationen bei gepaarten Stichproben

		N	Korrelation	Sig.
Paaren 1	MBIpaersterZP & MBIpazweiterZP	26	,750	,000

Test bei gepaarten Stichproben

		Gepaarte Differenzen			
		Mittelwert	Std.-Abweichung	Standardfehler des Mittelwertes	95% Konfidenzinterv... Unterer Wert
Paaren 1	MBIpaersterZP - MBIpazweiterZP	-,34615	8,02467	1,57377	-3,58739

Test bei gepaarten Stichproben

		Gepaarte ... 95% Konfidenzinterval... Oberer Wert	T	df	Sig. (2-seitig)
Paaren 1	MBIpaersterZP - MBIpazweiterZP	2,89508	-,220	25	,828

Effektgrößen bei Stichproben mit paarigen Werten

			Standardisierer[a]	Punktschätzung
Paaren 1	MBIpaersterZP - MBIpazweiterZP	Cohen's d	8,02467	-,043
		Hedges' Korrektur	8,14761	-,042

Effektgrößen bei Stichproben mit paarigen Werten

			95% Konfidenzintervall Unterer Wert	95% Konfidenzintervall Oberer Wert
Paaren 1	MBIpaersterZP - MBIpazweiterZP	Cohen's d	-,427	,342
		Hedges' Korrektur	-,421	,337

a. Der bei der Schätzung der Effektgrößen verwendete Nenner.
Cohen's d verwendet die Standardabweichung einer Stichprobe der Mittelwertdifferenz.
Hedges' Korrektur verwendet die Standardabweichung einer Stichprobe der Mittelwertdifferenz und einen Korrekturfaktor.

```
T-TEST PAIRS=WIersterZP WITH WIzweiterZP (PAIRED)
  /ES DISPLAY(TRUE) STANDARDIZER(SD)
  /CRITERIA=CI(.9500)
  /MISSING=ANALYSIS.
```

t-Test

Statistik bei gepaarten Stichproben

		Mittelwert	N	Std.-Abweichung	Standardfehler des Mittelwertes
Paaren 1	WIersterZP	3,6786	28	2,58276	,48810
	WIzweiterZP	3,1071	28	2,46966	,46672

Korrelationen bei gepaarten Stichproben

		N	Korrelation	Sig.
Paaren 1	WIersterZP & WIzweiterZP	28	,836	,000

Test bei gepaarten Stichproben

		Gepaarte Differenzen			
		Mittelwert	Std.-Abweichung	Standardfehler des Mittelwertes	95% Konfidenzinterv... Unterer Wert
Paaren 1	WIersterZP - WIzweiterZP	,57143	1,45114	,27424	,00873

Test bei gepaarten Stichproben

		Gepaarte ... 95% Konfidenzinterval... Oberer Wert	T	df	Sig. (2-seitig)
Paaren 1	WIersterZP - WIzweiterZP	1,13412	2,084	27	,047

Effektgrößen bei Stichproben mit paarigen Werten

			Standardisierer[a]	Punktschätzung
Paaren 1	WIersterZP - WIzweiterZP	Cohen's d	1,45114	,394
		Hedges' Korrektur	1,47170	,388

Effektgrößen bei Stichproben mit paarigen Werten

			95% Konfidenzintervall	
			Unterer Wert	Oberer Wert
Paaren 1	WIersterZP - WIzweiterZP	Cohen's d	,006	,775
		Hedges' Korrektur	,005	,764

a. Der bei der Schätzung der Effektgrößen verwendete Nenner.
Cohen's d verwendet die Standardabweichung einer Stichprobe der Mittelwertdifferenz.
Hedges' Korrektur verwendet die Standardabweichung einer Stichprobe der Mittelwertdifferenz und einen Korrekturfaktor.

```
T-TEST PAIRS=BVIersterZP WITH BVIzweiterZP (PAIRED)
  /ES DISPLAY(TRUE) STANDARDIZER(SD)
  /CRITERIA=CI(.9500)
  /MISSING=ANALYSIS.
```

t-Test

Statistik bei gepaarten Stichproben

		Mittelwert	N	Std.-Abweichung	Standardfehler des Mittelwertes
Paaren 1	BVIersterZP	44,7118	28	8,19462	1,54864
	BVIzweiterZP	43,2396	28	9,37429	1,77157

Korrelationen bei gepaarten Stichproben

		N	Korrelation	Sig.
Paaren 1	BVIersterZP & BVIzweiterZP	28	,877	,000

Test bei gepaarten Stichproben

		Gepaarte Differenzen			
		Mittelwert	Std.-Abweichung	Standardfehler des Mittelwertes	95% Konfidenzinterv... Unterer Wert
Paaren 1	BVIersterZP - BVIzweiterZP	1,47214	4,51206	,85270	-,27745

Test bei gepaarten Stichproben

		Gepaarte ... 95% Konfidenzinterval...			
		Oberer Wert	T	df	Sig. (2-seitig)
Paaren 1	BVIersterZP - BVIzweiterZP	3,22174	1,726	27	,096

Effektgrößen bei Stichproben mit paarigen Werten

			Standardisierer[a]	Punktschätzung
Paaren 1	BVIersterZP - BVIzweiterZP	Cohen's d	4,51206	,326
		Hedges' Korrektur	4,57596	,322

Effektgrößen bei Stichproben mit paarigen Werten

			95% Konfidenzintervall	
			Unterer Wert	Oberer Wert
Paaren 1	BVIersterZP - BVIzweiterZP	Cohen's d	-,057	,704
		Hedges' Korrektur	-,056	,694

a. Der bei der Schätzung der Effektgrößen verwendete Nenner.
Cohen's d verwendet die Standardabweichung einer Stichprobe der Mittelwertdifferenz.
Hedges' Korrektur verwendet die Standardabweichung einer Stichprobe der Mittelwertdifferenz und einen Korrekturfaktor.

```
GLM FEELEadaptiveStrategienersterZP FEELEmaladaptiveStrategienersterZP BY
    STAITraitMedianaufteilungErsterZP
  /WSFACTOR=Faktor1 2 Polynomial
  /METHOD=SSTYPE(3)
  /PLOT=PROFILE(Faktor1*STAITraitMedianaufteilungErsterZP) TYPE=LINE ERRORBAR=NO MEANR
EFERENCE=NO
    YAXIS=AUTO
```

```
  /CRITERIA=ALPHA(.05)
  /WSDESIGN=Faktor1
  /DESIGN=STAITraitMedianaufteilungErsterZP.

GLM ISIersterZP ISIzweiterZP BY STAITraitMedianaufteilungErsterZP
  /WSFACTOR=Faktor1 2 Polynomial
  /METHOD=SSTYPE(3)
  /PLOT=PROFILE(Faktor1*STAITraitMedianaufteilungErsterZP) TYPE=LINE ERRORBAR=NO MEANR
EFERENCE=NO
    YAXIS=AUTO
  /CRITERIA=ALPHA(.05)
  /WSDESIGN=Faktor1
  /DESIGN=STAITraitMedianaufteilungErsterZP.
```

Allgemeines lineares Modell

Innersubjektfaktoren

Maß: MASS_1

Faktor1	Abhängige Variable
1	ISIersterZP
2	ISIzweiterZP

Zwischensubjektfaktoren

		Wertbeschriftung	N
STAITraitMedianaufteilung ErsterZP	1,00	1bis40	13
	2,00	ab41	14

Multivariate Tests[a]

Effekt		Wert	F	Hypothese df
Faktor1	Pillai-Spur	,007	,168[b]	1,000
	Wilks-Lambda	,993	,168[b]	1,000
	Hotelling-Spur	,007	,168[b]	1,000
	Größte charakteristische Wurzel nach Roy	,007	,168[b]	1,000
Faktor1 * STAITraitMedianaufteilung ErsterZP	Pillai-Spur	,024	,623[b]	1,000
	Wilks-Lambda	,976	,623[b]	1,000
	Hotelling-Spur	,025	,623[b]	1,000
	Größte charakteristische Wurzel nach Roy	,025	,623[b]	1,000

Multivariate Tests[a]

Effekt		Fehler df	Sig.
Faktor1	Pillai-Spur	25,000	,686
	Wilks-Lambda	25,000	,686
	Hotelling-Spur	25,000	,686
	Größte charakteristische Wurzel nach Roy	25,000	,686
Faktor1 * STAITraitMedianaufteilung ErsterZP	Pillai-Spur	25,000	,437
	Wilks-Lambda	25,000	,437
	Hotelling-Spur	25,000	,437
	Größte charakteristische Wurzel nach Roy	25,000	,437

a. Design: Konstanter Term + STAITraitMedianaufteilungErsterZP
Innersubjektdesign: Faktor1

b. Exakte Statistik

Mauchly-Test auf Sphärizität[a]

Maß: MASS_1

Innersubjekteffekt	Mauchly-W	Ungefähres Chi-Quadrat	df	Sig.	Epsilon[b] Greenhouse-Geisser
Faktor1	1,000	,000	0	.	1,000

Mauchly-Test auf Sphärizität[a]

Maß: MASS_1

Innersubjekteffekt	Epsilon[b] Huynh-Feldt (HF)	Untergrenze
Faktor1	1,000	1,000

Prüft die Nullhypothese, dass sich die Fehlerkovarianz-Matrix der orthonormalisierten transformierten abhängigen Variablen proportional zur Einheitsmatrix verhält.

a. Design: Konstanter Term + STAITraitMedianaufteilungErsterZP
Innersubjektdesign: Faktor1

b. Kann zum Korrigieren der Freiheitsgrade für die gemittelten Signifikanztests verwendet werden. In der Tabelle mit den Tests der Effekte innerhalb der Subjekte werden korrigierte Tests angezeigt.

Tests der Innersubjekteffekte

Maß: MASS_1

Quelle		Typ III Quadratsumme	df	Mittel der Quadrate
Faktor1	Sphärizität angenommen	2,320	1	2,320
	Greenhouse-Geisser	2,320	1,000	2,320
	Huynh-Feldt (HF)	2,320	1,000	2,320
	Untergrenze	2,320	1,000	2,320
Faktor1 * STAITraitMedianaufteilung ErsterZP	Sphärizität angenommen	8,616	1	8,616
	Greenhouse-Geisser	8,616	1,000	8,616
	Huynh-Feldt (HF)	8,616	1,000	8,616
	Untergrenze	8,616	1,000	8,616
Fehler(Faktor1)	Sphärizität angenommen	345,717	25	13,829
	Greenhouse-Geisser	345,717	25,000	13,829
	Huynh-Feldt (HF)	345,717	25,000	13,829
	Untergrenze	345,717	25,000	13,829

Tests der Innersubjekteffekte

Maß: MASS_1

Quelle		F	Sig.
Faktor1	Sphärizität angenommen	,168	,686
	Greenhouse-Geisser	,168	,686
	Huynh-Feldt (HF)	,168	,686
	Untergrenze	,168	,686
Faktor1 * STAITraitMedianaufteilung ErsterZP	Sphärizität angenommen	,623	,437
	Greenhouse-Geisser	,623	,437
	Huynh-Feldt (HF)	,623	,437
	Untergrenze	,623	,437
Fehler(Faktor1)	Sphärizität angenommen		
	Greenhouse-Geisser		
	Huynh-Feldt (HF)		
	Untergrenze		

Tests der Innersubjektkontraste

Maß: MASS_1

Quelle	Faktor1	Typ III Quadratsumme	df	Mittel der Quadrate	F
Faktor1	Linear	2,320	1	2,320	,168
Faktor1 * STAITraitMedianaufteilung ErsterZP	Linear	8,616	1	8,616	,623
Fehler(Faktor1)	Linear	345,717	25	13,829	

Tests der Innersubjektkontraste

Maß: MASS_1

Quelle	Faktor1	Sig.
Faktor1	Linear	,686
Faktor1 * STAITraitMedianaufteilung ErsterZP	Linear	,437
Fehler(Faktor1)	Linear	

Tests der Zwischensubjekteffekte

Maß: MASS_1

Transformierte Variable: Mittel

Quelle	Typ III Quadratsumme	df	Mittel der Quadrate	F	Sig.
Konstanter Term	6198,415	1	6198,415	132,412	,000
STAITraitMedianaufteilung ErsterZP	344,860	1	344,860	7,367	,012
Fehler	1170,288	25	46,812		

Profilplots

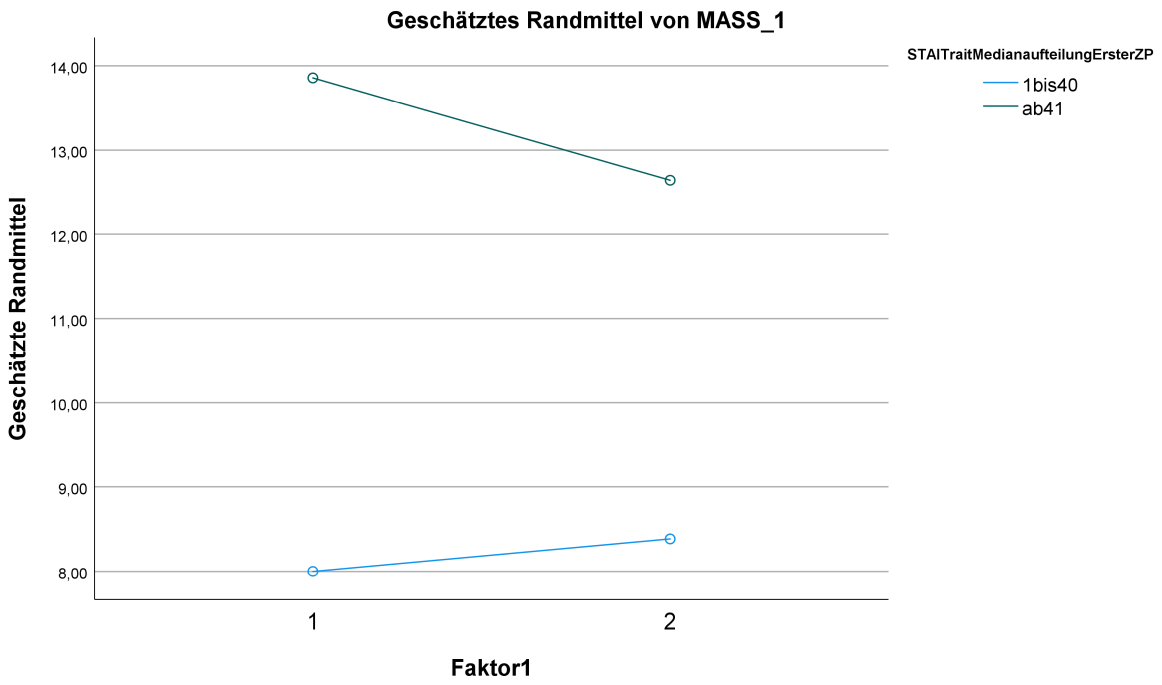


```
GLM ESSersterZP ESSzweiterZP BY STAITraitMedianaufteilungErsterZP
  /WSFACTOR=Faktor1 2 Polynomial
  /METHOD=SSTYPE(3)
  /PLOT=PROFILE(Faktor1*STAITraitMedianaufteilungErsterZP) TYPE=LINE ERRORBAR=NO MEA
EFERENCE=NO
    YAXIS=AUTO
  /CRITERIA=ALPHA(.05)
  /WSDESIGN=Faktor1
  /DESIGN=STAITraitMedianaufteilungErsterZP.
```

Allgemeines lineares Modell

Innersubjektfaktoren

Maß: MASS_1

Faktor1	Abhängige Variable
1	ESSersterZP
2	ESSzweiterZP

Zwischensubjektfaktoren

		Wertbeschriftung	N
STAITraitMedianaufteilungErsterZP	1,00	1bis40	13
	2,00	ab41	14

Multivariate Tests[a]

Effekt		Wert	F	Hypothese df
Faktor1	Pillai-Spur	,000	,000[b]	1,000
	Wilks-Lambda	1,000	,000[b]	1,000
	Hotelling-Spur	,000	,000[b]	1,000
	Größte charakteristische Wurzel nach Roy	,000	,000[b]	1,000
Faktor1 * STAITraitMedianaufteilung ErsterZP	Pillai-Spur	,011	,286[b]	1,000
	Wilks-Lambda	,989	,286[b]	1,000
	Hotelling-Spur	,011	,286[b]	1,000
	Größte charakteristische Wurzel nach Roy	,011	,286[b]	1,000

Multivariate Tests[a]

Effekt		Fehler df	Sig.
Faktor1	Pillai-Spur	25,000	,984
	Wilks-Lambda	25,000	,984
	Hotelling-Spur	25,000	,984
	Größte charakteristische Wurzel nach Roy	25,000	,984
Faktor1 * STAITraitMedianaufteilung ErsterZP	Pillai-Spur	25,000	,598
	Wilks-Lambda	25,000	,598
	Hotelling-Spur	25,000	,598
	Größte charakteristische Wurzel nach Roy	25,000	,598

a. Design: Konstanter Term + STAITraitMedianaufteilungErsterZP
Innersubjektdesign: Faktor1

b. Exakte Statistik

Mauchly-Test auf Sphärizität[a]

Maß: MASS_1

Innersubjekteffekt	Mauchly-W	Ungefähres Chi-Quadrat	df	Sig.	Epsilon[b] Greenhouse-Geisser
Faktor1	1,000	,000	0	.	1,000

Mauchly-Test auf Sphärizität[a]

Maß: MASS_1

Innersubjekteffekt	Epsilon[b] Huynh-Feldt (HF)	Untergrenze
Faktor1	1,000	1,000

Prüft die Nullhypothese, dass sich die Fehlerkovarianz-Matrix der orthonormalisierten transformierten abhängigen Variablen proportional zur Einheitsmatrix verhält.

a. Design: Konstanter Term + STAITraitMedianaufteilungErsterZP
Innersubjektdesign: Faktor1

b. Kann zum Korrigieren der Freiheitsgrade für die gemittelten Signifikanztests verwendet werden. In der Tabelle mit den Tests der Effekte innerhalb der Subjekte werden korrigierte Tests angezeigt.

Tests der Innersubjekteffekte

Maß: MASS_1

Quelle		Typ III Quadratsumme	df	Mittel der Quadrate
Faktor1	Sphärizität angenommen	,003	1	,003
	Greenhouse-Geisser	,003	1,000	,003
	Huynh-Feldt (HF)	,003	1,000	,003
	Untergrenze	,003	1,000	,003
Faktor1 * STAITraitMedianaufteilung ErsterZP	Sphärizität angenommen	1,854	1	1,854
	Greenhouse-Geisser	1,854	1,000	1,854
	Huynh-Feldt (HF)	1,854	1,000	1,854
	Untergrenze	1,854	1,000	1,854
Fehler(Faktor1)	Sphärizität angenommen	162,146	25	6,486
	Greenhouse-Geisser	162,146	25,000	6,486
	Huynh-Feldt (HF)	162,146	25,000	6,486
	Untergrenze	162,146	25,000	6,486

Tests der Innersubjekteffekte

Maß: MASS_1

Quelle		F	Sig.
Faktor1	Sphärizität angenommen	,000	,984
	Greenhouse-Geisser	,000	,984
	Huynh-Feldt (HF)	,000	,984
	Untergrenze	,000	,984
Faktor1 * STAITraitMedianaufteilungErsterZP	Sphärizität angenommen	,286	,598
	Greenhouse-Geisser	,286	,598
	Huynh-Feldt (HF)	,286	,598
	Untergrenze	,286	,598
Fehler(Faktor1)	Sphärizität angenommen		
	Greenhouse-Geisser		
	Huynh-Feldt (HF)		
	Untergrenze		

Tests der Innersubjektkontraste

Maß: MASS_1

Quelle	Faktor1	Typ III Quadratsumme	df	Mittel der Quadrate	F
Faktor1	Linear	,003	1	,003	,000
Faktor1 * STAITraitMedianaufteilungErsterZP	Linear	1,854	1	1,854	,286
Fehler(Faktor1)	Linear	162,146	25	6,486	

Tests der Innersubjektkontraste

Maß: MASS_1

Quelle	Faktor1	Sig.
Faktor1	Linear	,984
Faktor1 * STAITraitMedianaufteilungErsterZP	Linear	,598
Fehler(Faktor1)	Linear	

Tests der Zwischensubjekteffekte

Maß: MASS_1

Transformierte Variable: Mittel

Quelle	Typ III Quadratsumme	df	Mittel der Quadrate	F	Sig.
Konstanter Term	6078,291	1	6078,291	166,971	,000
STAITraitMedianaufteilung ErsterZP	6,513	1	6,513	,179	,676
Fehler	910,080	25	36,403		

Profilplots

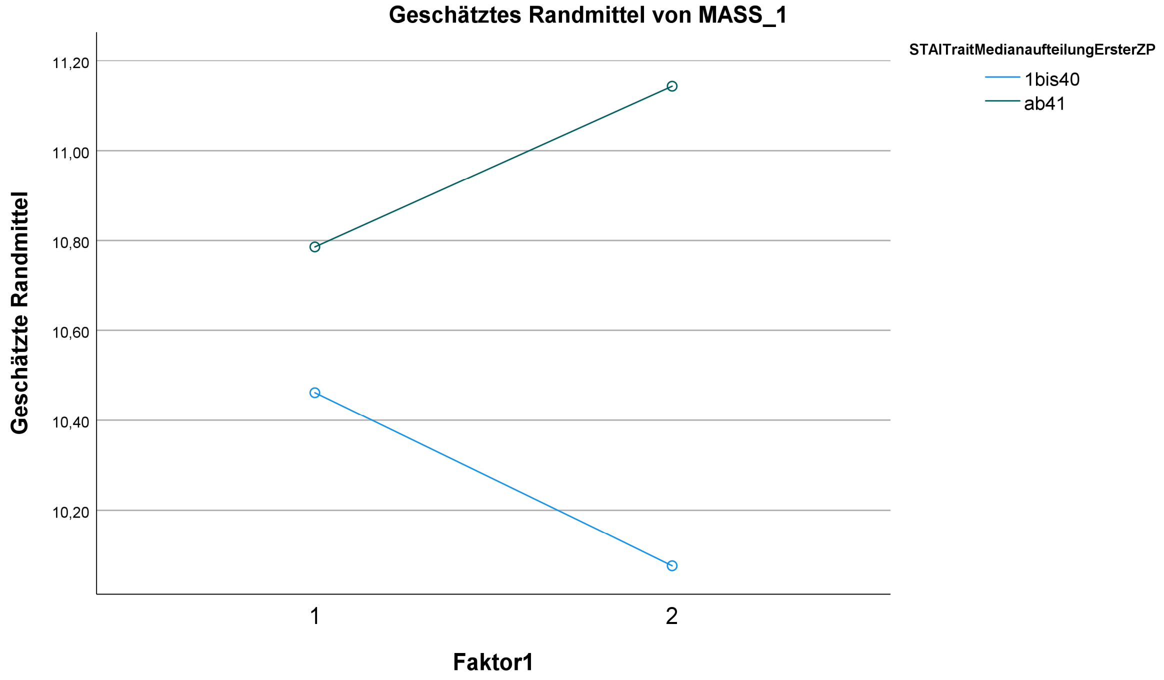


```
GLM PSSersterZP PSSzweiterZP BY STAITraitMedianaufteilungErsterZP
  /WSFACTOR=Faktor1 2 Polynomial
  /METHOD=SSTYPE(3)
  /PLOT=PROFILE(Faktor1*STAITraitMedianaufteilungErsterZP) TYPE=LINE ERRORBAR=NO MEA
EFERENCE=NO
    YAXIS=AUTO
  /CRITERIA=ALPHA(.05)
  /WSDESIGN=Faktor1
  /DESIGN=STAITraitMedianaufteilungErsterZP.
```

Allgemeines lineares Modell

Innersubjektfaktoren

Maß: MASS_1

Faktor1	Abhängige Variable
1	PSSersterZP
2	PSSzweiterZP

Zwischensubjektfaktoren

		Wertbeschriftung	N
STAITraitMedianaufteilung ErsterZP	1,00	1bis40	13
	2,00	ab41	14

Multivariate Tests[a]

Effekt		Wert	F	Hypothese df
Faktor1	Pillai-Spur	,151	4,464[b]	1,000
	Wilks-Lambda	,849	4,464[b]	1,000
	Hotelling-Spur	,179	4,464[b]	1,000
	Größte charakteristische Wurzel nach Roy	,179	4,464[b]	1,000
Faktor1 * STAITraitMedianaufteilung ErsterZP	Pillai-Spur	,002	,044[b]	1,000
	Wilks-Lambda	,998	,044[b]	1,000
	Hotelling-Spur	,002	,044[b]	1,000
	Größte charakteristische Wurzel nach Roy	,002	,044[b]	1,000

Multivariate Tests[a]

Effekt		Fehler df	Sig.
Faktor1	Pillai-Spur	25,000	,045
	Wilks-Lambda	25,000	,045
	Hotelling-Spur	25,000	,045
	Größte charakteristische Wurzel nach Roy	25,000	,045
Faktor1 * STAITraitMedianaufteilung ErsterZP	Pillai-Spur	25,000	,836
	Wilks-Lambda	25,000	,836
	Hotelling-Spur	25,000	,836
	Größte charakteristische Wurzel nach Roy	25,000	,836

a. Design: Konstanter Term + STAITraitMedianaufteilungErsterZP
Innersubjektdesign: Faktor1

b. Exakte Statistik

Mauchly-Test auf Sphärizität[a]

Maß: MASS_1

Innersubjekteffekt	Mauchly-W	Ungefähres Chi-Quadrat	df	Sig.	Epsilon[b] Greenhouse-Geisser
Faktor1	1,000	,000	0	.	1,000

Mauchly-Test auf Sphärizität[a]

Maß: MASS_1

Innersubjekteffekt	Epsilon[b] Huynh-Feldt (HF)	Untergrenze
Faktor1	1,000	1,000

Prüft die Nullhypothese, dass sich die Fehlerkovarianz-Matrix der orthonormalisierten transformierten abhängigen Variablen proportional zur Einheitsmatrix verhält.

a. Design: Konstanter Term + STAITraitMedianaufteilungErsterZP
Innersubjektdesign: Faktor1

b. Kann zum Korrigieren der Freiheitsgrade für die gemittelten Signifikanztests verwendet werden. In der Tabelle mit den Tests der Effekte innerhalb der Subjekte werden korrigierte Tests angezeigt.

Tests der Innersubjekteffekte

Maß: MASS_1

Quelle		Typ III Quadratsumme	df	Mittel der Quadrate
Faktor1	Sphärizität angenommen	19,077	1	19,077
	Greenhouse-Geisser	19,077	1,000	19,077
	Huynh-Feldt (HF)	19,077	1,000	19,077
	Untergrenze	19,077	1,000	19,077
Faktor1 * STAITraitMedianaufteilungErsterZP	Sphärizität angenommen	,188	1	,188
	Greenhouse-Geisser	,188	1,000	,188
	Huynh-Feldt (HF)	,188	1,000	,188
	Untergrenze	,188	1,000	,188
Fehler(Faktor1)	Sphärizität angenommen	106,849	25	4,274
	Greenhouse-Geisser	106,849	25,000	4,274
	Huynh-Feldt (HF)	106,849	25,000	4,274
	Untergrenze	106,849	25,000	4,274

Tests der Innersubjekteffekte

Maß: MASS_1

Quelle		F	Sig.
Faktor1	Sphärizität angenommen	4,464	,045
	Greenhouse-Geisser	4,464	,045
	Huynh-Feldt (HF)	4,464	,045
	Untergrenze	4,464	,045
Faktor1 * STAITraitMedianaufteilung ErsterZP	Sphärizität angenommen	,044	,836
	Greenhouse-Geisser	,044	,836
	Huynh-Feldt (HF)	,044	,836
	Untergrenze	,044	,836
Fehler(Faktor1)	Sphärizität angenommen		
	Greenhouse-Geisser		
	Huynh-Feldt (HF)		
	Untergrenze		

Tests der Innersubjektkontraste

Maß: MASS_1

Quelle	Faktor1	Typ III Quadratsumme	df	Mittel der Quadrate	F
Faktor1	Linear	19,077	1	19,077	4,464
Faktor1 * STAITraitMedianaufteilung ErsterZP	Linear	,188	1	,188	,044
Fehler(Faktor1)	Linear	106,849	25	4,274	

Tests der Innersubjektkontraste

Maß: MASS_1

Quelle	Faktor1	Sig.
Faktor1	Linear	,045
Faktor1 * STAITraitMedianaufteilung ErsterZP	Linear	,836
Fehler(Faktor1)	Linear	

Tests der Zwischensubjekteffekte

Maß: MASS_1

Transformierte Variable: Mittel

Quelle	Typ III Quadratsumme	df	Mittel der Quadrate	F	Sig.
Konstanter Term	21520,029	1	21520,029	523,060	,000
STAITraitMedianaufteilung ErsterZP	1156,252	1	1156,252	28,104	,000
Fehler	1028,563	25	41,143		

Profilplots

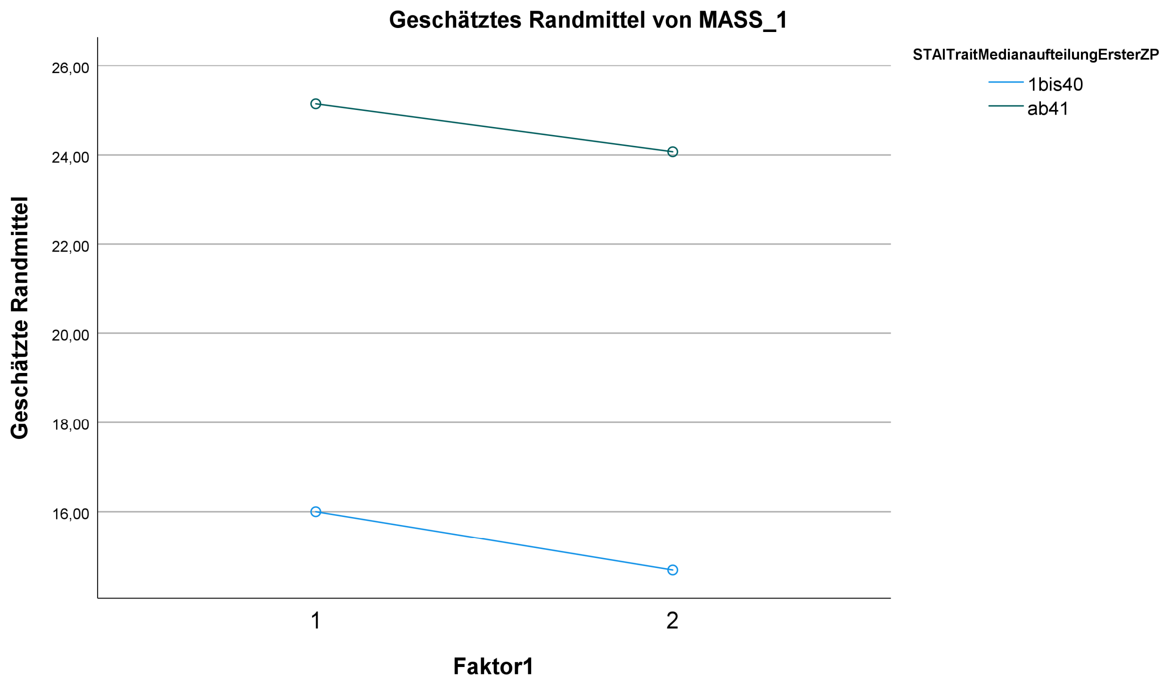


```
GLM GBBGesamtskalaBersterZP GBBGesamtskalaBzweiterZP BY STAITraitMedianaufteilungErs
rZP
  /WSFACTOR=Faktor1 2 Polynomial
  /METHOD=SSTYPE(3)
  /PLOT=PROFILE(Faktor1*STAITraitMedianaufteilungErsterZP) TYPE=LINE ERRORBAR=NO MEA
EFERENCE=NO
    YAXIS=AUTO
  /CRITERIA=ALPHA(.05)
  /WSDESIGN=Faktor1
  /DESIGN=STAITraitMedianaufteilungErsterZP.
```

Allgemeines lineares Modell

Innersubjektfaktoren

Maß: MASS_1

Faktor1	Abhängige Variable
1	GBBGesamtskalaBersterZP
2	GBBGesamtskalaBzweiterZP

Zwischensubjektfaktoren

		Wertbeschriftung	N
STAITraitMedianaufteilung ErsterZP	1,00	1bis40	12
	2,00	ab41	14

Multivariate Tests[a]

Effekt		Wert	F	Hypothese df
Faktor1	Pillai-Spur	,008	,206[b]	1,000
	Wilks-Lambda	,992	,206[b]	1,000
	Hotelling-Spur	,009	,206[b]	1,000
	Größte charakteristische Wurzel nach Roy	,009	,206[b]	1,000
Faktor1 * STAITraitMedianaufteilung ErsterZP	Pillai-Spur	,000	,000[b]	1,000
	Wilks-Lambda	1,000	,000[b]	1,000
	Hotelling-Spur	,000	,000[b]	1,000
	Größte charakteristische Wurzel nach Roy	,000	,000[b]	1,000

Multivariate Tests[a]

Effekt		Fehler df	Sig.
Faktor1	Pillai-Spur	24,000	,654
	Wilks-Lambda	24,000	,654
	Hotelling-Spur	24,000	,654
	Größte charakteristische Wurzel nach Roy	24,000	,654
Faktor1 * STAITraitMedianaufteilung ErsterZP	Pillai-Spur	24,000	,990
	Wilks-Lambda	24,000	,990
	Hotelling-Spur	24,000	,990
	Größte charakteristische Wurzel nach Roy	24,000	,990

a. Design: Konstanter Term + STAITraitMedianaufteilungErsterZP
Innersubjektdesign: Faktor1

b. Exakte Statistik

Mauchly-Test auf Sphärizität[a]

Maß: MASS_1

Innersubjekteffekt	Mauchly-W	Ungefähres Chi-Quadrat	df	Sig.	Epsilon[b] Greenhouse-Geisser
Faktor1	1,000	,000	0	.	1,000

Mauchly-Test auf Sphärizität[a]

Maß: MASS_1

Innersubjekteffekt	Epsilon[b] Huynh-Feldt (HF)	Untergrenze
Faktor1	1,000	1,000

Prüft die Nullhypothese, dass sich die Fehlerkovarianz-Matrix der orthonormalisierten transformierten abhängigen Variablen proportional zur Einheitsmatrix verhält.

a. Design: Konstanter Term + STAITraitMedianaufteilungErsterZP
Innersubjektdesign: Faktor1

b. Kann zum Korrigieren der Freiheitsgrade für die gemittelten Signifikanztests verwendet werden. In der Tabelle mit den Tests der Effekte innerhalb der Subjekte werden korrigierte Tests angezeigt.

Tests der Innersubjekteffekte

Maß: MASS_1

Quelle		Typ III Quadratsumme	df	Mittel der Quadrate
Faktor1	Sphärizität angenommen	16,011	1	16,011
	Greenhouse-Geisser	16,011	1,000	16,011
	Huynh-Feldt (HF)	16,011	1,000	16,011
	Untergrenze	16,011	1,000	16,011
Faktor1 * STAITraitMedianaufteilungErsterZP	Sphärizität angenommen	,011	1	,011
	Greenhouse-Geisser	,011	1,000	,011
	Huynh-Feldt (HF)	,011	1,000	,011
	Untergrenze	,011	1,000	,011
Fehler(Faktor1)	Sphärizität angenommen	1868,315	24	77,846
	Greenhouse-Geisser	1868,315	24,000	77,846
	Huynh-Feldt (HF)	1868,315	24,000	77,846
	Untergrenze	1868,315	24,000	77,846

Tests der Innersubjekteffekte

Maß: MASS_1

Quelle		F	Sig.
Faktor1	Sphärizität angenommen	,206	,654
	Greenhouse-Geisser	,206	,654
	Huynh-Feldt (HF)	,206	,654
	Untergrenze	,206	,654
Faktor1 * STAITraitMedianaufteilung ErsterZP	Sphärizität angenommen	,000	,990
	Greenhouse-Geisser	,000	,990
	Huynh-Feldt (HF)	,000	,990
	Untergrenze	,000	,990
Fehler(Faktor1)	Sphärizität angenommen		
	Greenhouse-Geisser		
	Huynh-Feldt (HF)		
	Untergrenze		

Tests der Innersubjektkontraste

Maß: MASS_1

Quelle	Faktor1	Typ III Quadratsumme	df	Mittel der Quadrate	F
Faktor1	Linear	16,011	1	16,011	,206
Faktor1 * STAITraitMedianaufteilung ErsterZP	Linear	,011	1	,011	,000
Fehler(Faktor1)	Linear	1868,315	24	77,846	

Tests der Innersubjektkontraste

Maß: MASS_1

Quelle	Faktor1	Sig.
Faktor1	Linear	,654
Faktor1 * STAITraitMedianaufteilung ErsterZP	Linear	,990
Fehler(Faktor1)	Linear	

Tests der Zwischensubjekteffekte

Maß: MASS_1

Transformierte Variable: Mittel

Quelle	Typ III Quadratsumme	df	Mittel der Quadrate	F	Sig.
Konstanter Term	28150,770	1	28150,770	143,316	,000
STAITraitMedianaufteilung ErsterZP	182,308	1	182,308	,928	,345
Fehler	4714,173	24	196,424		

Profilplots

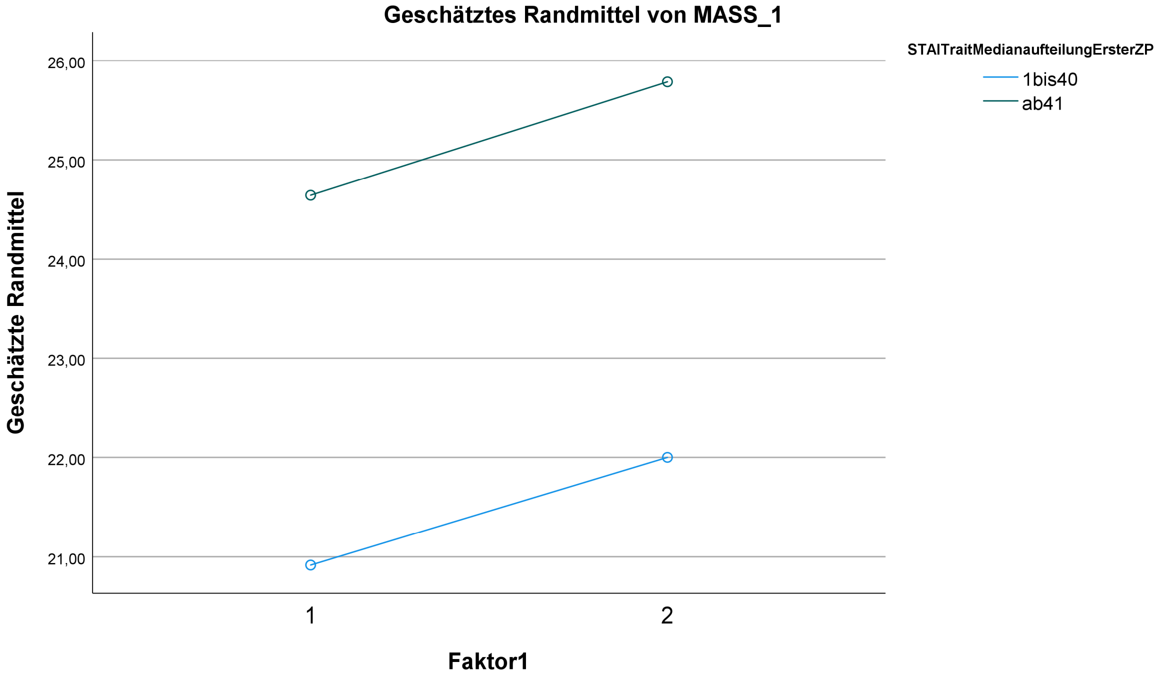


```
GLM TICSersterZPsscs TICSzweiterZPsscs BY STAITraitMedianaufteilungErsterZP
  /WSFACTOR=Faktor1 2 Polynomial
  /METHOD=SSTYPE(3)
  /PLOT=PROFILE(Faktor1*STAITraitMedianaufteilungErsterZP) TYPE=LINE ERRORBAR=NO MEA
EFERENCE=NO
    YAXIS=AUTO
  /CRITERIA=ALPHA(.05)
  /WSDESIGN=Faktor1
  /DESIGN=STAITraitMedianaufteilungErsterZP.
```

Allgemeines lineares Modell

Innersubjektfaktoren

Maß: MASS_1

Faktor1	Abhängige Variable
1	TICSersterZPsscs
2	TICSzweiterZPsscs

Zwischensubjektfaktoren

		Wertbeschriftung	N
STAITraitMedianaufteilung ErsterZP	1,00	1bis40	12
	2,00	ab41	14

Multivariate Tests[a]

Effekt		Wert	F	Hypothese df
Faktor1	Pillai-Spur	,051	1,278[b]	1,000
	Wilks-Lambda	,949	1,278[b]	1,000
	Hotelling-Spur	,053	1,278[b]	1,000
	Größte charakteristische Wurzel nach Roy	,053	1,278[b]	1,000
Faktor1 * STAITraitMedianaufteilung ErsterZP	Pillai-Spur	,240	7,575[b]	1,000
	Wilks-Lambda	,760	7,575[b]	1,000
	Hotelling-Spur	,316	7,575[b]	1,000
	Größte charakteristische Wurzel nach Roy	,316	7,575[b]	1,000

Multivariate Tests[a]

Effekt		Fehler df	Sig.
Faktor1	Pillai-Spur	24,000	,270
	Wilks-Lambda	24,000	,270
	Hotelling-Spur	24,000	,270
	Größte charakteristische Wurzel nach Roy	24,000	,270
Faktor1 * STAITraitMedianaufteilung ErsterZP	Pillai-Spur	24,000	,011
	Wilks-Lambda	24,000	,011
	Hotelling-Spur	24,000	,011
	Größte charakteristische Wurzel nach Roy	24,000	,011

a. Design: Konstanter Term + STAITraitMedianaufteilungErsterZP
Innersubjektdesign: Faktor1

b. Exakte Statistik

Mauchly-Test auf Sphärizität[a]

Maß: MASS_1

Innersubjekteffekt	Mauchly-W	Ungefähres Chi-Quadrat	df	Sig.	Epsilon[b] Greenhouse-Geisser
Faktor1	1,000	,000	0	.	1,000

Mauchly-Test auf Sphärizität[a]

Maß: MASS_1

Innersubjekteffekt	Epsilon[b]	
	Huynh-Feldt (HF)	Untergrenze
Faktor1	1,000	1,000

Prüft die Nullhypothese, dass sich die Fehlerkovarianz-Matrix der orthonormalisierten transformierten abhängigen Variablen proportional zur Einheitsmatrix verhält.

a. Design: Konstanter Term + STAITraitMedianaufteilungErsterZP
Innersubjektdesign: Faktor1

b. Kann zum Korrigieren der Freiheitsgrade für die gemittelten Signifikanztests verwendet werden. In der Tabelle mit den Tests der Effekte innerhalb der Subjekte werden korrigierte Tests angezeigt.

Tests der Innersubjekteffekte

Maß: MASS_1

Quelle		Typ III Quadratsumme	df	Mittel der Quadrate
Faktor1	Sphärizität angenommen	107,704	1	107,704
	Greenhouse-Geisser	107,704	1,000	107,704
	Huynh-Feldt (HF)	107,704	1,000	107,704
	Untergrenze	107,704	1,000	107,704
Faktor1 * STAITraitMedianaufteilungErsterZP	Sphärizität angenommen	638,627	1	638,627
	Greenhouse-Geisser	638,627	1,000	638,627
	Huynh-Feldt (HF)	638,627	1,000	638,627
	Untergrenze	638,627	1,000	638,627
Fehler(Faktor1)	Sphärizität angenommen	2023,315	24	84,305
	Greenhouse-Geisser	2023,315	24,000	84,305
	Huynh-Feldt (HF)	2023,315	24,000	84,305
	Untergrenze	2023,315	24,000	84,305

Tests der Innersubjekteffekte

Maß: MASS_1

Quelle		F	Sig.
Faktor1	Sphärizität angenommen	1,278	,270
	Greenhouse-Geisser	1,278	,270
	Huynh-Feldt (HF)	1,278	,270
	Untergrenze	1,278	,270
Faktor1 * STAITraitMedianaufteilung ErsterZP	Sphärizität angenommen	7,575	,011
	Greenhouse-Geisser	7,575	,011
	Huynh-Feldt (HF)	7,575	,011
	Untergrenze	7,575	,011
Fehler(Faktor1)	Sphärizität angenommen		
	Greenhouse-Geisser		
	Huynh-Feldt (HF)		
	Untergrenze		

Tests der Innersubjektkontraste

Maß: MASS_1

Quelle	Faktor1	Typ III Quadratsumme	df	Mittel der Quadrate	F
Faktor1	Linear	107,704	1	107,704	1,278
Faktor1 * STAITraitMedianaufteilung ErsterZP	Linear	638,627	1	638,627	7,575
Fehler(Faktor1)	Linear	2023,315	24	84,305	

Tests der Innersubjektkontraste

Maß: MASS_1

Quelle	Faktor1	Sig.
Faktor1	Linear	,270
Faktor1 * STAITraitMedianaufteilung ErsterZP	Linear	,011
Fehler(Faktor1)	Linear	

Tests der Zwischensubjekteffekte

Maß: MASS_1

Transformierte Variable: Mittel

Quelle	Typ III Quadratsumme	df	Mittel der Quadrate	F	Sig.
Konstanter Term	16544,011	1	16544,011	251,729	,000
STAITraitMedianaufteilung ErsterZP	72,165	1	72,165	1,098	,305
Fehler	1577,315	24	65,721		

Profilplots

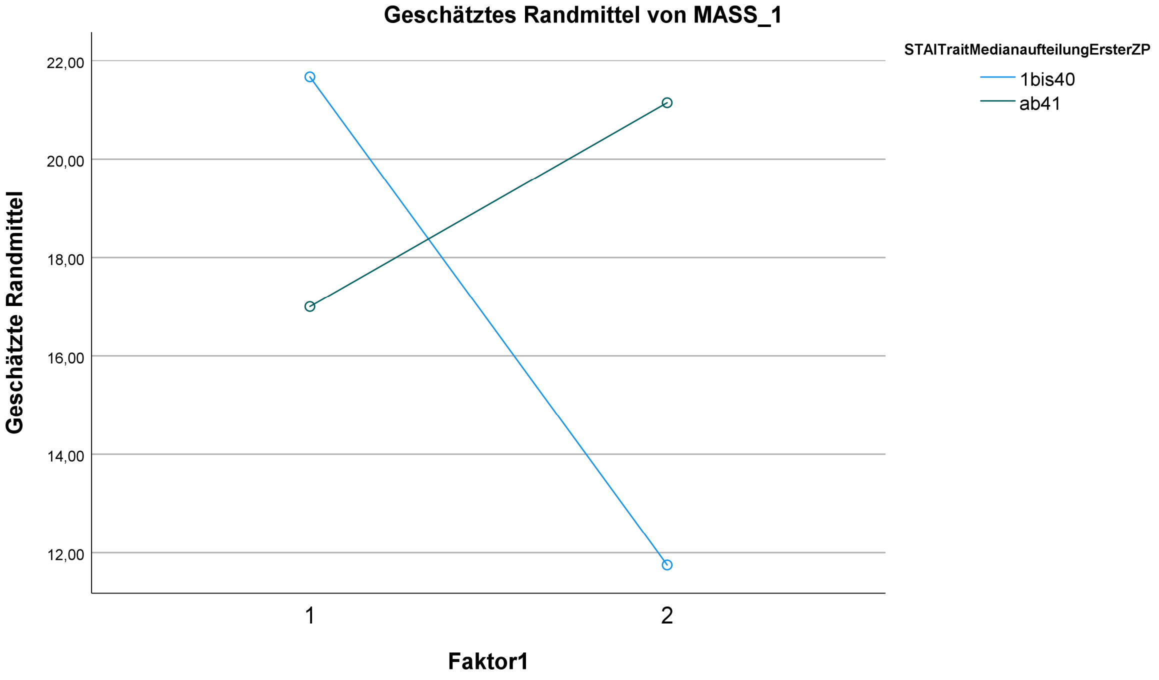


```
GLM MBIeeersterZP MBIeezweiterZP BY STAITraitMedianaufteilungErsterZP
  /WSFACTOR=Faktor1 2 Polynomial
  /METHOD=SSTYPE(3)
  /PLOT=PROFILE(Faktor1*STAITraitMedianaufteilungErsterZP) TYPE=LINE ERRORBAR=NO MEA
EFERENCE=NO
    YAXIS=AUTO
  /CRITERIA=ALPHA(.05)
  /WSDESIGN=Faktor1
  /DESIGN=STAITraitMedianaufteilungErsterZP.
```

Allgemeines lineares Modell

Innersubjektfaktoren

Maß: MASS_1

Faktor1	Abhängige Variable
1	MBleeersterZP
2	MBleezweiterZP

Zwischensubjektfaktoren

		Wertbeschriftung	N
STAITraitMedianaufteilung ErsterZP	1,00	1bis40	13
	2,00	ab41	12

Multivariate Tests[a]

Effekt		Wert	F	Hypothese df
Faktor1	Pillai-Spur	,012	,270[b]	1,000
	Wilks-Lambda	,988	,270[b]	1,000
	Hotelling-Spur	,012	,270[b]	1,000
	Größte charakteristische Wurzel nach Roy	,012	,270[b]	1,000
Faktor1 * STAITraitMedianaufteilung ErsterZP	Pillai-Spur	,037	,890[b]	1,000
	Wilks-Lambda	,963	,890[b]	1,000
	Hotelling-Spur	,039	,890[b]	1,000
	Größte charakteristische Wurzel nach Roy	,039	,890[b]	1,000

Multivariate Tests[a]

Effekt		Fehler df	Sig.
Faktor1	Pillai-Spur	23,000	,609
	Wilks-Lambda	23,000	,609
	Hotelling-Spur	23,000	,609
	Größte charakteristische Wurzel nach Roy	23,000	,609
Faktor1 * STAITraitMedianaufteilung ErsterZP	Pillai-Spur	23,000	,355
	Wilks-Lambda	23,000	,355
	Hotelling-Spur	23,000	,355
	Größte charakteristische Wurzel nach Roy	23,000	,355

a. Design: Konstanter Term + STAITraitMedianaufteilungErsterZP
Innersubjektdesign: Faktor1

b. Exakte Statistik

Mauchly-Test auf Sphärizität[a]

Maß: MASS_1

Innersubjekteffekt	Mauchly-W	Ungefähres Chi-Quadrat	df	Sig.	Epsilon[b]
					Greenhouse-Geisser
Faktor1	1,000	,000	0	.	1,000

Mauchly-Test auf Sphärizität[a]

Maß: MASS_1

Innersubjekteffekt	Epsilon[b]	
	Huynh-Feldt (HF)	Untergrenze
Faktor1	1,000	1,000

Prüft die Nullhypothese, dass sich die Fehlerkovarianz-Matrix der orthonormalisierten transformierten abhängigen Variablen proportional zur Einheitsmatrix verhält.

a. Design: Konstanter Term + STAITraitMedianaufteilungErsterZP
Innersubjektdesign: Faktor1

b. Kann zum Korrigieren der Freiheitsgrade für die gemittelten Signifikanztests verwendet werden. In der Tabelle mit den Tests der Effekte innerhalb der Subjekte werden korrigierte Tests angezeigt.

Tests der Innersubjekteffekte

Maß: MASS_1

Quelle		Typ III Quadratsumme	df	Mittel der Quadrate
Faktor1	Sphärizität angenommen	13,376	1	13,376
	Greenhouse-Geisser	13,376	1,000	13,376
	Huynh-Feldt (HF)	13,376	1,000	13,376
	Untergrenze	13,376	1,000	13,376
Faktor1 * STAITraitMedianaufteilungErsterZP	Sphärizität angenommen	44,176	1	44,176
	Greenhouse-Geisser	44,176	1,000	44,176
	Huynh-Feldt (HF)	44,176	1,000	44,176
	Untergrenze	44,176	1,000	44,176
Fehler(Faktor1)	Sphärizität angenommen	1141,304	23	49,622
	Greenhouse-Geisser	1141,304	23,000	49,622
	Huynh-Feldt (HF)	1141,304	23,000	49,622
	Untergrenze	1141,304	23,000	49,622

Tests der Innersubjekteffekte

Maß: MASS_1

Quelle		F	Sig.
Faktor1	Sphärizität angenommen	,270	,609
	Greenhouse-Geisser	,270	,609
	Huynh-Feldt (HF)	,270	,609
	Untergrenze	,270	,609
Faktor1 * STAITraitMedianaufteilung ErsterZP	Sphärizität angenommen	,890	,355
	Greenhouse-Geisser	,890	,355
	Huynh-Feldt (HF)	,890	,355
	Untergrenze	,890	,355
Fehler(Faktor1)	Sphärizität angenommen		
	Greenhouse-Geisser		
	Huynh-Feldt (HF)		
	Untergrenze		

Tests der Innersubjektkontraste

Maß: MASS_1

Quelle	Faktor1	Typ III Quadratsumme	df	Mittel der Quadrate	F
Faktor1	Linear	13,376	1	13,376	,270
Faktor1 * STAITraitMedianaufteilung ErsterZP	Linear	44,176	1	44,176	,890
Fehler(Faktor1)	Linear	1141,304	23	49,622	

Tests der Innersubjektkontraste

Maß: MASS_1

Quelle	Faktor1	Sig.
Faktor1	Linear	,609
Faktor1 * STAITraitMedianaufteilung ErsterZP	Linear	,355
Fehler(Faktor1)	Linear	

Tests der Zwischensubjekteffekte

Maß: MASS_1

Transformierte Variable: Mittel

Quelle	Typ III Quadratsumme	df	Mittel der Quadrate	F	Sig.
Konstanter Term	18686,837	1	18686,837	84,608	,000
STAITraitMedianaufteilung ErsterZP	1099,877	1	1099,877	4,980	,036
Fehler	5079,843	23	220,863		

Profilplots

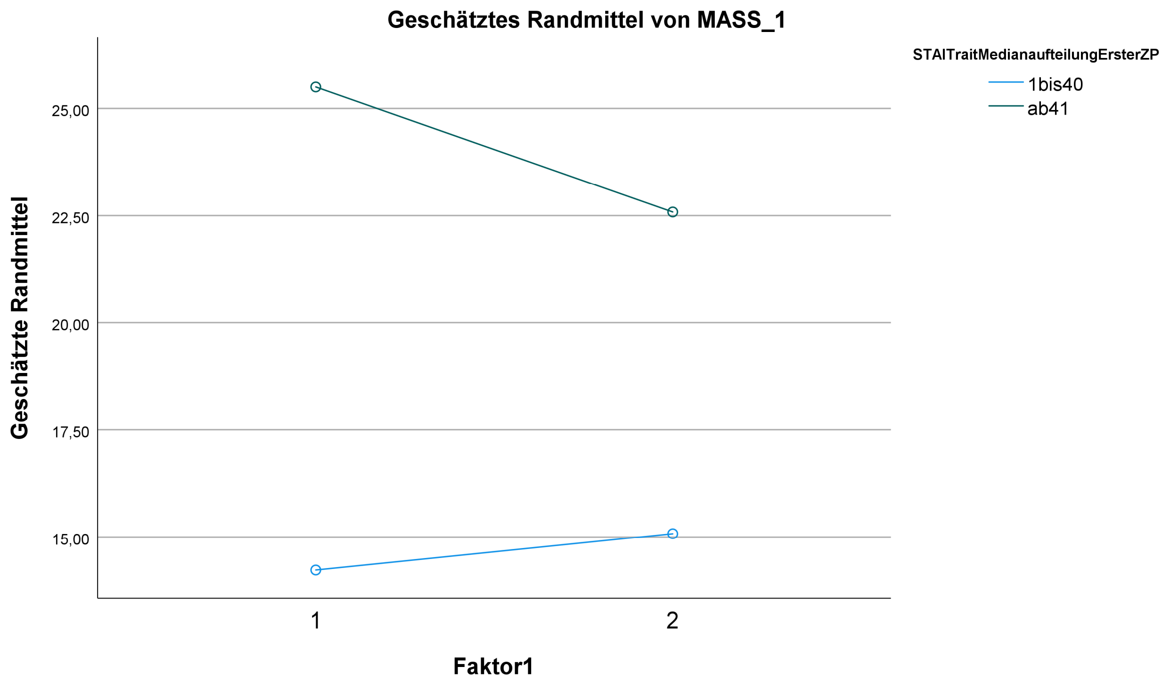


```
GLM MBIdersterZP MBIdzweiterZP BY STAITraitMedianaufteilungErsterZP
  /WSFACTOR=Faktor1 2 Polynomial
  /METHOD=SSTYPE(3)
  /PLOT=PROFILE(Faktor1*STAITraitMedianaufteilungErsterZP) TYPE=LINE ERRORBAR=NO MEA
EFERENCE=NO
    YAXIS=AUTO
  /CRITERIA=ALPHA(.05)
  /WSDESIGN=Faktor1
  /DESIGN=STAITraitMedianaufteilungErsterZP.
```

Allgemeines lineares Modell

Innersubjektfaktoren

Maß: MASS_1

Faktor1	Abhängige Variable
1	MBIdersterZP
2	MBIdzweiterZP

Zwischensubjektfaktoren

		Wertbeschriftung	N
STAITraitMedianaufteilung ErsterZP	1,00	1bis40	13
	2,00	ab41	12

Multivariate Tests[a]

Effekt		Wert	F	Hypothese df
Faktor1	Pillai-Spur	,020	,463[b]	1,000
	Wilks-Lambda	,980	,463[b]	1,000
	Hotelling-Spur	,020	,463[b]	1,000
	Größte charakteristische Wurzel nach Roy	,020	,463[b]	1,000
Faktor1 * STAITraitMedianaufteilung ErsterZP	Pillai-Spur	,053	1,287[b]	1,000
	Wilks-Lambda	,947	1,287[b]	1,000
	Hotelling-Spur	,056	1,287[b]	1,000
	Größte charakteristische Wurzel nach Roy	,056	1,287[b]	1,000

Multivariate Tests[a]

Effekt		Fehler df	Sig.
Faktor1	Pillai-Spur	23,000	,503
	Wilks-Lambda	23,000	,503
	Hotelling-Spur	23,000	,503
	Größte charakteristische Wurzel nach Roy	23,000	,503
Faktor1 * STAITraitMedianaufteilung ErsterZP	Pillai-Spur	23,000	,268
	Wilks-Lambda	23,000	,268
	Hotelling-Spur	23,000	,268
	Größte charakteristische Wurzel nach Roy	23,000	,268

a. Design: Konstanter Term + STAITraitMedianaufteilungErsterZP
Innersubjektdesign: Faktor1

b. Exakte Statistik

Mauchly-Test auf Sphärizität[a]

Maß: MASS_1

Innersubjekteffekt	Mauchly-W	Ungefähres Chi-Quadrat	df	Sig.	Epsilon[b] Greenhouse-Geisser
Faktor1	1,000	,000	0	.	1,000

Mauchly-Test auf Sphärizität[a]

Maß: MASS_1

Innersubjekteffekt	Epsilon[b]	
	Huynh-Feldt (HF)	Untergrenze
Faktor1	1,000	1,000

Prüft die Nullhypothese, dass sich die Fehlerkovarianz-Matrix der orthonormalisierten transformierten abhängigen Variablen proportional zur Einheitsmatrix verhält.

a. Design: Konstanter Term + STAITraitMedianaufteilungErsterZP
Innersubjektdesign: Faktor1

b. Kann zum Korrigieren der Freiheitsgrade für die gemittelten Signifikanztests verwendet werden. In der Tabelle mit den Tests der Effekte innerhalb der Subjekte werden korrigierte Tests angezeigt.

Tests der Innersubjekteffekte

Maß: MASS_1

Quelle		Typ III Quadratsumme	df	Mittel der Quadrate
Faktor1	Sphärizität angenommen	1,755	1	1,755
	Greenhouse-Geisser	1,755	1,000	1,755
	Huynh-Feldt (HF)	1,755	1,000	1,755
	Untergrenze	1,755	1,000	1,755
Faktor1 * STAITraitMedianaufteilungErsterZP	Sphärizität angenommen	4,875	1	4,875
	Greenhouse-Geisser	4,875	1,000	4,875
	Huynh-Feldt (HF)	4,875	1,000	4,875
	Untergrenze	4,875	1,000	4,875
Fehler(Faktor1)	Sphärizität angenommen	87,125	23	3,788
	Greenhouse-Geisser	87,125	23,000	3,788
	Huynh-Feldt (HF)	87,125	23,000	3,788
	Untergrenze	87,125	23,000	3,788

Tests der Innersubjekteffekte

Maß: MASS_1

Quelle		F	Sig.
Faktor1	Sphärizität angenommen	,463	,503
	Greenhouse-Geisser	,463	,503
	Huynh-Feldt (HF)	,463	,503
	Untergrenze	,463	,503
Faktor1 * STAITraitMedianaufteilung ErsterZP	Sphärizität angenommen	1,287	,268
	Greenhouse-Geisser	1,287	,268
	Huynh-Feldt (HF)	1,287	,268
	Untergrenze	1,287	,268
Fehler(Faktor1)	Sphärizität angenommen		
	Greenhouse-Geisser		
	Huynh-Feldt (HF)		
	Untergrenze		

Tests der Innersubjektkontraste

Maß: MASS_1

Quelle	Faktor1	Typ III Quadratsumme	df	Mittel der Quadrate	F
Faktor1	Linear	1,755	1	1,755	,463
Faktor1 * STAITraitMedianaufteilung ErsterZP	Linear	4,875	1	4,875	1,287
Fehler(Faktor1)	Linear	87,125	23	3,788	

Tests der Innersubjektkontraste

Maß: MASS_1

Quelle	Faktor1	Sig.
Faktor1	Linear	,503
Faktor1 * STAITraitMedianaufteilung ErsterZP	Linear	,268
Fehler(Faktor1)	Linear	

Tests der Zwischensubjekteffekte

Maß: MASS_1

Transformierte Variable: Mittel

Quelle	Typ III Quadratsumme	df	Mittel der Quadrate	F	Sig.
Konstanter Term	613,201	1	613,201	34,292	,000
STAITraitMedianaufteilung ErsterZP	131,041	1	131,041	7,328	,013
Fehler	411,279	23	17,882		

Profilplots

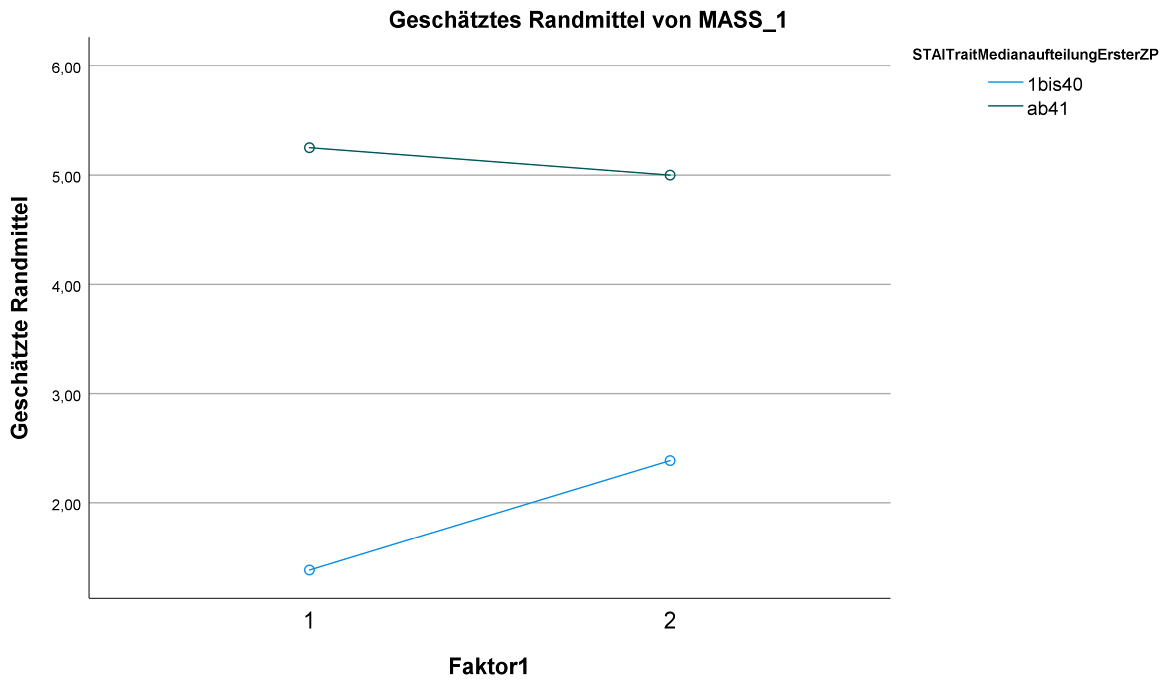


```
GLM MBIpaersterZP MBIpazweiterZP BY STAITraitMedianaufteilungErsterZP
  /WSFACTOR=Faktor1 2 Polynomial
  /METHOD=SSTYPE(3)
  /PLOT=PROFILE(Faktor1*STAITraitMedianaufteilungErsterZP) TYPE=LINE ERRORBAR=NO MEA
EFERENCE=NO
    YAXIS=AUTO
  /CRITERIA=ALPHA(.05)
  /WSDESIGN=Faktor1
  /DESIGN=STAITraitMedianaufteilungErsterZP.
```

Allgemeines lineares Modell

Innersubjektfaktoren

Maß: MASS_1

Faktor1	Abhängige Variable
1	MBIpaersterZP
2	MBIpazweiterZP

Zwischensubjektfaktoren

		Wertbeschriftung	N
STAITraitMedianaufteilung ErsterZP	1,00	1bis40	13
	2,00	ab41	12

Multivariate Tests[a]

Effekt		Wert	F	Hypothese df
Faktor1	Pillai-Spur	,000	,001[b]	1,000
	Wilks-Lambda	1,000	,001[b]	1,000
	Hotelling-Spur	,000	,001[b]	1,000
	Größte charakteristische Wurzel nach Roy	,000	,001[b]	1,000
Faktor1 * STAITraitMedianaufteilung ErsterZP	Pillai-Spur	,067	1,656[b]	1,000
	Wilks-Lambda	,933	1,656[b]	1,000
	Hotelling-Spur	,072	1,656[b]	1,000
	Größte charakteristische Wurzel nach Roy	,072	1,656[b]	1,000

Multivariate Tests[a]

Effekt		Fehler df	Sig.
Faktor1	Pillai-Spur	23,000	,979
	Wilks-Lambda	23,000	,979
	Hotelling-Spur	23,000	,979
	Größte charakteristische Wurzel nach Roy	23,000	,979
Faktor1 * STAITraitMedianaufteilung ErsterZP	Pillai-Spur	23,000	,211
	Wilks-Lambda	23,000	,211
	Hotelling-Spur	23,000	,211
	Größte charakteristische Wurzel nach Roy	23,000	,211

a. Design: Konstanter Term + STAITraitMedianaufteilungErsterZP
Innersubjektdesign: Faktor1

b. Exakte Statistik

Mauchly-Test auf Sphärizität[a]

Maß: MASS_1

Innersubjekteffekt	Mauchly-W	Ungefähres Chi-Quadrat	df	Sig.	Epsilon[b] Greenhouse-Geisser
Faktor1	1,000	,000	0	.	1,000

Mauchly-Test auf Sphärizität[a]

Maß: MASS_1

Innersubjekteffekt	Epsilon[b] Huynh-Feldt (HF)	Untergrenze
Faktor1	1,000	1,000

Prüft die Nullhypothese, dass sich die Fehlerkovarianz-Matrix der orthonormalisierten transformierten abhängigen Variablen proportional zur Einheitsmatrix verhält.

a. Design: Konstanter Term + STAITraitMedianaufteilungErsterZP
Innersubjektdesign: Faktor1

b. Kann zum Korrigieren der Freiheitsgrade für die gemittelten Signifikanztests verwendet werden. In der Tabelle mit den Tests der Effekte innerhalb der Subjekte werden korrigierte Tests angezeigt.

Tests der Innersubjekteffekte

Maß: MASS_1

Quelle		Typ III Quadratsumme	df	Mittel der Quadrate
Faktor1	Sphärizität angenommen	,022	1	,022
	Greenhouse-Geisser	,022	1,000	,022
	Huynh-Feldt (HF)	,022	1,000	,022
	Untergrenze	,022	1,000	,022
Faktor1 * STAITraitMedianaufteilung ErsterZP	Sphärizität angenommen	52,022	1	52,022
	Greenhouse-Geisser	52,022	1,000	52,022
	Huynh-Feldt (HF)	52,022	1,000	52,022
	Untergrenze	52,022	1,000	52,022
Fehler(Faktor1)	Sphärizität angenommen	722,458	23	31,411
	Greenhouse-Geisser	722,458	23,000	31,411
	Huynh-Feldt (HF)	722,458	23,000	31,411
	Untergrenze	722,458	23,000	31,411

Tests der Innersubjekteffekte

Maß: MASS_1

Quelle		F	Sig.
Faktor1	Sphärizität angenommen	,001	,979
	Greenhouse-Geisser	,001	,979
	Huynh-Feldt (HF)	,001	,979
	Untergrenze	,001	,979
Faktor1 * STAITraitMedianaufteilung ErsterZP	Sphärizität angenommen	1,656	,211
	Greenhouse-Geisser	1,656	,211
	Huynh-Feldt (HF)	1,656	,211
	Untergrenze	1,656	,211
Fehler(Faktor1)	Sphärizität angenommen		
	Greenhouse-Geisser		
	Huynh-Feldt (HF)		
	Untergrenze		

Tests der Innersubjektkontraste

Maß: MASS_1

Quelle	Faktor1	Typ III Quadratsumme	df	Mittel der Quadrate	F
Faktor1	Linear	,022	1	,022	,001
Faktor1 * STAITraitMedianaufteilung ErsterZP	Linear	52,022	1	52,022	1,656
Fehler(Faktor1)	Linear	722,458	23	31,411	

Tests der Innersubjektkontraste

Maß: MASS_1

Quelle	Faktor1	Sig.
Faktor1	Linear	,979
Faktor1 * STAITraitMedianaufteilung ErsterZP	Linear	,211
Fehler(Faktor1)	Linear	

Tests der Zwischensubjekteffekte

Maß: MASS_1

Transformierte Variable: Mittel

Quelle	Typ III Quadratsumme	df	Mittel der Quadrate	F	Sig.
Konstanter Term	67767,446	1	67767,446	488,594	,000
STAITraitMedianaufteilung ErsterZP	284,246	1	284,246	2,049	,166
Fehler	3190,074	23	138,699		

Profilplots

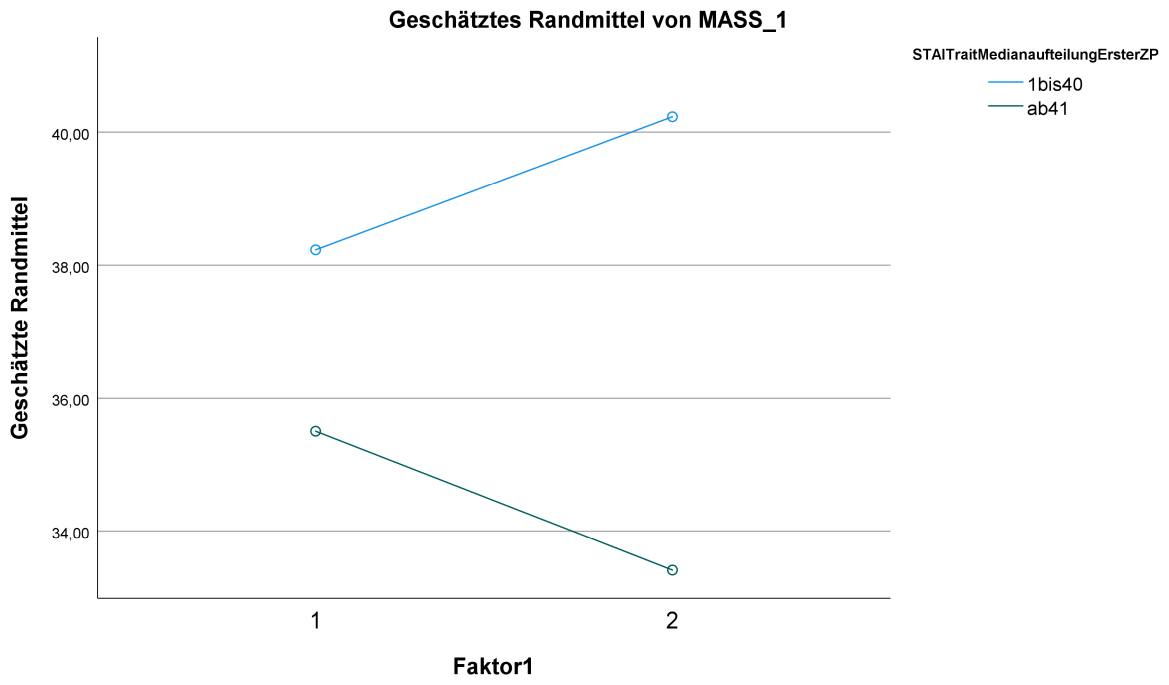


```
GLM WIersterZP WIzweiterZP BY STAITraitMedianaufteilungErsterZP
  /WSFACTOR=Faktor1 2 Polynomial
  /METHOD=SSTYPE(3)
  /PLOT=PROFILE(Faktor1*STAITraitMedianaufteilungErsterZP) TYPE=LINE ERRORBAR=NO MEA
EFERENCE=NO
    YAXIS=AUTO
  /CRITERIA=ALPHA(.05)
  /WSDESIGN=Faktor1
  /DESIGN=STAITraitMedianaufteilungErsterZP.
```

Allgemeines lineares Modell

Innersubjektfaktoren

Maß: MASS_1

Faktor1	Abhängige Variable
1	WIersterZP
2	WIzweiterZP

Zwischensubjektfaktoren

		Wertbeschriftung	N
STAITraitMedianaufteilung ErsterZP	1,00	1bis40	13
	2,00	ab41	14

Multivariate Tests[a]

Effekt		Wert	F	Hypothese df
Faktor1	Pillai-Spur	,199	6,208[b]	1,000
	Wilks-Lambda	,801	6,208[b]	1,000
	Hotelling-Spur	,248	6,208[b]	1,000
	Größte charakteristische Wurzel nach Roy	,248	6,208[b]	1,000
Faktor1 * STAITraitMedianaufteilung ErsterZP	Pillai-Spur	,016	,410[b]	1,000
	Wilks-Lambda	,984	,410[b]	1,000
	Hotelling-Spur	,016	,410[b]	1,000
	Größte charakteristische Wurzel nach Roy	,016	,410[b]	1,000

Multivariate Tests[a]

Effekt		Fehler df	Sig.
Faktor1	Pillai-Spur	25,000	,020
	Wilks-Lambda	25,000	,020
	Hotelling-Spur	25,000	,020
	Größte charakteristische Wurzel nach Roy	25,000	,020
Faktor1 * STAITraitMedianaufteilung ErsterZP	Pillai-Spur	25,000	,528
	Wilks-Lambda	25,000	,528
	Hotelling-Spur	25,000	,528
	Größte charakteristische Wurzel nach Roy	25,000	,528

a. Design: Konstanter Term + STAITraitMedianaufteilungErsterZP
Innersubjektdesign: Faktor1

b. Exakte Statistik

Mauchly-Test auf Sphärizität[a]

Maß: MASS_1

Innersubjekteffekt	Mauchly-W	Ungefähres Chi-Quadrat	df	Sig.	Epsilon[b] Greenhouse-Geisser
Faktor1	1,000	,000	0	.	1,000

Mauchly-Test auf Sphärizität[a]

Maß: MASS_1

Innersubjekteffekt	Epsilon[b] Huynh-Feldt (HF)	Untergrenze
Faktor1	1,000	1,000

Prüft die Nullhypothese, dass sich die Fehlerkovarianz-Matrix der orthonormalisierten transformierten abhängigen Variablen proportional zur Einheitsmatrix verhält.

a. Design: Konstanter Term + STAITraitMedianaufteilungErsterZP
Innersubjektdesign: Faktor1

b. Kann zum Korrigieren der Freiheitsgrade für die gemittelten Signifikanztests verwendet werden. In der Tabelle mit den Tests der Effekte innerhalb der Subjekte werden korrigierte Tests angezeigt.

Tests der Innersubjekteffekte

Maß: MASS_1

Quelle		Typ III Quadratsumme	df	Mittel der Quadrate
Faktor1	Sphärizität angenommen	6,108	1	6,108
	Greenhouse-Geisser	6,108	1,000	6,108
	Huynh-Feldt (HF)	6,108	1,000	6,108
	Untergrenze	6,108	1,000	6,108
Faktor1 * STAITraitMedianaufteilungErsterZP	Sphärizität angenommen	,404	1	,404
	Greenhouse-Geisser	,404	1,000	,404
	Huynh-Feldt (HF)	,404	1,000	,404
	Untergrenze	,404	1,000	,404
Fehler(Faktor1)	Sphärizität angenommen	24,596	25	,984
	Greenhouse-Geisser	24,596	25,000	,984
	Huynh-Feldt (HF)	24,596	25,000	,984
	Untergrenze	24,596	25,000	,984

Tests der Innersubjekteffekte

Maß: MASS_1

Quelle		F	Sig.
Faktor1	Sphärizität angenommen	6,208	,020
	Greenhouse-Geisser	6,208	,020
	Huynh-Feldt (HF)	6,208	,020
	Untergrenze	6,208	,020
Faktor1 * STAITraitMedianaufteilung ErsterZP	Sphärizität angenommen	,410	,528
	Greenhouse-Geisser	,410	,528
	Huynh-Feldt (HF)	,410	,528
	Untergrenze	,410	,528
Fehler(Faktor1)	Sphärizität angenommen		
	Greenhouse-Geisser		
	Huynh-Feldt (HF)		
	Untergrenze		

Tests der Innersubjektkontraste

Maß: MASS_1

Quelle	Faktor1	Typ III Quadratsumme	df	Mittel der Quadrate	F
Faktor1	Linear	6,108	1	6,108	6,208
Faktor1 * STAITraitMedianaufteilung ErsterZP	Linear	,404	1	,404	,410
Fehler(Faktor1)	Linear	24,596	25	,984	

Tests der Innersubjektkontraste

Maß: MASS_1

Quelle	Faktor1	Sig.
Faktor1	Linear	,020
Faktor1 * STAITraitMedianaufteilung ErsterZP	Linear	,528
Fehler(Faktor1)	Linear	

Tests der Zwischensubjekteffekte

Maß: MASS_1

Transformierte Variable: Mittel

Quelle	Typ III Quadratsumme	df	Mittel der Quadrate	F	Sig.
Konstanter Term	599,342	1	599,342	56,973	,000
STAITraitMedianaufteilung ErsterZP	52,601	1	52,601	5,000	,035
Fehler	262,992	25	10,520		

Profilplots

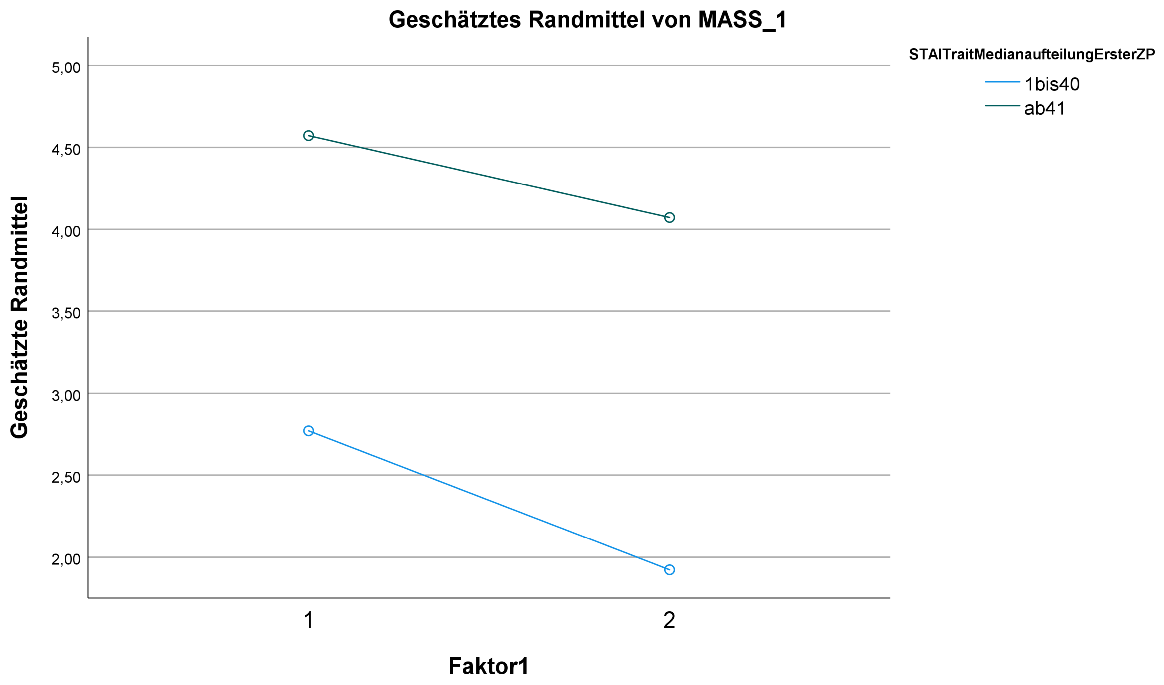


```
GLM BVIersterZP BVIzweiterZP BY STAITraitMedianaufteilungErsterZP
  /WSFACTOR=Faktor1 2 Polynomial
  /METHOD=SSTYPE(3)
  /PLOT=PROFILE(Faktor1*STAITraitMedianaufteilungErsterZP) TYPE=LINE ERRORBAR=NO MEA
EFERENCE=NO
    YAXIS=AUTO
  /CRITERIA=ALPHA(.05)
  /WSDESIGN=Faktor1
  /DESIGN=STAITraitMedianaufteilungErsterZP.
```

Allgemeines lineares Modell

Innersubjektfaktoren

Maß: MASS_1

Faktor1	Abhängige Variable
1	BVIersterZP
2	BVIzweiterZP

Zwischensubjektfaktoren

		Wertbeschriftung	N
STAITraitMedianaufteilung ErsterZP	1,00	1bis40	13
	2,00	ab41	14

Multivariate Tests[a]

Effekt		Wert	F	Hypothese df
Faktor1	Pillai-Spur	,104	2,895[b]	1,000
	Wilks-Lambda	,896	2,895[b]	1,000
	Hotelling-Spur	,116	2,895[b]	1,000
	Größte charakteristische Wurzel nach Roy	,116	2,895[b]	1,000
Faktor1 * STAITraitMedianaufteilung ErsterZP	Pillai-Spur	,001	,030[b]	1,000
	Wilks-Lambda	,999	,030[b]	1,000
	Hotelling-Spur	,001	,030[b]	1,000
	Größte charakteristische Wurzel nach Roy	,001	,030[b]	1,000

Multivariate Tests[a]

Effekt		Fehler df	Sig.
Faktor1	Pillai-Spur	25,000	,101
	Wilks-Lambda	25,000	,101
	Hotelling-Spur	25,000	,101
	Größte charakteristische Wurzel nach Roy	25,000	,101
Faktor1 * STAITraitMedianaufteilung ErsterZP	Pillai-Spur	25,000	,863
	Wilks-Lambda	25,000	,863
	Hotelling-Spur	25,000	,863
	Größte charakteristische Wurzel nach Roy	25,000	,863

a. Design: Konstanter Term + STAITraitMedianaufteilungErsterZP
Innersubjektdesign: Faktor1

b. Exakte Statistik

Mauchly-Test auf Sphärizität[a]

Maß: MASS_1

Innersubjekteffekt	Mauchly-W	Ungefähres Chi-Quadrat	df	Sig.	Epsilon[b] Greenhouse-Geisser
Faktor1	1,000	,000	0	.	1,000

Mauchly-Test auf Sphärizität[a]

Maß: MASS_1

Innersubjekteffekt	Epsilon[b] Huynh-Feldt (HF)	Untergrenze
Faktor1	1,000	1,000

Prüft die Nullhypothese, dass sich die Fehlerkovarianz-Matrix der orthonormalisierten transformierten abhängigen Variablen proportional zur Einheitsmatrix verhält.

a. Design: Konstanter Term + STAITraitMedianaufteilungErsterZP
Innersubjektdesign: Faktor1

b. Kann zum Korrigieren der Freiheitsgrade für die gemittelten Signifikanztests verwendet werden. In der Tabelle mit den Tests der Effekte innerhalb der Subjekte werden korrigierte Tests angezeigt.

Tests der Innersubjekteffekte

Maß: MASS_1

Quelle		Typ III Quadratsumme	df	Mittel der Quadrate
Faktor1	Sphärizität angenommen	31,661	1	31,661
	Greenhouse-Geisser	31,661	1,000	31,661
	Huynh-Feldt (HF)	31,661	1,000	31,661
	Untergrenze	31,661	1,000	31,661
Faktor1 * STAITraitMedianaufteilung ErsterZP	Sphärizität angenommen	,331	1	,331
	Greenhouse-Geisser	,331	1,000	,331
	Huynh-Feldt (HF)	,331	1,000	,331
	Untergrenze	,331	1,000	,331
Fehler(Faktor1)	Sphärizität angenommen	273,387	25	10,935
	Greenhouse-Geisser	273,387	25,000	10,935
	Huynh-Feldt (HF)	273,387	25,000	10,935
	Untergrenze	273,387	25,000	10,935

Tests der Innersubjekteffekte

Maß: MASS_1

Quelle		F	Sig.
Faktor1	Sphärizität angenommen	2,895	,101
	Greenhouse-Geisser	2,895	,101
	Huynh-Feldt (HF)	2,895	,101
	Untergrenze	2,895	,101
Faktor1 * STAITraitMedianaufteilung ErsterZP	Sphärizität angenommen	,030	,863
	Greenhouse-Geisser	,030	,863
	Huynh-Feldt (HF)	,030	,863
	Untergrenze	,030	,863
Fehler(Faktor1)	Sphärizität angenommen		
	Greenhouse-Geisser		
	Huynh-Feldt (HF)		
	Untergrenze		

Tests der Innersubjektkontraste

Maß: MASS_1

Quelle	Faktor1	Typ III Quadratsumme	df	Mittel der Quadrate	F
Faktor1	Linear	31,661	1	31,661	2,895
Faktor1 * STAITraitMedianaufteilung ErsterZP	Linear	,331	1	,331	,030
Fehler(Faktor1)	Linear	273,387	25	10,935	

Tests der Innersubjektkontraste

Maß: MASS_1

Quelle	Faktor1	Sig.
Faktor1	Linear	,101
Faktor1 * STAITraitMedianaufteilung ErsterZP	Linear	,863
Fehler(Faktor1)	Linear	

Tests der Zwischensubjekteffekte

Maß: MASS_1

Transformierte Variable: Mittel

Quelle	Typ III Quadratsumme	df	Mittel der Quadrate	F	Sig.
Konstanter Term	103250,003	1	103250,003	1183,797	,000
STAITraitMedianaufteilung ErsterZP	1730,334	1	1730,334	19,839	,000
Fehler	2180,485	25	87,219		

Profilplots

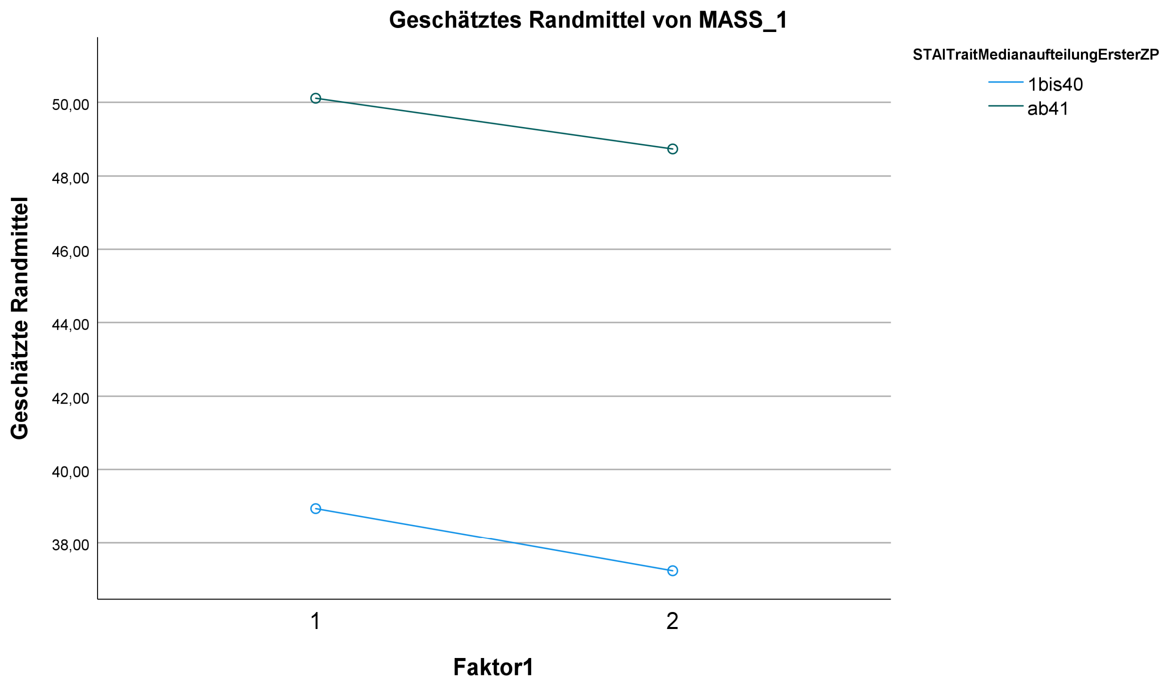


```
GET
  FILE='C:\Users\baranyia\AppData\Local\Microsoft\Windows\INetCache\Content.Outlook\
PFBIOG\Dateneingabe DA aktuell_ (002).sav'.
DATASET NAME DataSet1 WINDOW=FRONT.
DESCRIPTIVES VARIABLES=STAITraitersterZP
  /STATISTICS=MEAN STDDEV MIN MAX.
```

Deskriptive Statistik

```
[DataSet1] C:\Users\baranyia\AppData\Local\Microsoft\Windows\INetCache\Content.Outlo
\WFPFBIOG\Dateneingabe DA aktuell_ (002).sav
```

Deskriptive Statistiken

	N	Minimum	Maximum	Mittelwert	Std.-Abweichung
STAITraitersterZP	44	23,00	61,00	41,7727	10,41882
Gültige Werte (listenweise)	44				

```
FREQUENCIES VARIABLES=STAITraitersterZP
  /STATISTICS=MEDIAN
  /ORDER=ANALYSIS.
```

Häufigkeiten

Statistiken

STAITraitersterZP

N	Gültig	44
	Fehlend	36
Median		40,0000

STAITraitersterZP

		Häufigkeit	Prozent	Gültige Prozente	Kumulierte Prozente
Gültig	23,00	1	1,3	2,3	2,3
	25,00	1	1,3	2,3	4,5
	28,00	1	1,3	2,3	6,8
	29,00	2	2,5	4,5	11,4
	30,00	3	3,8	6,8	18,2
	31,00	2	2,5	4,5	22,7
	33,00	2	2,5	4,5	27,3
	34,00	1	1,3	2,3	29,5
	35,00	1	1,3	2,3	31,8
	38,00	4	5,0	9,1	40,9
	39,00	2	2,5	4,5	45,5
	40,00	3	3,8	6,8	52,3
	42,00	1	1,3	2,3	54,5
	43,00	1	1,3	2,3	56,8
	44,00	1	1,3	2,3	59,1
	45,00	3	3,8	6,8	65,9
	46,00	2	2,5	4,5	70,5
	47,00	1	1,3	2,3	72,7
	48,00	2	2,5	4,5	77,3
	50,00	1	1,3	2,3	79,5
	55,00	3	3,8	6,8	86,4

STAITraitersterZP

		Häufigkeit	Prozent	Gültige Prozente	Kumulierte Prozente
	57,00	1	1,3	2,3	88,6
	58,00	2	2,5	4,5	93,2
	59,00	1	1,3	2,3	95,5
	60,00	1	1,3	2,3	97,7
	61,00	1	1,3	2,3	100,0
	Gesamt	44	55,0	100,0	
Fehlend	System	36	45,0		
Gesamt		80	100,0		

```
GLM PSSersterZP PSSzweiterZP BY STAITraitMedianaufteilungErsterZP
  /WSFACTOR=Faktor1 2 Polynomial
  /METHOD=SSTYPE(3)
  /PLOT=PROFILE(Faktor1*STAITraitMedianaufteilungErsterZP) TYPE=LINE ERRORBAR=CI MEANR
EFERENCE=NO
    YAXIS=AUTO
  /PRINT=DESCRIPTIVE ETASQ OPOWER PARAMETER
  /PLOT=SPREADLEVEL RESIDUALS
  /CRITERIA=ALPHA(.05)
  /WSDESIGN=Faktor1
  /DESIGN=STAITraitMedianaufteilungErsterZP.
```

Allgemeines lineares Modell

Innersubjektfaktoren

Maß: MASS_1

Faktor1	Abhängige Variable
1	PSSersterZP
2	PSSzweiterZP

Zwischensubjektfaktoren

		Wertbeschriftung	N
STAITraitMedianaufteilungErsterZP	1,00	1bis40	13
	2,00	ab41	14

Deskriptive Statistiken

	STAITraitMedianaufteilung ErsterZP	Mittelwert	Standardabweichung	N
PSSersterZP	1bis40	16,0000	4,41588	13
	ab41	25,1429	4,81755	14
	Gesamt	20,7407	6,50203	27
PSSzweiterZP	1bis40	14,6923	4,62574	13
	ab41	24,0714	5,13606	14
	Gesamt	19,5556	6,77287	27

Multivariate Tests[a]

Effekt		Wert	F	Hypothese df
Faktor1	Pillai-Spur	,151	4,464[b]	1,000
	Wilks-Lambda	,849	4,464[b]	1,000
	Hotelling-Spur	,179	4,464[b]	1,000
	Größte charakteristische Wurzel nach Roy	,179	4,464[b]	1,000
Faktor1 * STAITraitMedianaufteilung ErsterZP	Pillai-Spur	,002	,044[b]	1,000
	Wilks-Lambda	,998	,044[b]	1,000
	Hotelling-Spur	,002	,044[b]	1,000
	Größte charakteristische Wurzel nach Roy	,002	,044[b]	1,000

Multivariate Tests[a]

Effekt		Fehler df	Sig.	Partielles Eta-Quadrat
Faktor1	Pillai-Spur	25,000	,045	,151
	Wilks-Lambda	25,000	,045	,151
	Hotelling-Spur	25,000	,045	,151
	Größte charakteristische Wurzel nach Roy	25,000	,045	,151
Faktor1 * STAITraitMedianaufteilung ErsterZP	Pillai-Spur	25,000	,836	,002
	Wilks-Lambda	25,000	,836	,002
	Hotelling-Spur	25,000	,836	,002
	Größte charakteristische Wurzel nach Roy	25,000	,836	,002

Multivariate Tests[a]

Effekt		Dezentr. Parameter	Beobachtete Trennschärfe[c]
Faktor1	Pillai-Spur	4,464	,528
	Wilks-Lambda	4,464	,528
	Hotelling-Spur	4,464	,528
	Größte charakteristische Wurzel nach Roy	4,464	,528
Faktor1 * STAITraitMedianaufteilung ErsterZP	Pillai-Spur	,044	,055
	Wilks-Lambda	,044	,055
	Hotelling-Spur	,044	,055
	Größte charakteristische Wurzel nach Roy	,044	,055

a. Design: Konstanter Term + STAITraitMedianaufteilungErsterZP
Innersubjektdesign: Faktor1

b. Exakte Statistik

c. Unter Verwendung von Alpha = ,05 berechnet

Mauchly-Test auf Sphärizität[a]

Maß: MASS_1

Innersubjekteffekt	Mauchly-W	Ungefähres Chi-Quadrat	df	Sig.	Epsilon[b] Greenhouse-Geisser
Faktor1	1,000	,000	0	.	1,000

Mauchly-Test auf Sphärizität[a]

Maß: MASS_1

Innersubjekteffekt	Epsilon[b] Huynh-Feldt (HF)	Untergrenze
Faktor1	1,000	1,000

Prüft die Nullhypothese, dass sich die Fehlerkovarianz-Matrix der orthonormalisierten transformierten abhängigen Variablen proportional zur Einheitsmatrix verhält.

a. Design: Konstanter Term + STAITraitMedianaufteilungErsterZP
Innersubjektdesign: Faktor1

b. Kann zum Korrigieren der Freiheitsgrade für die gemittelten Signifikanztests verwendet werden. In der Tabelle mit den Tests der Effekte innerhalb der Subjekte werden korrigierte Tests angezeigt.

Tests der Innersubjekteffekte

Maß: MASS_1

Quelle		Typ III Quadratsumme	df	Mittel der Quadrate
Faktor1	Sphärizität angenommen	19,077	1	19,077
	Greenhouse-Geisser	19,077	1,000	19,077
	Huynh-Feldt (HF)	19,077	1,000	19,077
	Untergrenze	19,077	1,000	19,077
Faktor1 * STAITraitMedianaufteilung ErsterZP	Sphärizität angenommen	,188	1	,188
	Greenhouse-Geisser	,188	1,000	,188
	Huynh-Feldt (HF)	,188	1,000	,188
	Untergrenze	,188	1,000	,188
Fehler(Faktor1)	Sphärizität angenommen	106,849	25	4,274
	Greenhouse-Geisser	106,849	25,000	4,274
	Huynh-Feldt (HF)	106,849	25,000	4,274
	Untergrenze	106,849	25,000	4,274

Tests der Innersubjekteffekte

Maß: MASS_1

Quelle		F	Sig.	Partielles Eta-Quadrat
Faktor1	Sphärizität angenommen	4,464	,045	,151
	Greenhouse-Geisser	4,464	,045	,151
	Huynh-Feldt (HF)	4,464	,045	,151
	Untergrenze	4,464	,045	,151
Faktor1 * STAITraitMedianaufteilung ErsterZP	Sphärizität angenommen	,044	,836	,002
	Greenhouse-Geisser	,044	,836	,002
	Huynh-Feldt (HF)	,044	,836	,002
	Untergrenze	,044	,836	,002
Fehler(Faktor1)	Sphärizität angenommen			
	Greenhouse-Geisser			
	Huynh-Feldt (HF)			
	Untergrenze			

Tests der Innersubjekteffekte

Maß: MASS_1

Quelle		Dezentr. Parameter	Beobachtete Trennschärfe[a]
Faktor1	Sphärizität angenommen	4,464	,528
	Greenhouse-Geisser	4,464	,528
	Huynh-Feldt (HF)	4,464	,528
	Untergrenze	4,464	,528
Faktor1 * STAITraitMedianaufteilung ErsterZP	Sphärizität angenommen	,044	,055
	Greenhouse-Geisser	,044	,055
	Huynh-Feldt (HF)	,044	,055
	Untergrenze	,044	,055
Fehler(Faktor1)	Sphärizität angenommen		
	Greenhouse-Geisser		
	Huynh-Feldt (HF)		
	Untergrenze		

a. Unter Verwendung von Alpha = ,05 berechnet

Tests der Innersubjektkontraste

Maß: MASS_1

Quelle	Faktor1	Typ III Quadratsumme	df	Mittel der Quadrate	F
Faktor1	Linear	19,077	1	19,077	4,464
Faktor1 * STAITraitMedianaufteilung ErsterZP	Linear	,188	1	,188	,044
Fehler(Faktor1)	Linear	106,849	25	4,274	

Tests der Innersubjektkontraste

Maß: MASS_1

Quelle	Faktor1	Sig.	Partielles Eta-Quadrat	Dezentr. Parameter	Beobachtete Trennschärfe[a]
Faktor1	Linear	,045	,151	4,464	,528
Faktor1 * STAITraitMedianaufteilung ErsterZP	Linear	,836	,002	,044	,055
Fehler(Faktor1)	Linear				

a. Unter Verwendung von Alpha = ,05 berechnet

Tests der Zwischensubjekteffekte

Maß: MASS_1

Transformierte Variable: Mittel

Quelle	Typ III Quadratsumme	df	Mittel der Quadrate	F	Sig.
Konstanter Term	21520,029	1	21520,029	523,060	,000
STAITraitMedianaufteilung ErsterZP	1156,252	1	1156,252	28,104	,000
Fehler	1028,563	25	41,143		

Tests der Zwischensubjekteffekte

Maß: MASS_1

Transformierte Variable: Mittel

Quelle	Partielles Eta-Quadrat	Dezentr. Parameter	Beobachtete Trennschärfe[a]
Konstanter Term	,954	523,060	1,000
STAITraitMedianaufteilung ErsterZP	,529	28,104	,999
Fehler			

a. Unter Verwendung von Alpha = ,05 berechnet

Parameterschätzungen

Abhängige Variable	Parameter	RegressionskoeffizientB	Std.-Fehler	T	Sig.
PSSersterZP	Konstanter Term	25,143	1,237	20,323	,000
	[STAITraitMedianaufteilung ErsterZP=1,00]	-9,143	1,783	-5,128	,000
	[STAITraitMedianaufteilung ErsterZP=2,00]	0[a]	.	.	.
PSSzweiterZP	Konstanter Term	24,071	1,309	18,389	,000
	[STAITraitMedianaufteilung ErsterZP=1,00]	-9,379	1,886	-4,972	,000
	[STAITraitMedianaufteilung ErsterZP=2,00]	0[a]	.	.	.

Parameterschätzungen

Abhängige Variable	Parameter	95% Konfidenzintervall Untergrenze	95% Konfidenzintervall Obergrenze	Partielles Eta-Quadrat
PSSersterZP	Konstanter Term	22,595	27,691	,943
	[STAITraitMedianaufteilung ErsterZP=1,00]	-12,815	-5,471	,513
	[STAITraitMedianaufteilung ErsterZP=2,00]	.	.	.
PSSzweiterZP	Konstanter Term	21,376	26,767	,931
	[STAITraitMedianaufteilung ErsterZP=1,00]	-13,264	-5,494	,497
	[STAITraitMedianaufteilung ErsterZP=2,00]	.	.	.

Parameterschätzungen

Abhängige Variable	Parameter	Dezentr. Parameter	Beobachtete Trennschärfe[b]
PSSersterZP	Konstanter Term	20,323	1,000
	[STAITraitMedianaufteilung ErsterZP=1,00]	5,128	,998
	[STAITraitMedianaufteilung ErsterZP=2,00]	.	.
PSSzweiterZP	Konstanter Term	18,389	1,000
	[STAITraitMedianaufteilung ErsterZP=1,00]	4,972	,998
	[STAITraitMedianaufteilung ErsterZP=2,00]	.	.

a. Dieser Parameter wird auf null gesetzt, da er redundant ist.

b. Unter Verwendung von Alpha = ,05 berechnet

Diagramm\: Streubreite gegen mittleres Niveau

Standardabweichungen gegen Mittelwerte

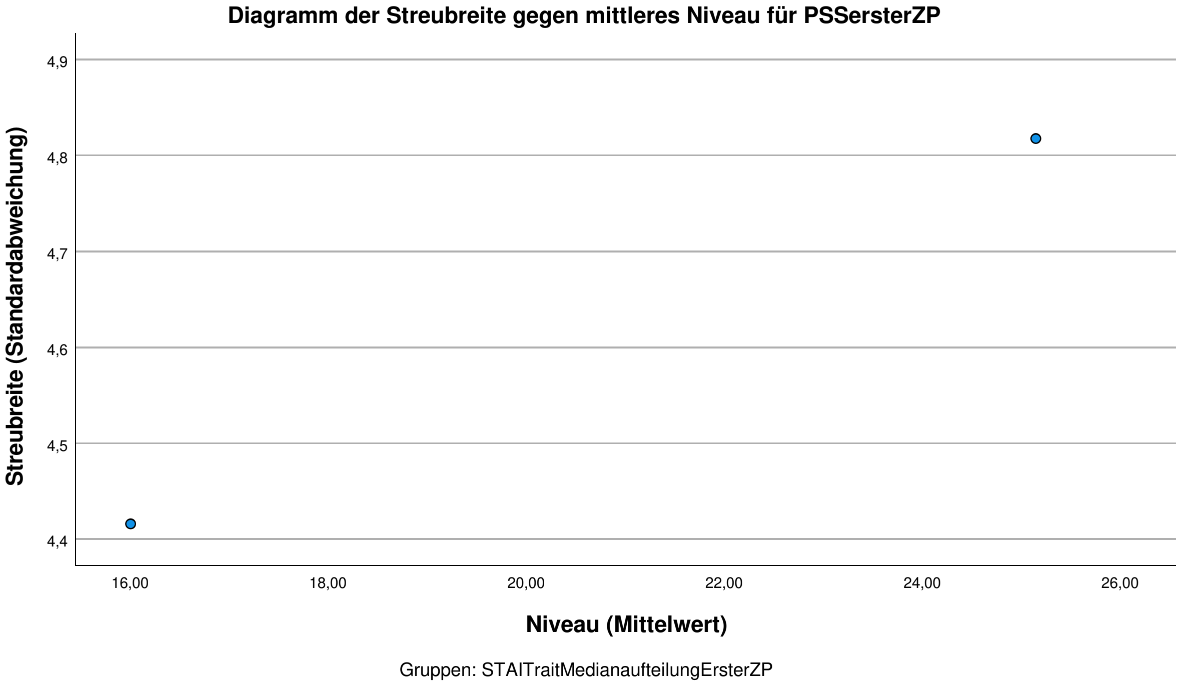

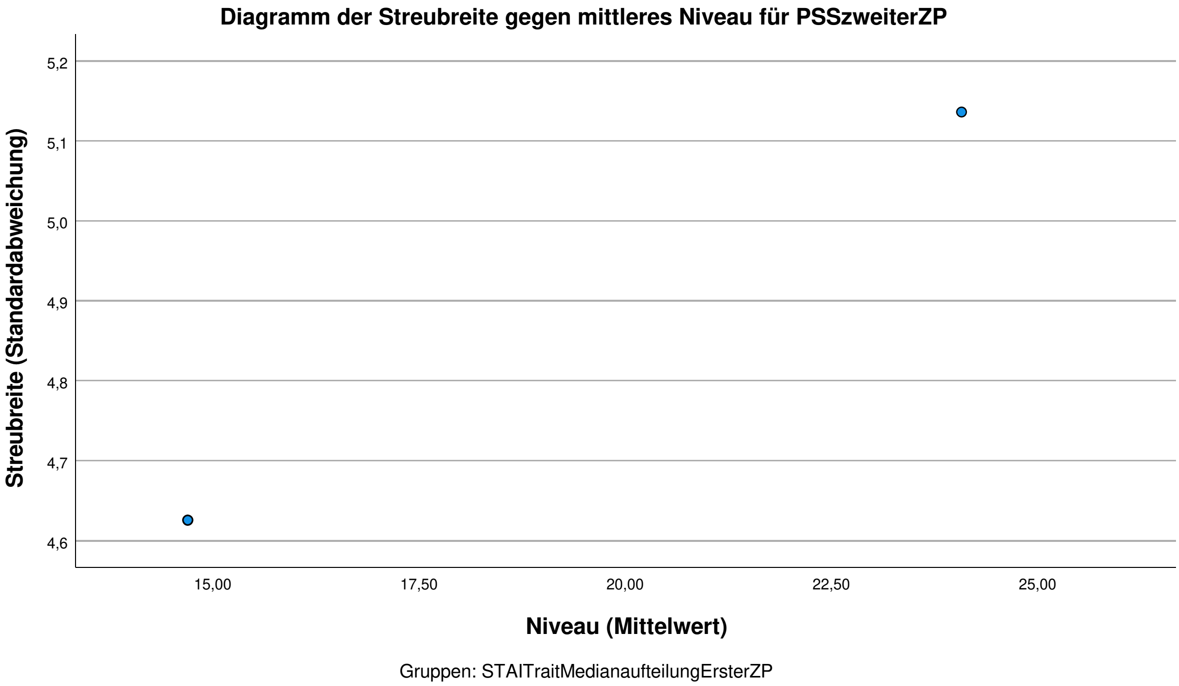

Varianzen gegen Mittelwerte

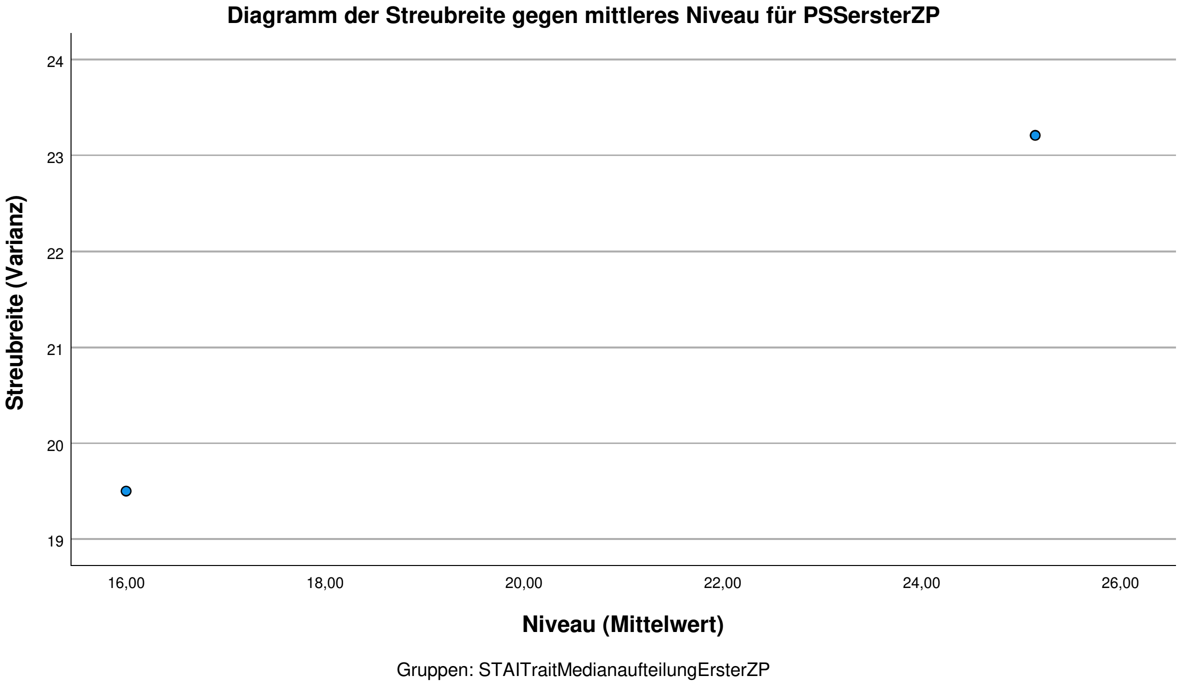

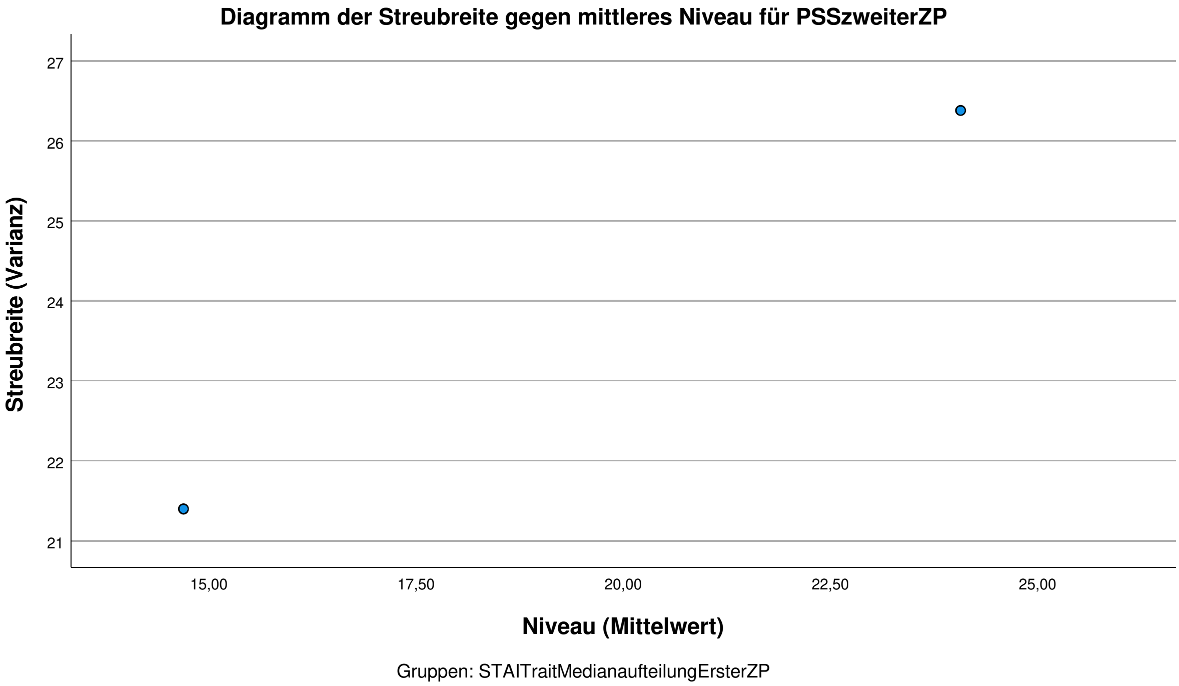

Residuen-Diagramme: Beobachtet * Vorhergesagt * Std.

Abhängige Variable: PSSersterZP

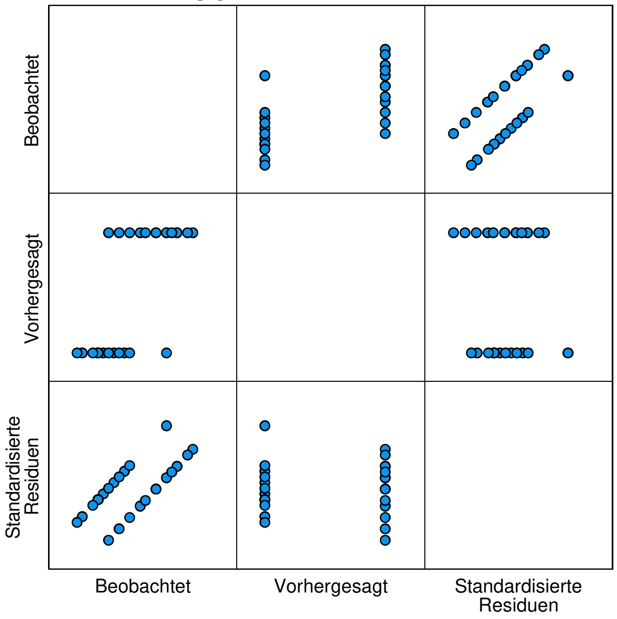

Modell: Konstanter Term + STAITraitMedianaufteilungErsterZP

Abhängige Variable: PSSzweiterZP

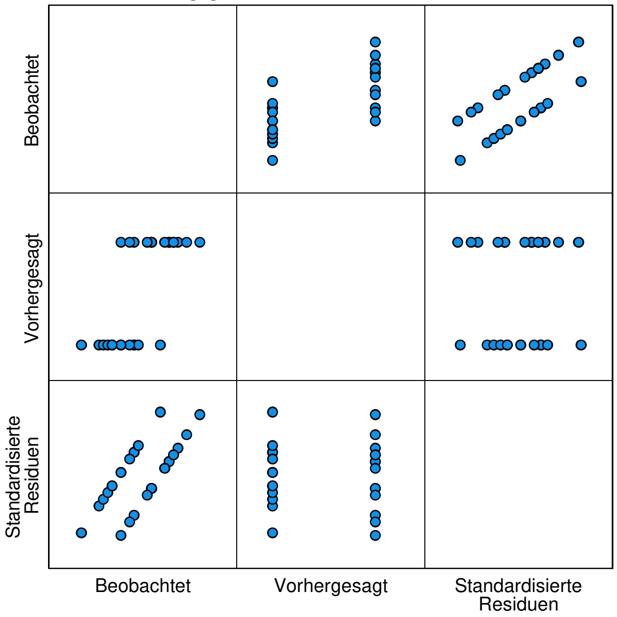

Modell: Konstanter Term + STAITraitMedianaufteilungErsterZP

Profilplots

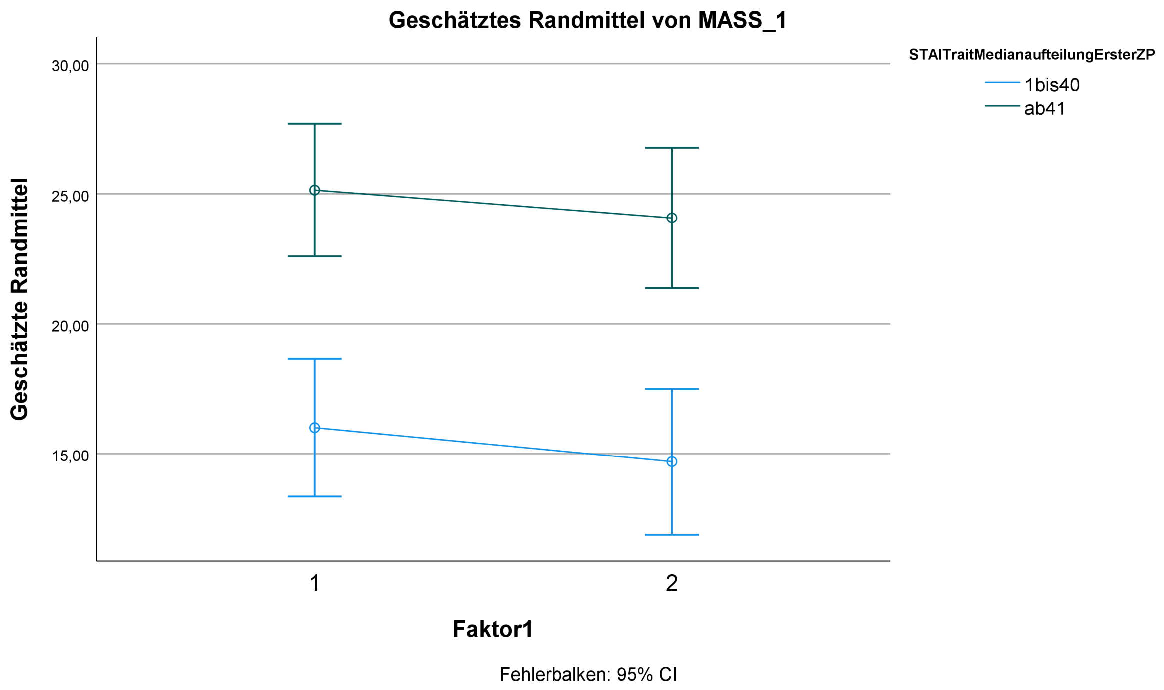


```
GLM PSSersterZP PSSzweiterZP BY STAITraitMedianaufteilungErsterZP WITH BRCSersterZP
  /WSFACTOR=Faktor1 2 Polynomial
  /METHOD=SSTYPE(3)
  /PLOT=PROFILE(Faktor1*STAITraitMedianaufteilungErsterZP) TYPE=LINE ERRORBAR=CI MEA
EFERENCE=NO
    YAXIS=AUTO
  /PRINT=DESCRIPTIVE ETASQ OPOWER PARAMETER
  /PLOT=SPREADLEVEL RESIDUALS
  /CRITERIA=ALPHA(.05)
  /WSDESIGN=Faktor1
  /DESIGN=BRCSersterZP STAITraitMedianaufteilungErsterZP.
```

Allgemeines lineares Modell

Innersubjektfaktoren

Maß: MASS_1

Faktor1	Abhängige Variable
1	PSSersterZP
2	PSSzweiterZP

Zwischensubjektfaktoren

		Wertbeschriftung	N
STAITraitMedianaufteilungErsterZP	1,00	1bis40	13
	2,00	ab41	14

Deskriptive Statistiken

	STAITraitMedianaufteilung ErsterZP	Mittelwert	Standardabweichung	N
PSSersterZP	1bis40	16,0000	4,41588	13
	ab41	25,1429	4,81755	14
	Gesamt	20,7407	6,50203	27
PSSzweiterZP	1bis40	14,6923	4,62574	13
	ab41	24,0714	5,13606	14
	Gesamt	19,5556	6,77287	27

Multivariate Tests[a]

Effekt		Wert	F	Hypothese df
Faktor1	Pillai-Spur	,004	,091[b]	1,000
	Wilks-Lambda	,996	,091[b]	1,000
	Hotelling-Spur	,004	,091[b]	1,000
	Größte charakteristische Wurzel nach Roy	,004	,091[b]	1,000
Faktor1 * BRCSersterZP	Pillai-Spur	,027	,661[b]	1,000
	Wilks-Lambda	,973	,661[b]	1,000
	Hotelling-Spur	,028	,661[b]	1,000
	Größte charakteristische Wurzel nach Roy	,028	,661[b]	1,000
Faktor1 * STAITraitMedianaufteilung ErsterZP	Pillai-Spur	,000	,003[b]	1,000
	Wilks-Lambda	1,000	,003[b]	1,000
	Hotelling-Spur	,000	,003[b]	1,000
	Größte charakteristische Wurzel nach Roy	,000	,003[b]	1,000

Multivariate Tests[a]

Effekt		Fehler df	Sig.	Partielles Eta-Quadrat
Faktor1	Pillai-Spur	24,000	,765	,004
	Wilks-Lambda	24,000	,765	,004
	Hotelling-Spur	24,000	,765	,004
	Größte charakteristische Wurzel nach Roy	24,000	,765	,004
Faktor1 * BRCSersterZP	Pillai-Spur	24,000	,424	,027
	Wilks-Lambda	24,000	,424	,027
	Hotelling-Spur	24,000	,424	,027
	Größte charakteristische Wurzel nach Roy	24,000	,424	,027
Faktor1 * STAITraitMedianaufteilung ErsterZP	Pillai-Spur	24,000	,958	,000
	Wilks-Lambda	24,000	,958	,000
	Hotelling-Spur	24,000	,958	,000
	Größte charakteristische Wurzel nach Roy	24,000	,958	,000

Multivariate Tests[a]

Effekt		Dezentr. Parameter	Beobachtete Trennschärfe[c]
Faktor1	Pillai-Spur	,091	,060
	Wilks-Lambda	,091	,060
	Hotelling-Spur	,091	,060
	Größte charakteristische Wurzel nach Roy	,091	,060
Faktor1 * BRCSersterZP	Pillai-Spur	,661	,122
	Wilks-Lambda	,661	,122
	Hotelling-Spur	,661	,122
	Größte charakteristische Wurzel nach Roy	,661	,122
Faktor1 * STAITraitMedianaufteilung ErsterZP	Pillai-Spur	,003	,050
	Wilks-Lambda	,003	,050
	Hotelling-Spur	,003	,050
	Größte charakteristische Wurzel nach Roy	,003	,050

a. Design: Konstanter Term + BRCSersterZP + STAITraitMedianaufteilungErsterZP
Innersubjektdesign: Faktor1

b. Exakte Statistik

c. Unter Verwendung von Alpha = ,05 berechnet

Mauchly-Test auf Sphärizität[a]

Maß: MASS_1

Innersubjekteffekt	Mauchly-W	Ungefähres Chi-Quadrat	df	Sig.	Epsilon[b] Greenhouse-Geisser
Faktor1	1,000	,000	0	.	1,000

Mauchly-Test auf Sphärizität[a]

Maß: MASS_1

Innersubjekteffekt	Epsilon[b] Huynh-Feldt (HF)	Untergrenze
Faktor1	1,000	1,000

Prüft die Nullhypothese, dass sich die Fehlerkovarianz-Matrix der orthonormalisierten transformierten abhängigen Variablen proportional zur Einheitsmatrix verhält.

a. Design: Konstanter Term + BRCSersterZP + STAITraitMedianaufteilungErsterZP
Innersubjektdesign: Faktor1

b. Kann zum Korrigieren der Freiheitsgrade für die gemittelten Signifikanztests verwendet werden. In der Tabelle mit den Tests der Effekte innerhalb der Subjekte werden korrigierte Tests angezeigt.

Tests der Innersubjekteffekte

Maß: MASS_1

Quelle		Typ III Quadratsumme	df	Mittel der Quadrate
Faktor1	Sphärizität angenommen	,396	1	,396
	Greenhouse-Geisser	,396	1,000	,396
	Huynh-Feldt (HF)	,396	1,000	,396
	Untergrenze	,396	1,000	,396
Faktor1 * BRCSersterZP	Sphärizität angenommen	2,863	1	2,863
	Greenhouse-Geisser	2,863	1,000	2,863
	Huynh-Feldt (HF)	2,863	1,000	2,863
	Untergrenze	2,863	1,000	2,863
Faktor1 * STAITraitMedianaufteilung ErsterZP	Sphärizität angenommen	,012	1	,012
	Greenhouse-Geisser	,012	1,000	,012
	Huynh-Feldt (HF)	,012	1,000	,012
	Untergrenze	,012	1,000	,012
Fehler(Faktor1)	Sphärizität angenommen	103,986	24	4,333
	Greenhouse-Geisser	103,986	24,000	4,333
	Huynh-Feldt (HF)	103,986	24,000	4,333
	Untergrenze	103,986	24,000	4,333

Tests der Innersubjekteffekte

Maß: MASS_1

Quelle		F	Sig.	Partielles Eta-Quadrat
Faktor1	Sphärizität angenommen	,091	,765	,004
	Greenhouse-Geisser	,091	,765	,004
	Huynh-Feldt (HF)	,091	,765	,004
	Untergrenze	,091	,765	,004
Faktor1 * BRCSersterZP	Sphärizität angenommen	,661	,424	,027
	Greenhouse-Geisser	,661	,424	,027
	Huynh-Feldt (HF)	,661	,424	,027
	Untergrenze	,661	,424	,027
Faktor1 * STAITraitMedianaufteilung ErsterZP	Sphärizität angenommen	,003	,958	,000
	Greenhouse-Geisser	,003	,958	,000
	Huynh-Feldt (HF)	,003	,958	,000
	Untergrenze	,003	,958	,000
Fehler(Faktor1)	Sphärizität angenommen			
	Greenhouse-Geisser			
	Huynh-Feldt (HF)			
	Untergrenze			

Tests der Innersubjekteffekte

Maß: MASS_1

Quelle		Dezentr. Parameter	Beobachtete Trennschärfe[a]
Faktor1	Sphärizität angenommen	,091	,060
	Greenhouse-Geisser	,091	,060
	Huynh-Feldt (HF)	,091	,060
	Untergrenze	,091	,060
Faktor1 * BRCSersterZP	Sphärizität angenommen	,661	,122
	Greenhouse-Geisser	,661	,122
	Huynh-Feldt (HF)	,661	,122
	Untergrenze	,661	,122
Faktor1 * STAITraitMedianaufteilung ErsterZP	Sphärizität angenommen	,003	,050
	Greenhouse-Geisser	,003	,050
	Huynh-Feldt (HF)	,003	,050
	Untergrenze	,003	,050
Fehler(Faktor1)	Sphärizität angenommen		
	Greenhouse-Geisser		
	Huynh-Feldt (HF)		
	Untergrenze		

a. Unter Verwendung von Alpha = ,05 berechnet

Tests der Innersubjektkontraste

Maß: MASS_1

Quelle	Faktor1	Typ III Quadratsumme	df	Mittel der Quadrate	F
Faktor1	Linear	,396	1	,396	,091
Faktor1 * BRCSersterZP	Linear	2,863	1	2,863	,661
Faktor1 * STAITraitMedianaufteilung ErsterZP	Linear	,012	1	,012	,003
Fehler(Faktor1)	Linear	103,986	24	4,333	

Tests der Innersubjektkontraste

Maß: MASS_1

Quelle	Faktor1	Sig.	Partielles Eta-Quadrat	Dezentr. Parameter	Beobachtete Trennschärfe[a]
Faktor1	Linear	,765	,004	,091	,060
Faktor1 * BRCSersterZP	Linear	,424	,027	,661	,122
Faktor1 * STAITraitMedianaufteilung ErsterZP	Linear	,958	,000	,003	,050
Fehler(Faktor1)	Linear				

a. Unter Verwendung von Alpha = ,05 berechnet

Tests der Zwischensubjekteffekte

Maß: MASS_1

Transformierte Variable: Mittel

Quelle	Typ III Quadratsumme	df	Mittel der Quadrate	F	Sig.
Konstanter Term	1740,330	1	1740,330	43,159	,000
BRCSersterZP	60,794	1	60,794	1,508	,231
STAITraitMedianaufteilung ErsterZP	1021,128	1	1021,128	25,323	,000
Fehler	967,770	24	40,324		

Tests der Zwischensubjekteffekte

Maß: MASS_1

Transformierte Variable: Mittel

Quelle	Partielles Eta-Quadrat	Dezentr. Parameter	Beobachtete Trennschärfe[a]
Konstanter Term	,643	43,159	1,000
BRCSersterZP	,059	1,508	,218
STAITraitMedianaufteilung ErsterZP	,513	25,323	,998
Fehler			

a. Unter Verwendung von Alpha = ,05 berechnet

Parameterschätzungen

Abhängige Variable	Parameter	Regressionskoeffizient B	Std.-Fehler	T	Sig.
PSSersterZP	Konstanter Term	28,460	3,776	7,537	,000
	BRCSersterZP	-,261	,281	-,930	,362
	[STAITraitMedianaufteilung ErsterZP=1,00]	-8,827	1,820	-4,851	,000
	[STAITraitMedianaufteilung ErsterZP=2,00]	0[a]	.	.	.
PSSzweiterZP	Konstanter Term	29,227	3,911	7,473	,000
	BRCSersterZP	-,406	,291	-1,396	,176
	[STAITraitMedianaufteilung ErsterZP=1,00]	-8,889	1,885	-4,716	,000
	[STAITraitMedianaufteilung ErsterZP=2,00]	0[a]	.	.	.

Parameterschätzungen

Abhängige Variable	Parameter	95% Konfidenzintervall		Partielles Eta-Quadrat
		Untergrenze	Obergrenze	
PSSersterZP	Konstanter Term	20,666	36,254	,703
	BRCSersterZP	-,840	,318	,035
	[STAITraitMedianaufteilung ErsterZP=1,00]	-12,583	-5,072	,495
	[STAITraitMedianaufteilung ErsterZP=2,00]	.	.	.
PSSzweiterZP	Konstanter Term	21,155	37,299	,699
	BRCSersterZP	-1,005	,194	,075
	[STAITraitMedianaufteilung ErsterZP=1,00]	-12,779	-4,999	,481
	[STAITraitMedianaufteilung ErsterZP=2,00]	.	.	.

Parameterschätzungen

Abhängige Variable	Parameter	Dezentr. Parameter	Beobachtete Trennschärfe[b]
PSSersterZP	Konstanter Term	7,537	1,000
	BRCSersterZP	,930	,145
	[STAITraitMedianaufteilungErsterZP=1,00]	4,851	,996
	[STAITraitMedianaufteilungErsterZP=2,00]	.	.
PSSzweiterZP	Konstanter Term	7,473	1,000
	BRCSersterZP	1,396	,268
	[STAITraitMedianaufteilungErsterZP=1,00]	4,716	,995
	[STAITraitMedianaufteilungErsterZP=2,00]	.	.

a. Dieser Parameter wird auf null gesetzt, da er redundant ist.

b. Unter Verwendung von Alpha = ,05 berechnet

Diagramm\: Streubreite gegen mittleres Niveau

Standardabweichungen gegen Mittelwerte

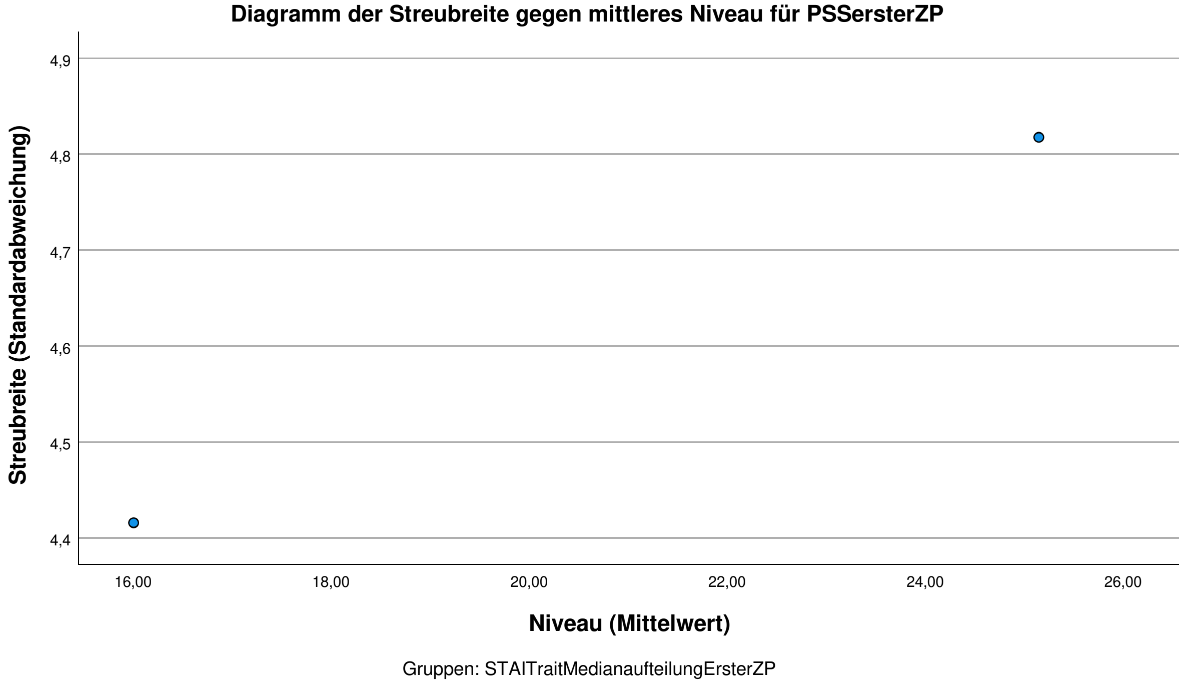

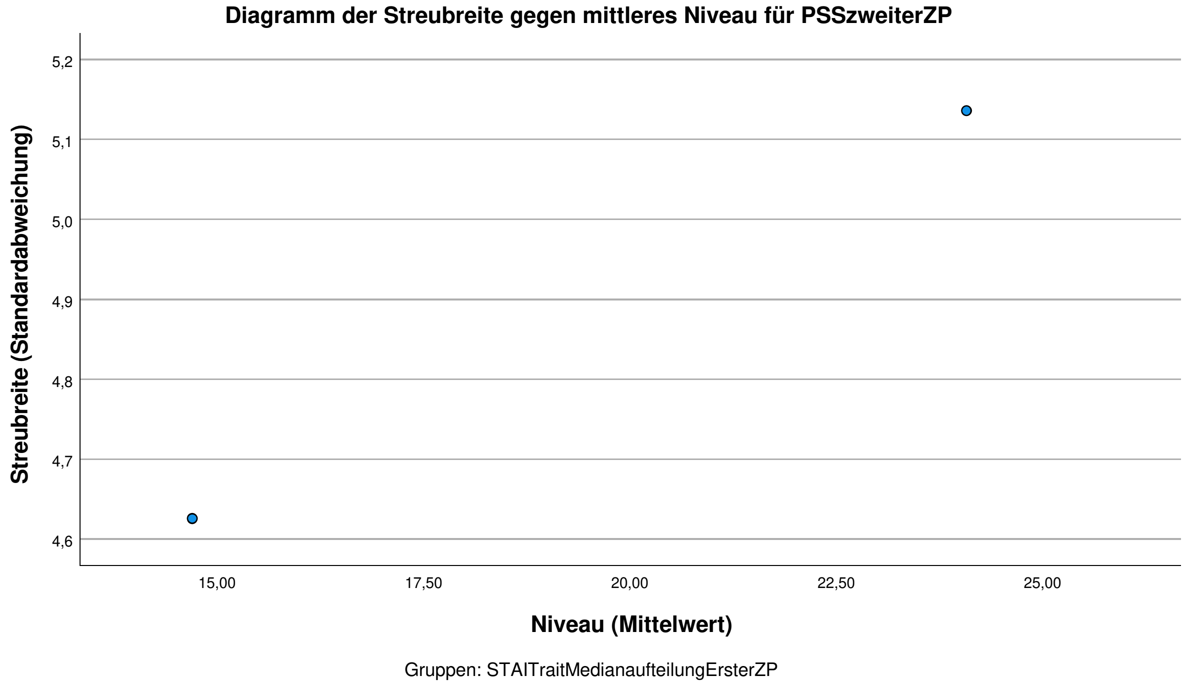

Varianzen gegen Mittelwerte

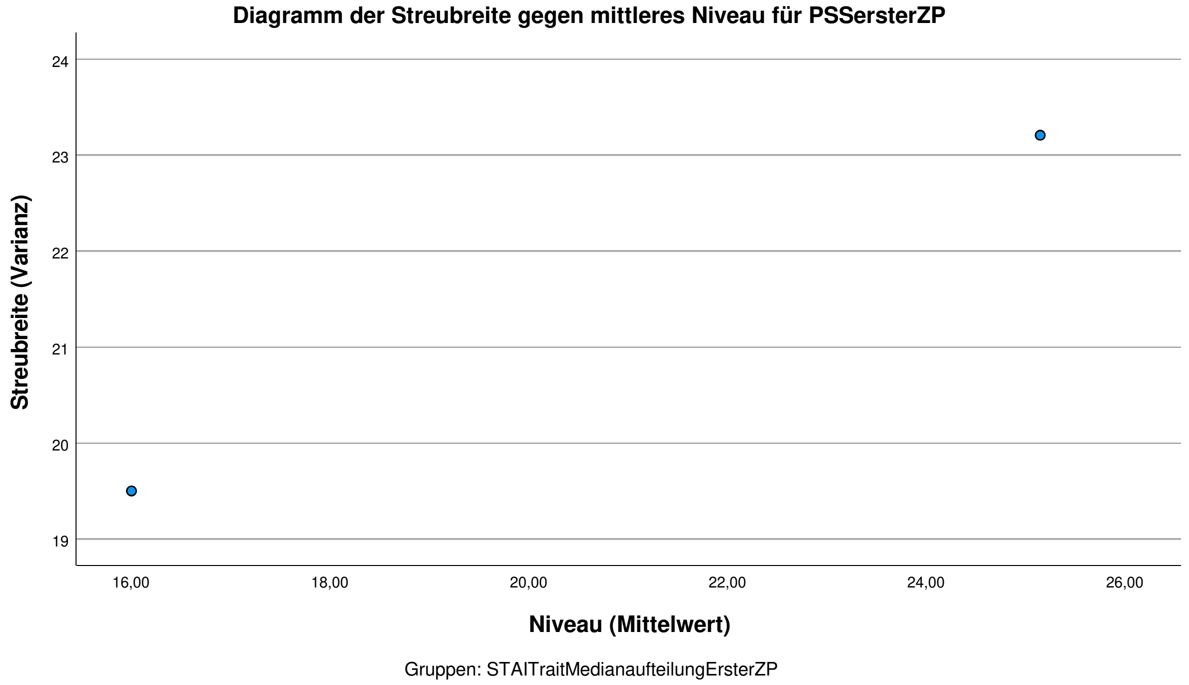

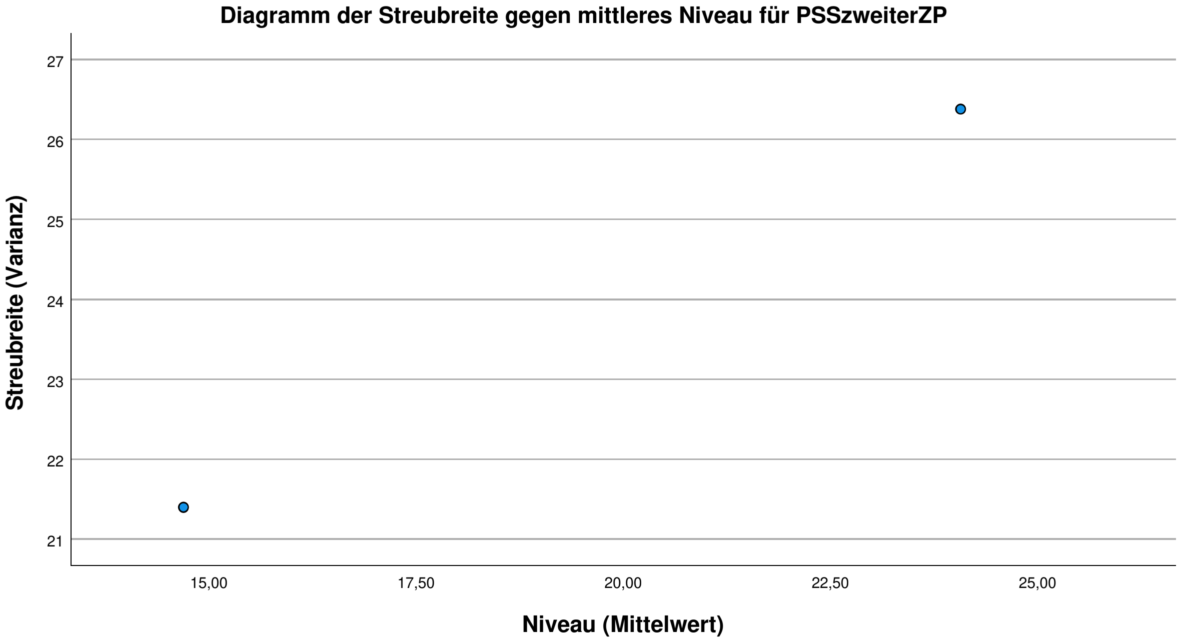

Gruppen: STAITraitMedianaufteilungErsterZP

Residuen-Diagramme: Beobachtet * Vorhergesagt * Std.

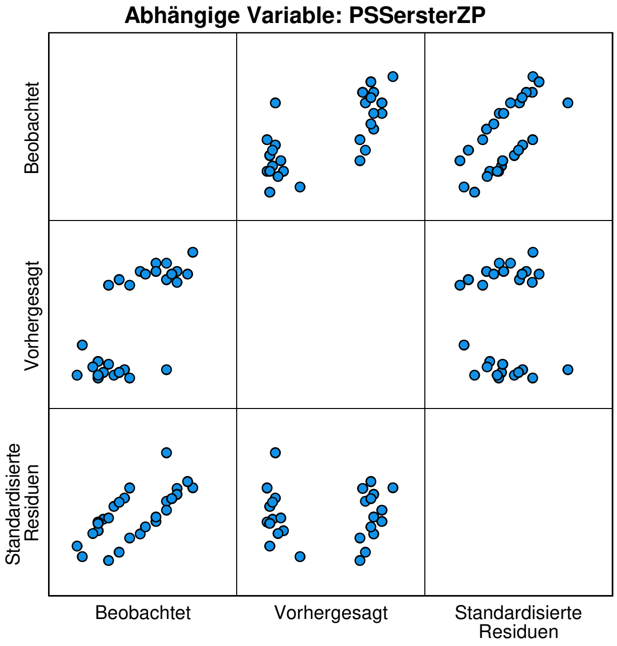

Modell: Konstanter Term + BRCSersterZP + STAITraitMedianaufteilungErsterZP

Abhängige Variable: PSSzweiterZP

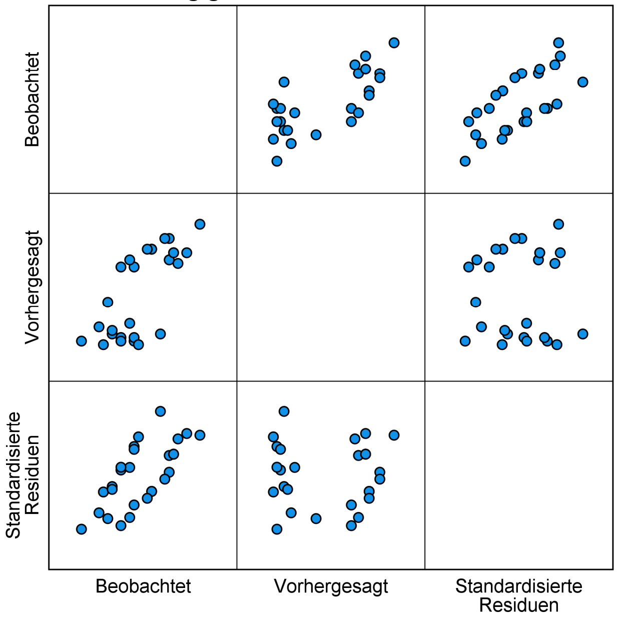

Modell: Konstanter Term + BRCSersterZP + STAITraitMedianaufteilungErsterZP

Profilplots

Geschätztes Randmittel von MASS_1

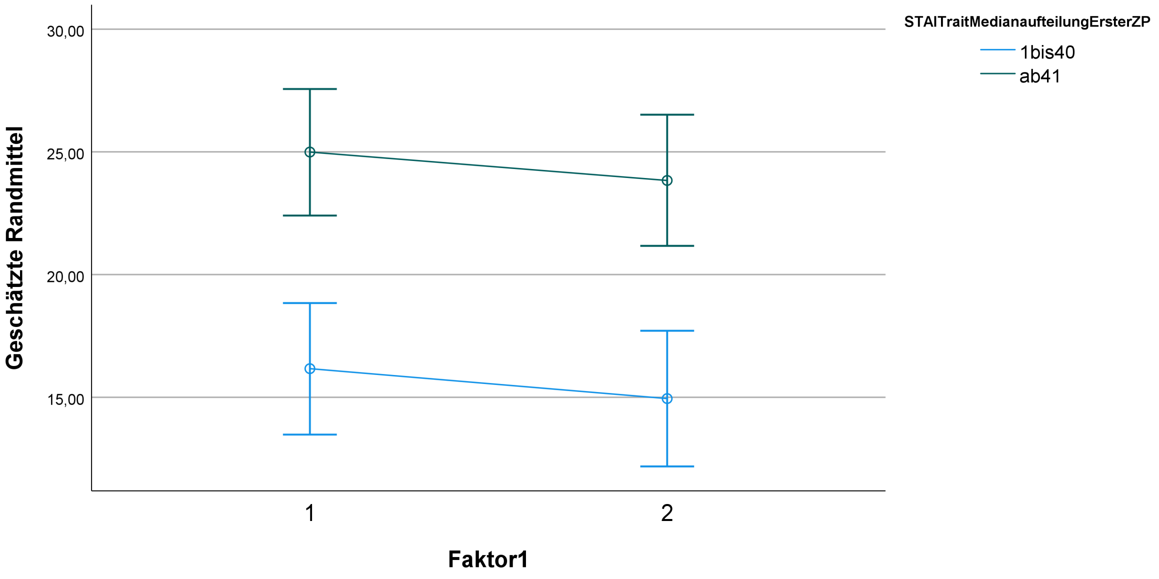

Die Kovariaten im Modell werden anhand der folgenden Werte berechnet: BRCSersterZP = 13,2963

Fehlerbalken: 95% CI
